# Supplementary material for: Distributed information encoding and decoding using self-organized spatial patterns
Source: Patterns (N Y). 2022 Sep 23;3(10):100590. doi: 10.1016/j.patter.2022.100590 (PMC9583124; doi:10.1016/j.patter.2022.100590)
Supplement: Document S1. Figures S1–S15 and Tables S1 and S2 [file mmc1.pdf]

Patterns, Volume *3*

Supplemental information

Distributed information encoding and decoding using self-organized spatial patterns

Jia Lu, Ryan Tsoi, Nan Luo, Yuanchi Ha, Shangying Wang, Minjun Kwak, Yasa Baig, Nicole Moiseyev, Shari Tian, Alison Zhang, Neil Zhenqiang Gong, and Lingchong You

Supplementary Information

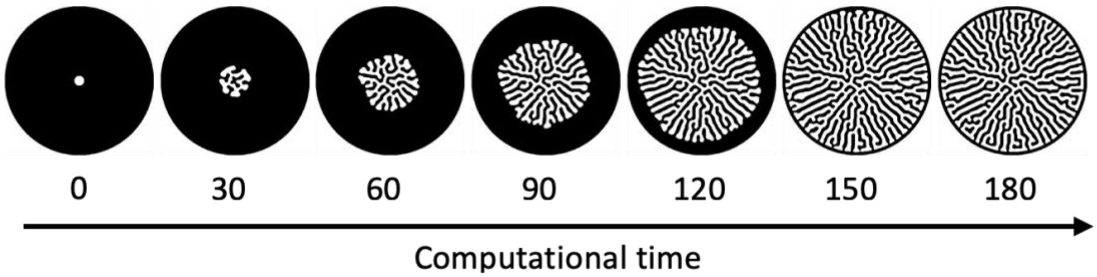

Fig. S1. Time series of branching pattern development.
An example of colony growth starting from a single spot configuration at the center of a circular growth domain. The cells are unevenly seeded within the initial configuration. As the colony grows, it develops into an intricate branching pattern and stops growing at around t = 180. The simulation parameters are the default: seeding spot radius = 5, $d_1/d_2 = 0.4$, $h_1 = 1000, h_2 = 2000, b = 6.5$, $\epsilon = 2000$, there was not growth noise (See "Methods").

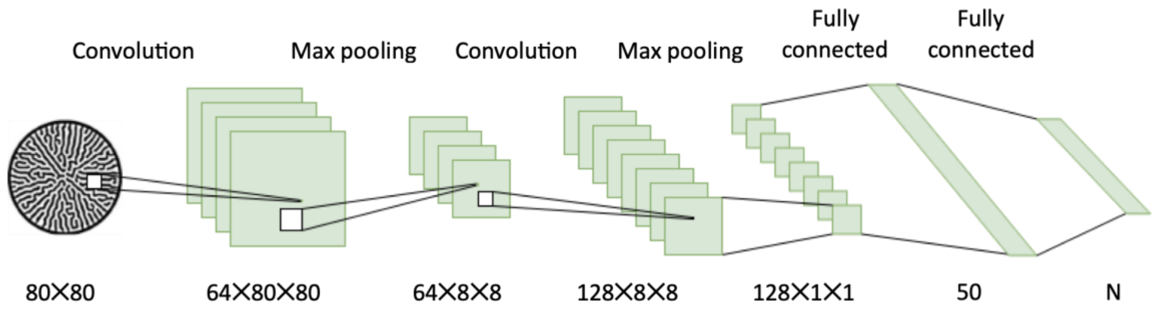

Fig. S2. Schematic of CNN decoder architecture.
The CNN takes an 80 × 80 greyscale image as input, and outputs a N dimensional feature, where N is the number of characters in a dictionary.

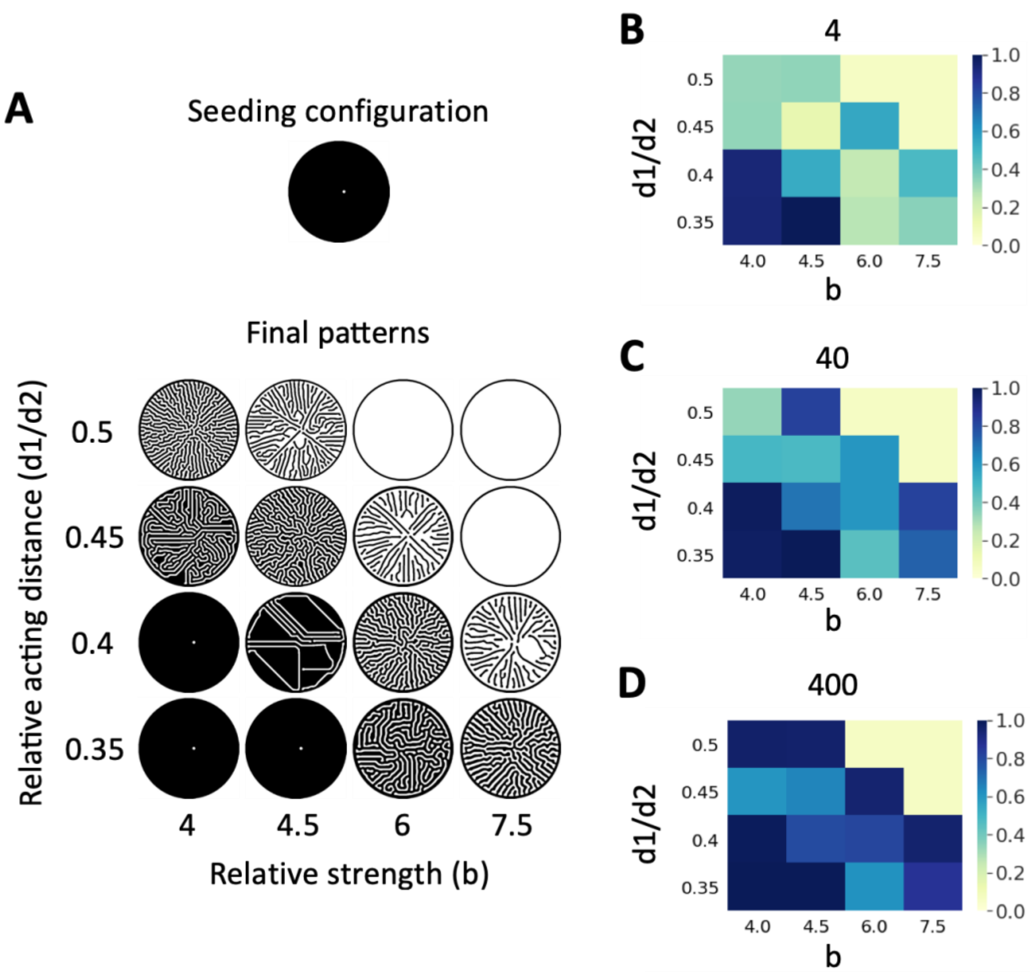

Fig. S3. Patterning dynamics impact the tradeoff among encoding capacity, security, and decoding reliability.

A. Starting from the same initial seeding configuration (and the same seeding noise), cells grow into diverse final patterns under different growth dynamics. Here, the growth dynamic is defined by two parameters: the relative strength and the relative acting distance of colony expansion and repulsion. The simulation is terminated when the growth stops. We can coarsely classify the patterns into three groups: the trivial (lower left corner), the disk-like (top right corner) and the branching patterns (the ones along the diagonal).

B – D. Decoding accuracy of different dynamics when scarce (B), intermediate (C), and adequate (D) training data are available (4, 40, and 400 replicates per class respectively). The trivial patterns always have high decoding capacity, whereas the disk-like patterns always have low decoding capacity. For branching patterns, the decoding accuracy increases drastically if more data become available.

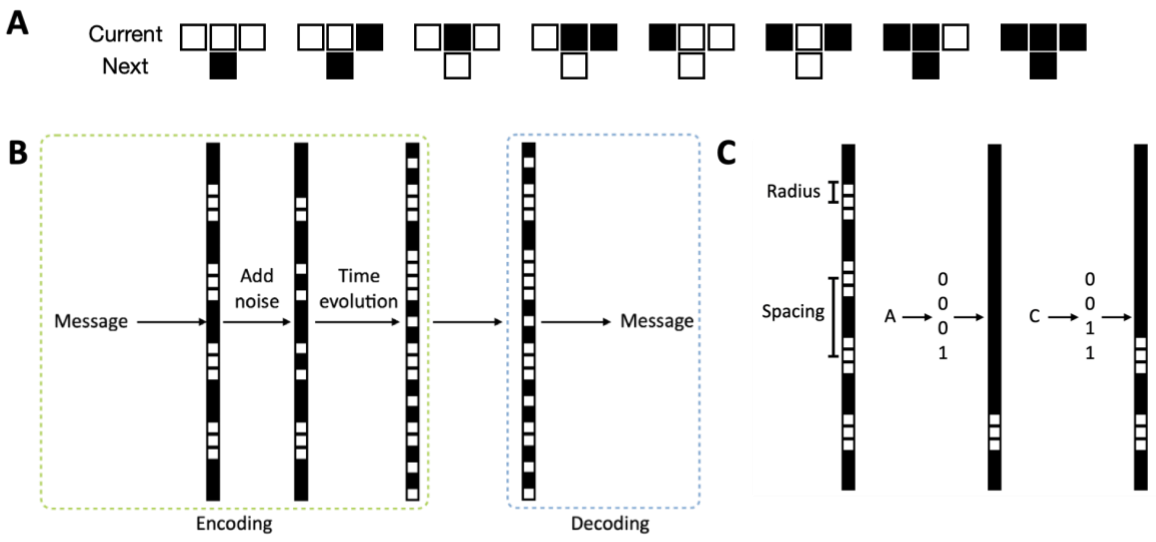

Fig. S4. Encoding and decoding using elementary cellular automata (ECA) with rule 60.
A. Rule 60 of ECA. Color indicates cell status: black – 0; white – 1. For the specific “Current” states, the sequence of the "Next” states represents the number 60 in a binary format (00111100).
B. Encoding-decoding scheme using one dimensional ECA. To encode, a message is first converted into a one-dimensional seeding array and then noise is added to it. The sequence then evolves into a final pattern following rule 60. A trained feedforward neural network is used to decode the pattern.
C. Predefined braille-like cell seeding arrangement and examples of encoded letters.

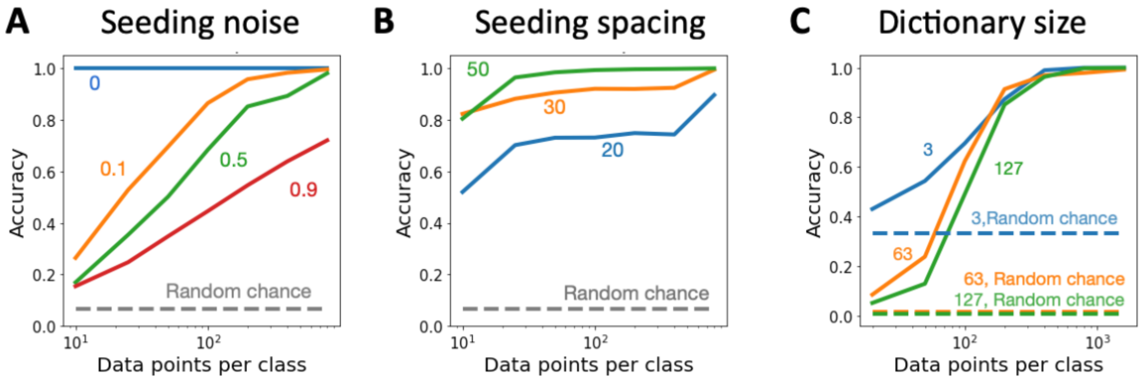

Fig. S5. Tradeoff between encoding capacity, security, and decoding reliability of the CA model. We varied parameters including: A. level of noise in time evolution (increasing value indicates higher noise), B. spacing between spots (time step = 600), and C. number of characters in encoding setup. The decoding accuracy generally increases as the number of replicates per class increases, and it significantly exceeds the corresponding accuracy by random guessing. Increasing complexity such as using larger growth noise or smaller spacing would require more data to reach the same accuracy. Larger dictionary size does not lead to sufficiently distinguishable training performance profile, it indicates every system may have different capacity and require tuning.

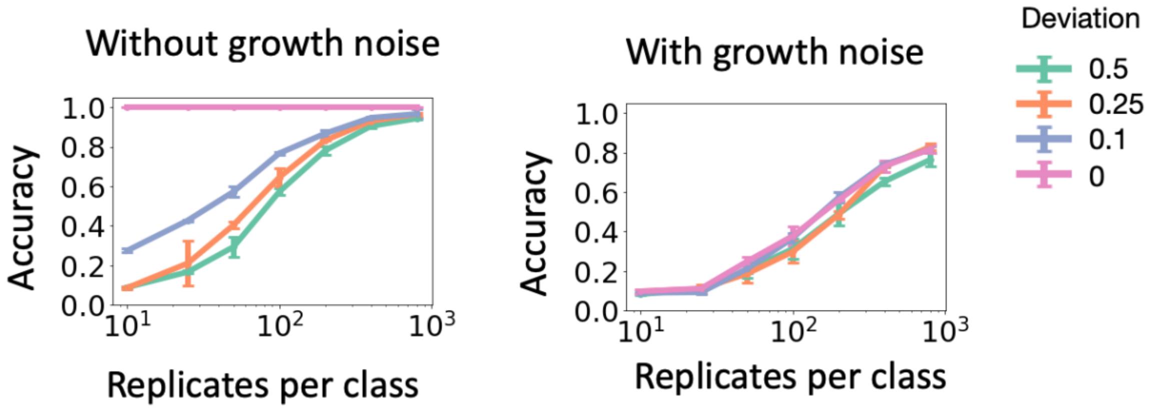

Fig. S6. Impact of seeding noise on the tradeoff among encoding capacity, security, and decoding reliability.

The seeding noise is implemented by assigning each pixel within the seeding configuration a random value. These values are drawn from a truncated Gaussian distribution with a mean of 0.5 and a given deviation (0, 0.1, 0.25 and 0.5 respectively). Larger deviation results in larger seeding noise. In the absence of growth noise, larger seeding noise leads to more challenging decoding, which is indicated by the increasing required training data. However, this impact is minor and can be obscured by a gentle growth noise (SNR = 10, see “Methods” for more details). Data are represented as mean ± standard deviation.

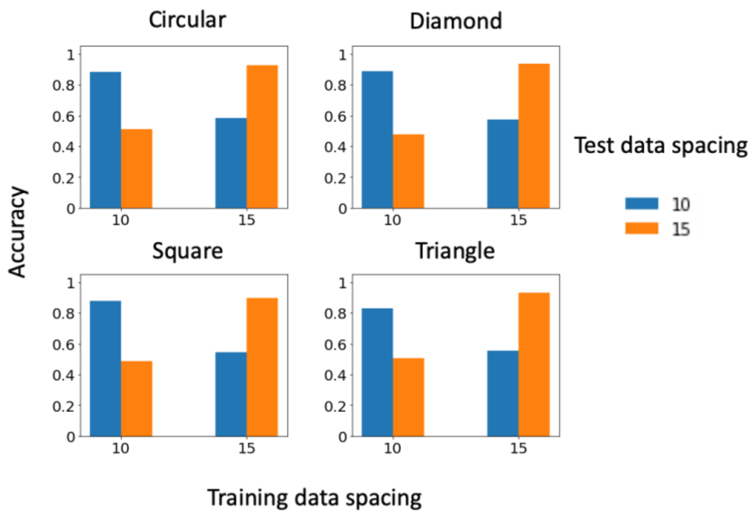

Fig. S7. Spacing as the encryption secret key.
Spacing distance = 10 and 15 were used respectively as the secret key to generate the training patterns. On growing media of all shapes, only models trained with the correct dataset can decode the patterns at significantly higher accuracy. The results indicate that spacing is a feasible choice for the secret key.

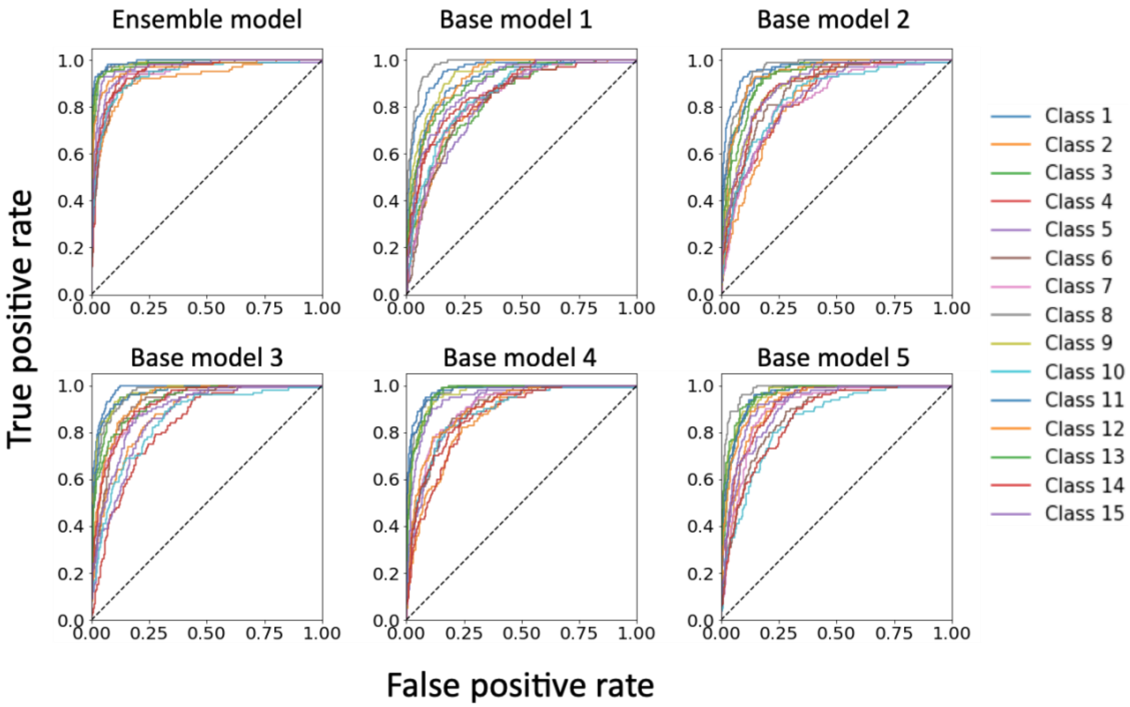

Fig. S8. Multiclass ROC of ensemble and base models.
We used 5 base CNN models to train a LR ensemble model. The training data were patterns generated using moderate growth noise (SNR = 3.5) and the dataset was composed of 100 replicates per class. Among base models, the ROC curves vary drastically. On the contrary, the variation reduces for the ensemble model, and the curves shift towards the upper left corner indicating significant performance improvement.

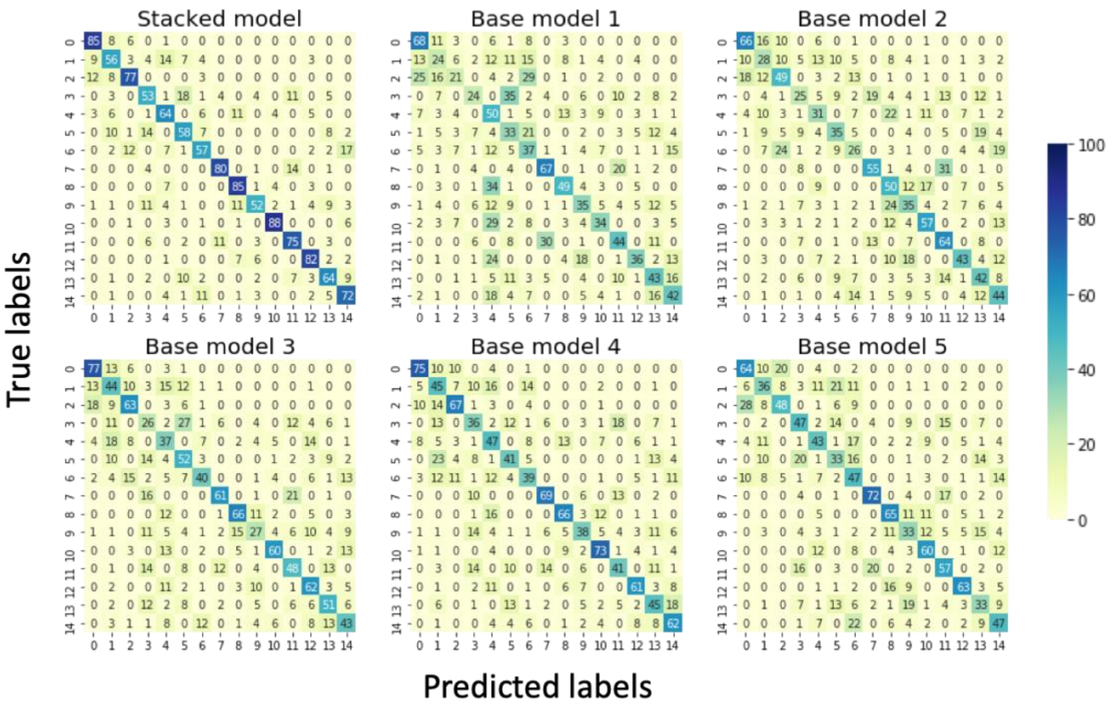

Fig. S9. Confusion matrix of ensemble and base models.

The base and ensemble models were trained on the same dataset in the same way as in Figure S6. Overall, the chance of misclassification reduces considerably for the ensemble model in comparison to individual base models.

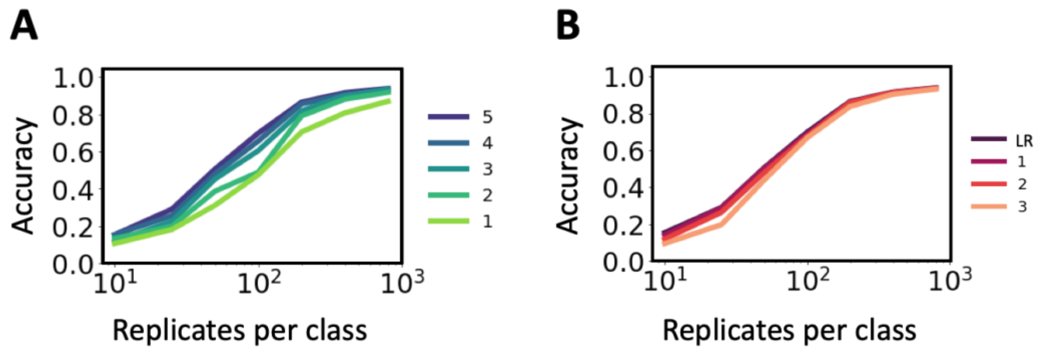

Fig. S10. The impact of the number of base models and ensemble model architecture on ensemble prediction.

A. Colors indicate different number of base models. The ensemble accuracy improves as more base models are used for constructing a LR ensemble model, and the amount of increase depends on the dataset size. The accuracy would eventually saturate when sufficiently many base models are used.

B. Logistic regression (LR) as the ensemble model outperforms feedforward neural network (FNN) with either 1, 2 or 3 hidden layers. Here, we kept the input and output layers the same for all models, and the number of hidden nodes for the FNNs are (40), (60, 30) and (60, 45, 25) respectively. LR performs slightly better or as good as FNNs.

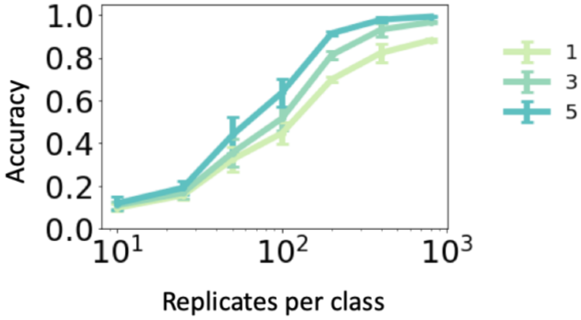

Fig. S11. Majority voting improves decoding accuracy.
Decoding accuracy of using 1, 3 and 5 patterns under the majority voting scheme. Using more patterns can significantly improve the performance, especially when a large dataset has been used for training. Data are represented as mean ± standard deviation.

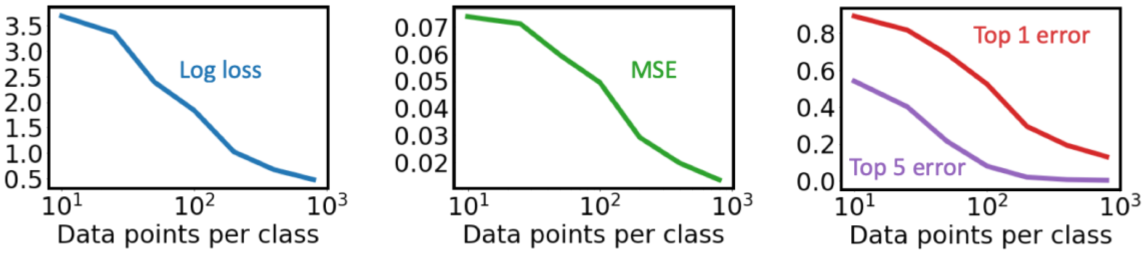

Fig. S12. Uncertainty estimation using deep ensemble.
5 base models trained using random initialization were used to calculate the selected metrics. Higher value indicates greater uncertainty. Having more training data lowers the prediction uncertainty.

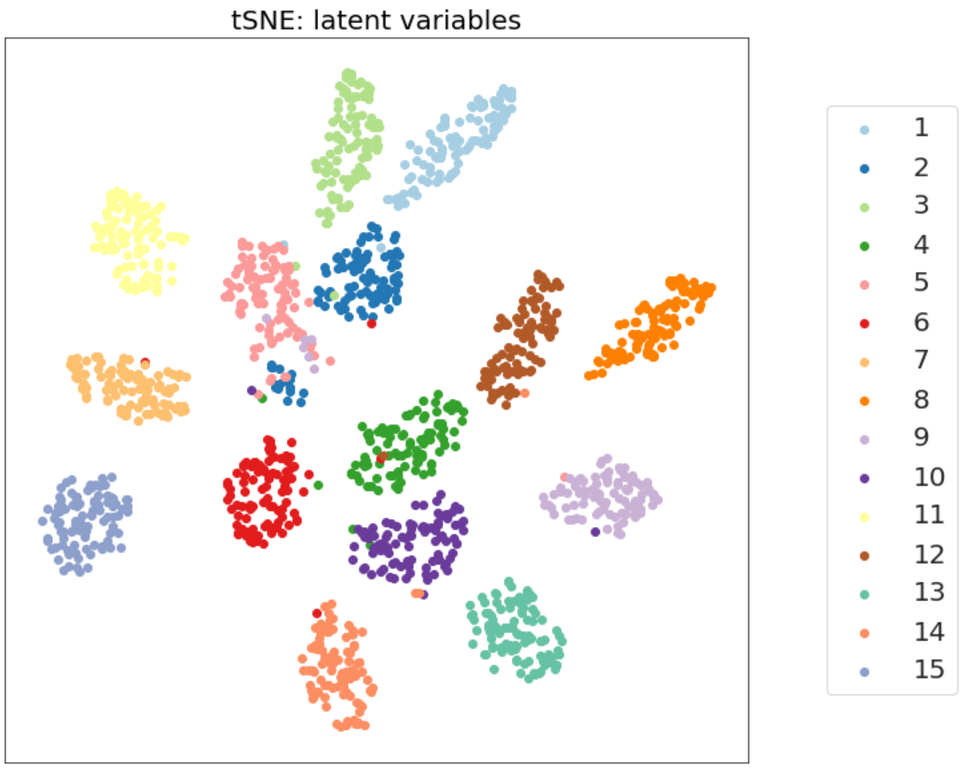

Fig. S13. t-distributed Stochastic Neighborhood Embedding (t-SNE) of data distribution. t-SNE was trained to embed CNN outputs (before fed to softmax) into 2D space using random initialization, perplexity 50, and learning rate 200 (implemented in scikit-learn). The training dataset consists of 15 initial configurations and 12000 data points, the simulation parameters are the default. The figure illustrates the distribution of 100 randomly selected patterns from each initial configuration (each labeled by a color). The results show that the patterns encoding different characters are indeed distinguishable as t-SNE learnt to cluster them.

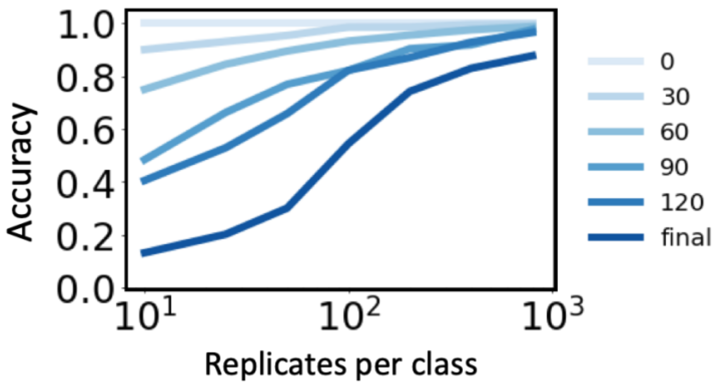

Fig. S14. Temporal information encoding using branching patterns.
Decoding accuracy on patterns arrested from growth at t = 0, 30, 60, 90, 120 and when the colony stops expanding (“final”). The patterns stopped growing at earlier time points, require less training data to achieve the same decoding accuracy.

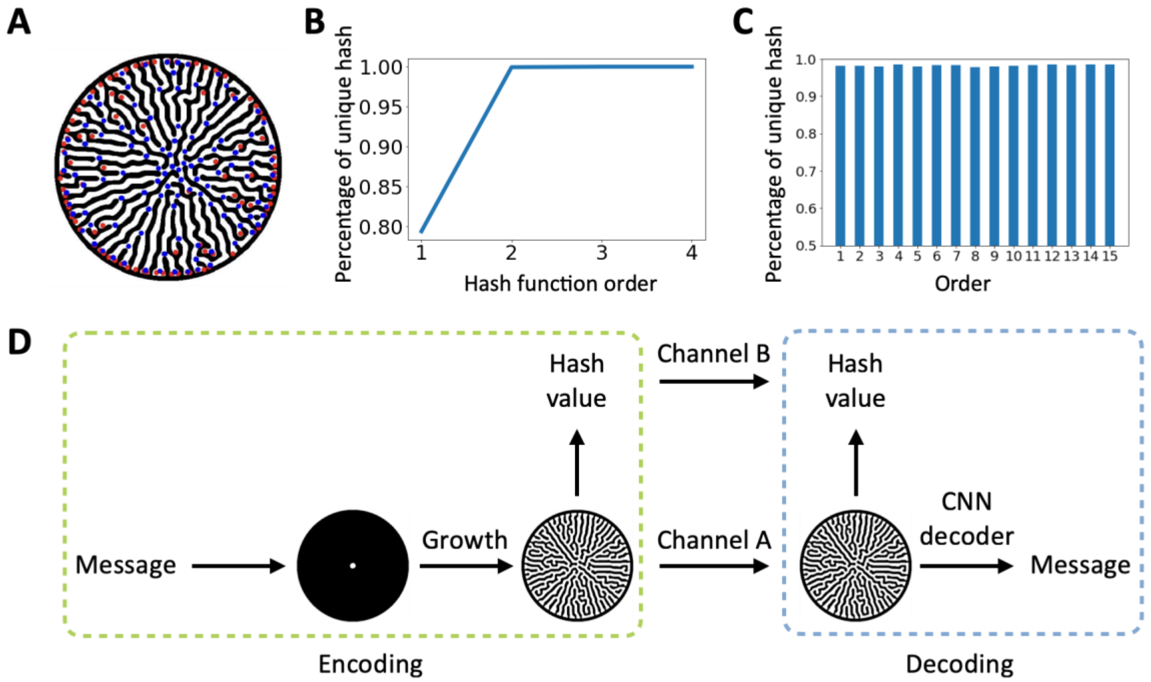

Fig. S15. Protecting information integrity using biological noise

A. Extracted minutiae (blue: bifurcation, red: branch ridge) of an example pattern.

B. The percentage of unique hashes. The hashes were computed using symmetric hash functions with order k = 1, 2, 3, and 4, respectively on a dataset of 15000 patterns (15 classes, 1000 replicates of each class). The default parameter set was used for data generation. To compute the percentage, we removed 217 duplicate patterns and normalized the counts with respect to the total number of unique patterns (14783). The figure indicates that increasing the order of hash function can reduce the chance of two different patterns sharing the same hash.

C. The percentage of unique hashes by class. The hash function here has k = 1. The calculation was carried out on the same dataset as in panel B. The percentage of unique hash was normalized with respect to the number of unique patterns for a given class. The results show that the chance of collision is comparable among all classes.

D. Integrity check procedure. In real communication, the sender would send a pattern and its hash through different channels (channel A and B, respectively) to the recipient. Once the recipient receives both, he/she would first authenticate information integrity by computing the hash of the received pattern and comparing it with the hash received through channel B. If they agree, the recipient can proceed to decode. Otherwise, reject the pattern and request a new one. This procedure is analogous to a typical two factor authentication.

Table S1. Converting encoding characters into binary representations.
For an example dictionary of 15 characters (A-E, 0-9), one possible way of encoding is to first convert them into 4-bit binary numbers in order. Binary number "0000" is purposefully avoided, because 0 corresponds to the absence of cells in the seeding configuration, thus colony patterns cannot form.

Encoding character	Binary representation	Encode character	Binary representation
A	0001	3	1001
B	0010	4	1010
C	0011	5	1011
D	0100	6	1100
E	0101	7	1101
0	0110	8	1110
1	0111	9	1111
2	1000		

Table S2. Estimated uncertainty as a function of training data size and the number of base models. We trained LR ensemble model using differently sized dataset and number of base models. For the same dataset, the estimated uncertainty does not change drastically with respect to the number of base models.

Replicates per class	Number of base models	LL	MSE	Top 1 error	Top 5 error
10	5	3.687	0.074	0.107	0.541
	4	3.675	0.074	0.108	0.537
	3	3.645	0.073	0.098	0.552
	2	3.545	0.072	0.108	0.533
100	5	1.839	0.049	0.476	0.080
	4	1.854	0.050	0.470	0.088
	3	1.903	0.051	0.448	0.097
	2	2.010	0.054	0.419	0.110
800	5	0.478	0.014	0.869	0.004
	4	0.423	0.012	0.881	0.003
	3	0.425	0.012	0.882	0.003
	2	0.446	0.012	0.884	0.002

Supplemental Experimental Procedures

Temporal information encoding and decoding

In dynamical systems, information embedded in the initial conditions may dissipate over time(1). Thus, it is necessary to determine the temporal encoding-decoding performance of our patterning system. We simulated branching patterns using the same protocol as described in the Methods. Instead of waiting until the colony stops growing, we arrested the simulation at different computational time points (Figure S1). We observed a similar trade-off among capacity, security and decoding reliability as mentioned in the main text (Figure S12). The patterns arrested at earlier time points have higher encoding capacity and lower security, as they require fewer training data to achieve the same accuracy compared to the later patterns. This is possibly because as the patterning process goes on, the colony starts developing branches that obscure the initial configuration information and shrinks the inter-categorical similarity. The accuracy curves are bounded from below by the curve of the final patterns, implying that the final patterns provide the highest security among all. Moreover, the results indicate that temporal regulation of pattern formation would be an additional strategy to modulate encoding capacity and security.

Authenticating patterns using noise signatures

Information integrity is critical for reliable communication and could be compromised under different scenarios. For instance, the attackers could alter the patterns to prevent delivering important messages or replace them with fake ones to deceive the intended recipient (ex. phishing). Therefore, it is critical to carry out an integrity check before decoding. One plausible method is through hashing, where a unique hash is generated and assigned to a message. When the pattern is tampered, the hash should change drastically (so-called *avalanche effect*) and fail to match the original one, indicating potential attacks and damage. Moreover, the hash function should be designed so that the chance of two different patterns having the same hash value (i.e., collision) is low.

In our system, branching dynamic amplifies the inherent biological noise in cell seeding and growth, resulting in patterns that are similar globally but vary in detail. We leveraged this feature and implemented hashing in the pattern-based communication platform. For a given pattern, we first locate its bifurcation and branch ridges (Figure S13A). To compute a unique hash, we input the extracted minutiae locations into a symmetric hash function(2). This class of hash functions has the advantage of being order-invariant, such that it allows us to bypass the challenge of assigning orders to minutiae. Consider an extracted pattern minutia has location $(x_i, y_i),\ i \in \{1,2,\dots,N\}$, where N is the total number of minutiae. We can design hash function $h_k = \sum_{i=1}^{N}(x_i^k + y_i^k)$, where k indicates the order of the function. As a proof of principle, we tested hash functions of different orders on an example dataset and found that it was possible to find order k that minimizes the chance of collision for patterns encoding different characters (Figure S13B) and patterns encoding the same characters (Figure S13C). Moreover, it is also possible and probably more robust to match the patterns by comparing multiple hash values all at once.

In practice, the end-users can use the hash values to carry out pattern integrity checks before preceding to decode, similar to two-factor authentication (Figure S13D). Here, we focused only on the malicious tampering of patterns other than transformation or numerical errors caused by minor transmission distortion. Considering the similarity between matching branching patterns and human fingerprints, further studies could design and apply more robust hash functions, such as those used on biometric data(3-5).

Encoding and decoding using elementary cellular automaton

The elementary cellular automaton (ECA) model is one-dimensional, each cell has a state of either 0 or 1. The system starts from an initial sequence of cells, defined in a similar manner as

in the Deng model (Figure S4C). Each character is first converted into a binary number, which is then translated into an initial configuration according to a predefined array. The cell status at the next time step depends on the current status of its neighbors. We implemented rule 60 that is weakly chaotic. Here 60 represents binary number 00111100, each digit represents the resulting cell status in the corresponding scenario (Figure S4A). For example, in the first scenario (Figure S4A, the most left), the cell of interest (middle) and its two neighbors have status of 111 at the current time step, the first 0 in the binary number means the cell of interest will have status 0 at the next time step.

Unless specified, the default encoding parameters are: sequence length = 450, time step = 500, spot radius = 10, dictionary size = 15, spacing = 70. Noise is imposed onto the seeding sequence before evolution starts. We draw random numbers from a uniform distribution and assign them to each cell within the initial configuration. We define a threshold ρ, cells with value smaller than ρ will flip from status 1 to 0, otherwise they will keep status 1. In other words, ρ is the percentage of cells who flip their status, larger ρ indicates larger noise level. We use default ρ of 0.5. These parameters were chosen such that the system gives satisfying security level. To decode, we trained a feedforward neural network to classify the output sequences via classification. The mathematical modeling was implemented in MATLAB R2020b, the ML model training implemented in Python3 and PyTorch 1.9.

Figure S5 shows that increasing noise level and small spacing deteriorate decoder performance, which is consistent with the observations with colony patterns. The decoding accuracy can be partially rescued by increasing the training data size. Dictionary size does not impact the performance, indicating each system have different encoding capacity and must be tuned individually.

Supplementary References

1. Gade P, Amritkar R. Characterizing loss of memory in a dynamical system. Physical review letters. 1990;65(4):389.
2. Tulyakov S, Farooq F, Govindaraju V, editors. Symmetric hash functions for fingerprint minutiae. International Conference on Pattern Recognition and Image Analysis; 2005: Springer.
3. Das P, Karthik K, Garai BC. A robust alignment-free fingerprint hashing algorithm based on minimum distance graphs. Pattern Recognition. 2012;45(9):3373-88.
4. Lai Y-L, Jin Z, Teoh ABJ, Goi B-M, Yap W-S, Chai T-Y, et al. Cancellable iris template generation based on Indexing-First-One hashing. Pattern Recognition. 2017;64:105-17.
5. Kumar G, Tulyakov S, Govindaraju V, editors. Combination of symmetric hash functions for secure fingerprint matching. 2010 20th International Conference on Pattern Recognition; 2010: IEEE.

Appendix A

Encoding characters in Emorfi

To construct Emorfi, each of the printable ASCII characters (including English letters in upper and lower cases, digits, punctuations, and whitespaces) was converted into a binary representation, which was then converted into a unique initial configuration. For each configuration, we carried out mathematical simulation and obtained 1000 different patterns. These 100 sets of patterns, as well as subsequent ones to be generated, make up Emorfi. In the table below, three examples are shown for each character.

Encoding character	Binary representation	Initial configuration	Example patterns		
0	0000001	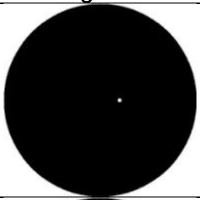	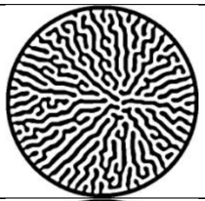	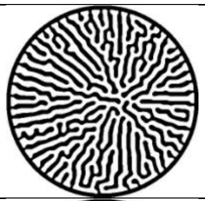	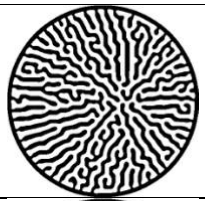
1	0000010	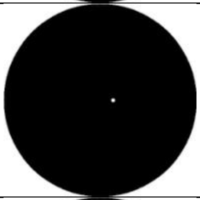	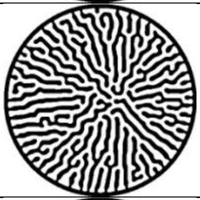	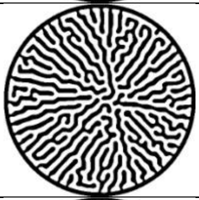	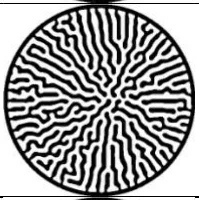
2	0000011	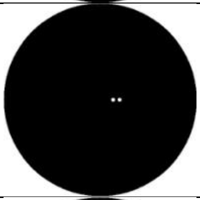	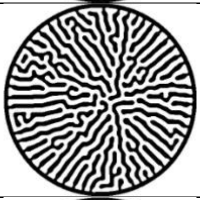	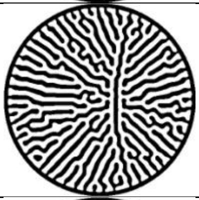	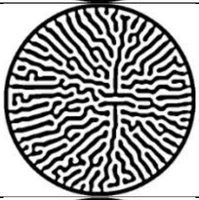
3	0000100	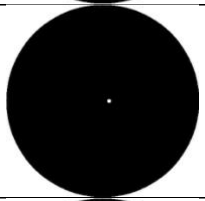	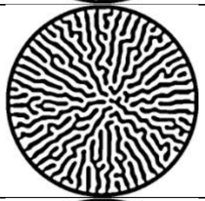	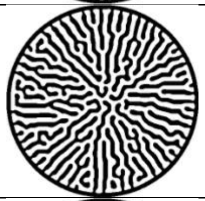	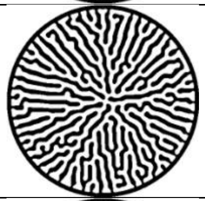
4	0000101	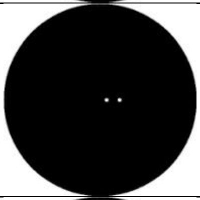	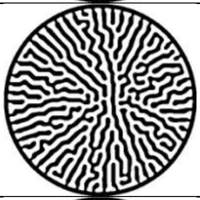	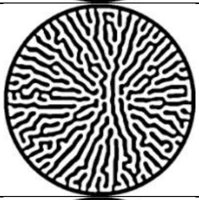	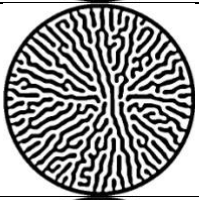
5	0000110	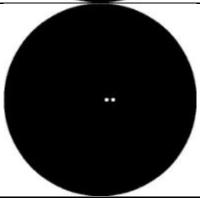	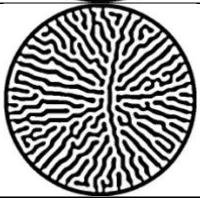	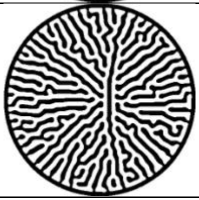	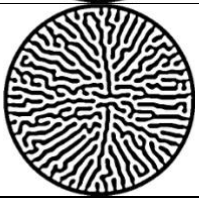

6	0000111	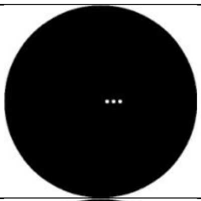	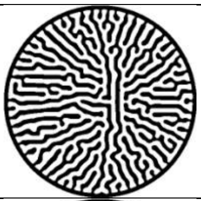	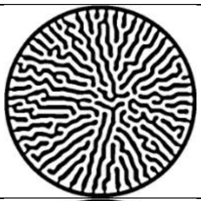	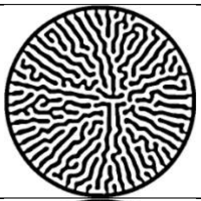
7	0001000	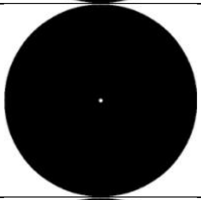	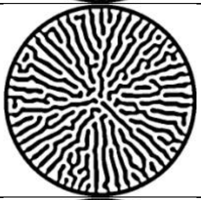	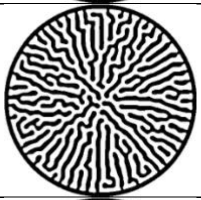	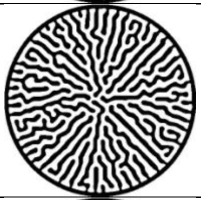
8	0001001	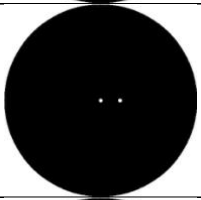	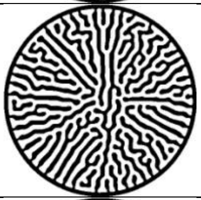	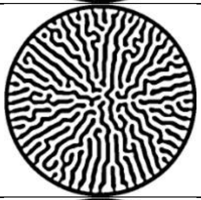	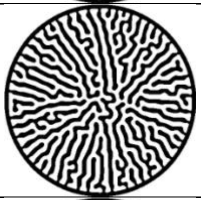
9	0001010	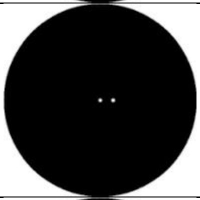	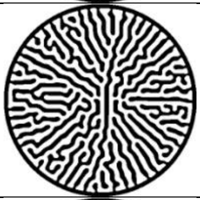	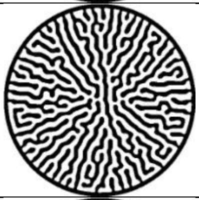	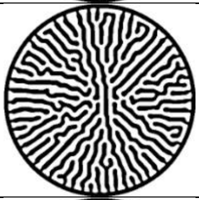
a	0001011	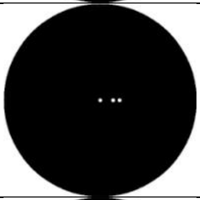	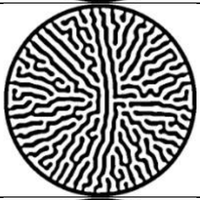	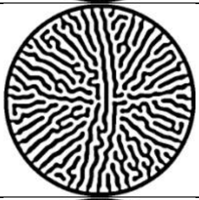	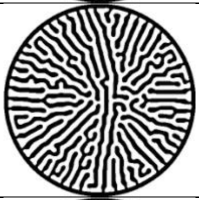
b	0001100	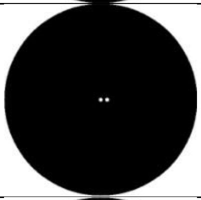	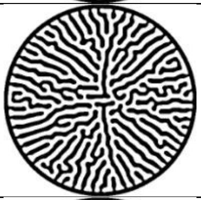	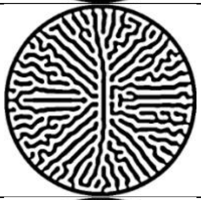	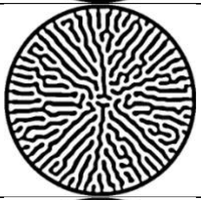
c	0001101	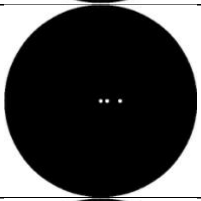	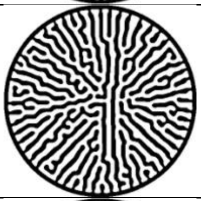	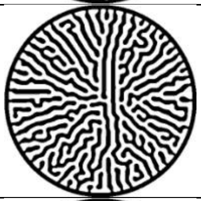	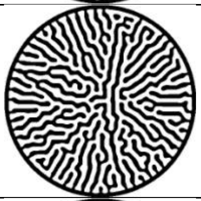
d	0001110	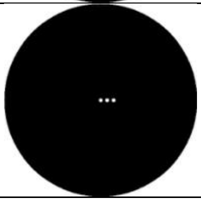	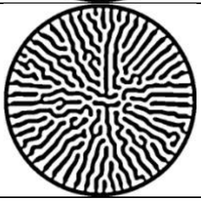	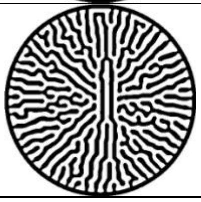	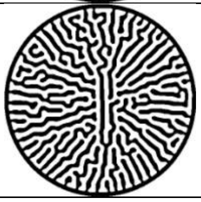

e	0001111	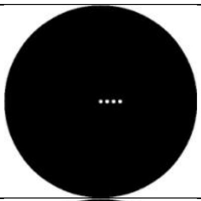	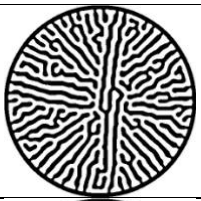	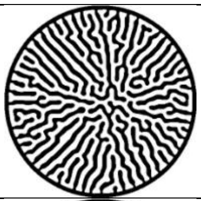	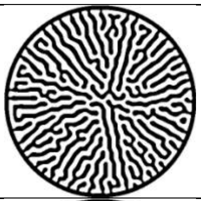
f	0010000	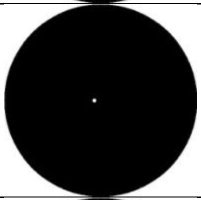	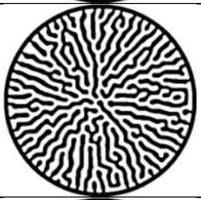	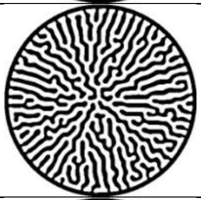	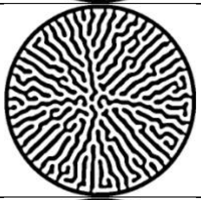
g	0010001	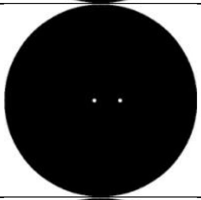	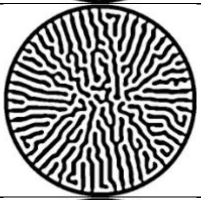	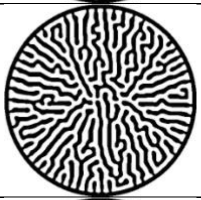	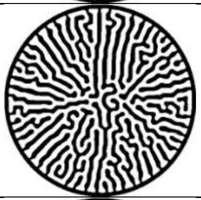
h	0010010	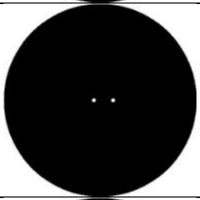	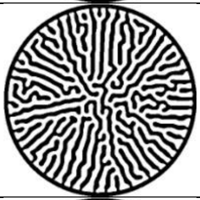	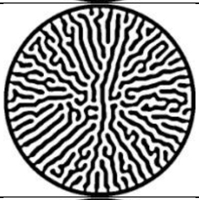	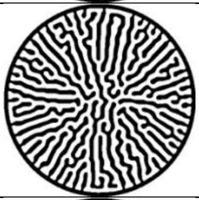
i	0010011	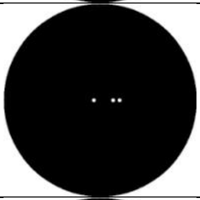	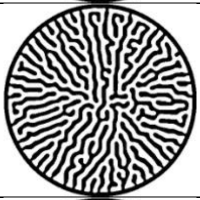	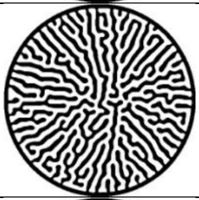	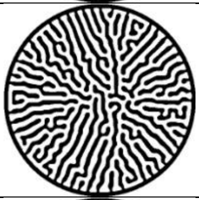
j	0010100	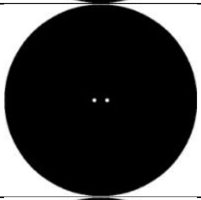	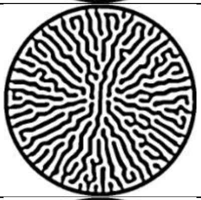	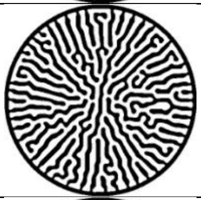	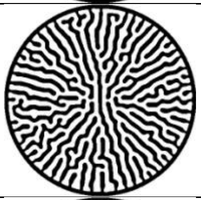
k	0010101	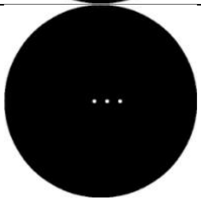	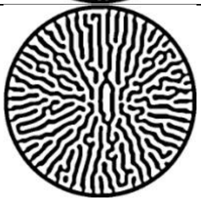	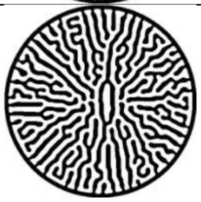	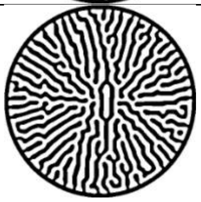
l	0010110	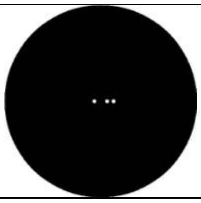	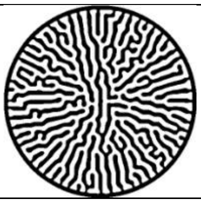	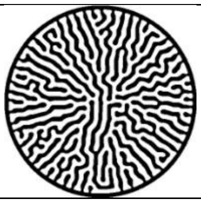	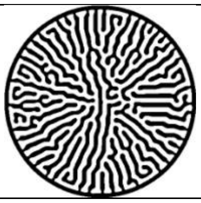

m	0010111	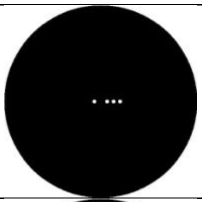	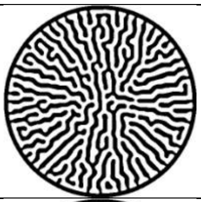	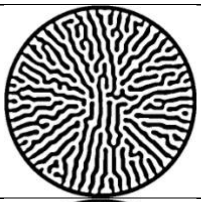	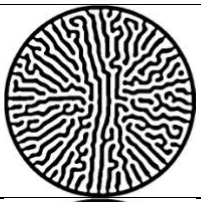
n	0011000	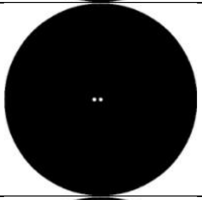	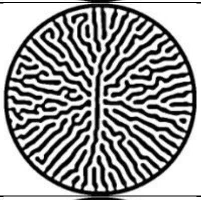	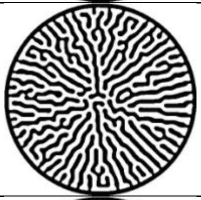	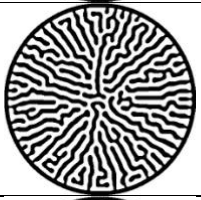
o	0011001	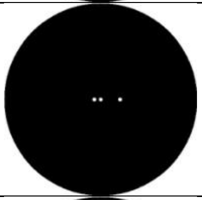	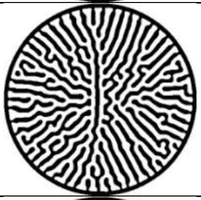	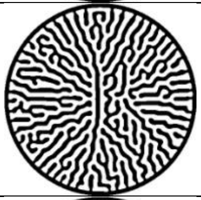	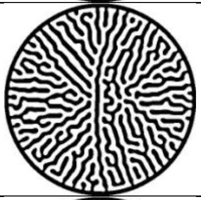
p	0011010	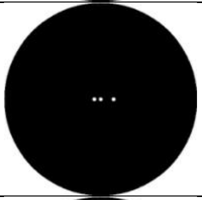	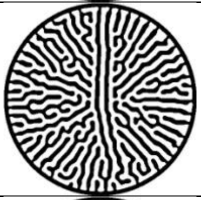	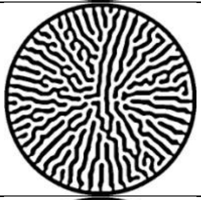	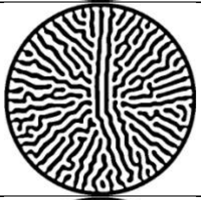
q	0011011	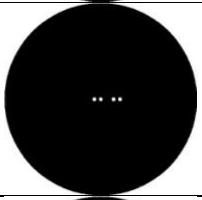	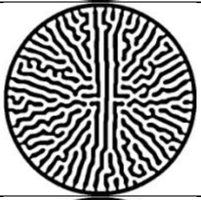	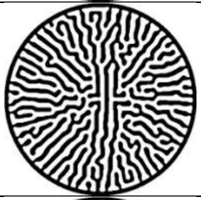	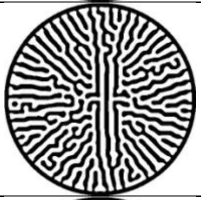
r	0011100	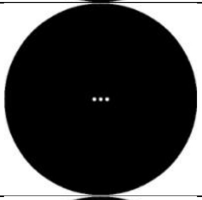	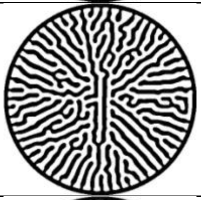	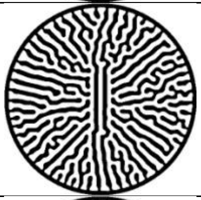	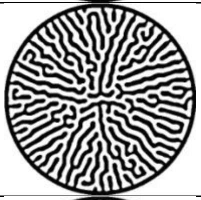
s	0011101	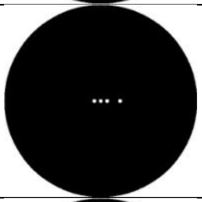	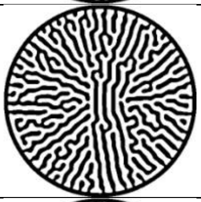	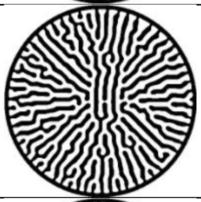	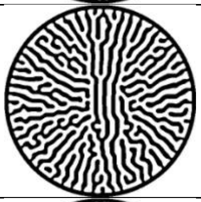
t	0011110	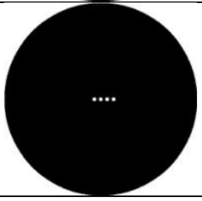	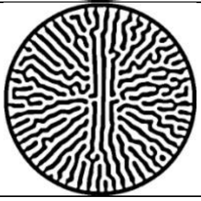	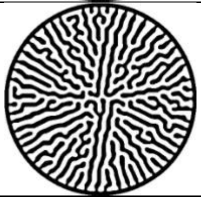	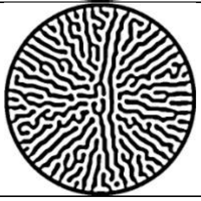

u	0011111	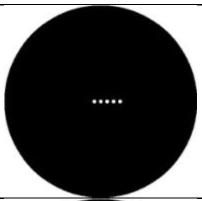	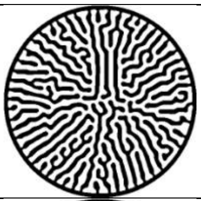	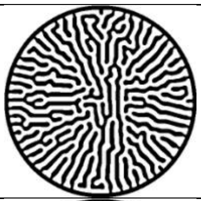	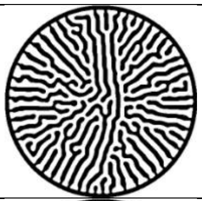
v	0100000	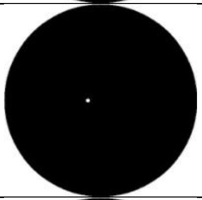	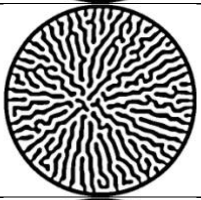	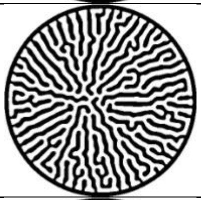	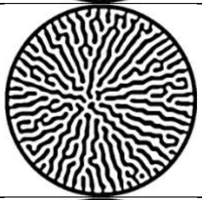
w	0100001	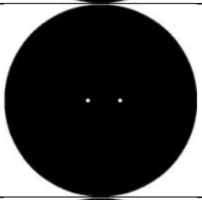	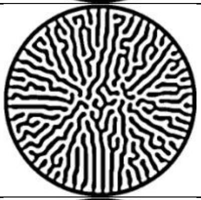	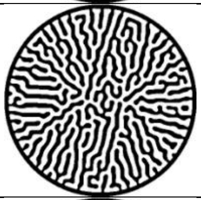	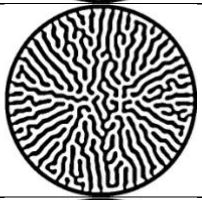
x	0100010	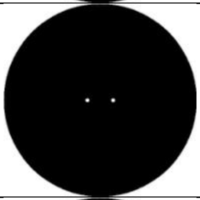	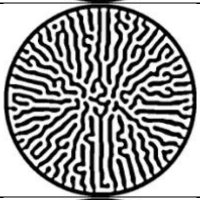	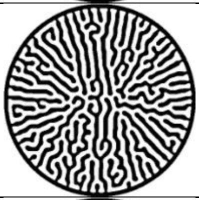	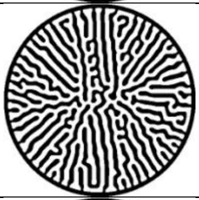
y	0100011	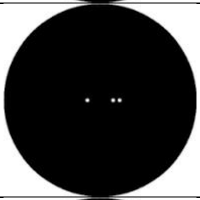	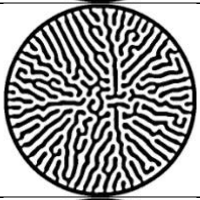	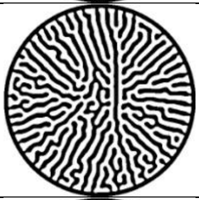	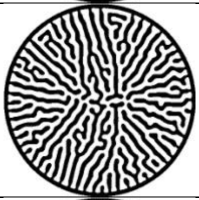
z	0100100	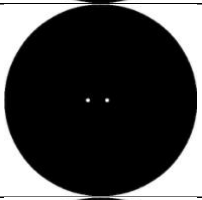	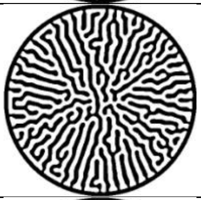	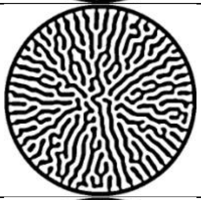	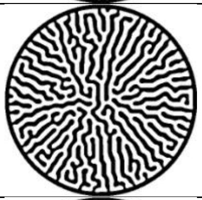
A	0100101	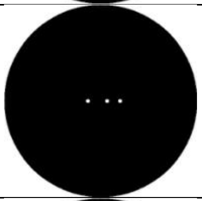	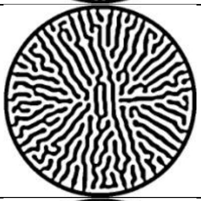	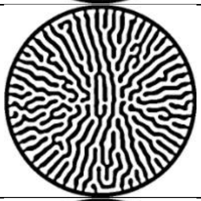	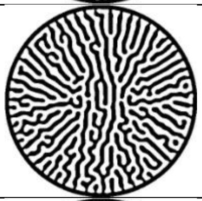
B	0100110	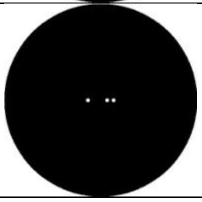	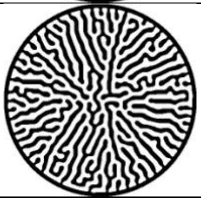	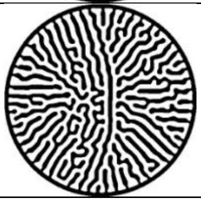	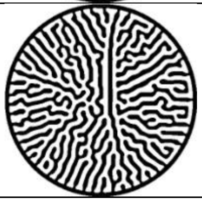

C	0100111	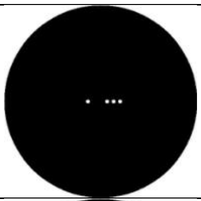	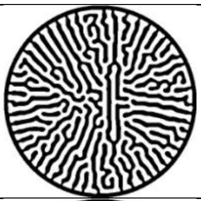	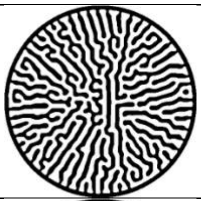	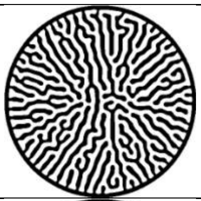
D	0101000	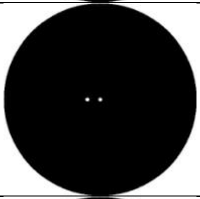	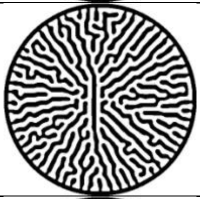	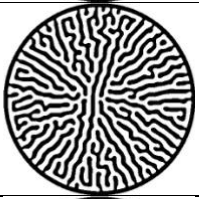	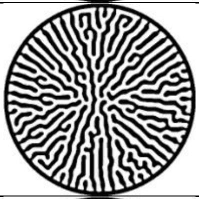
E	0101001	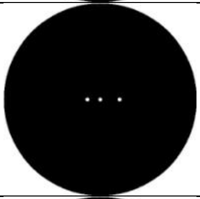	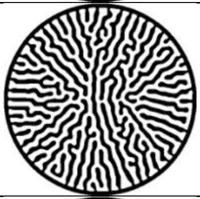	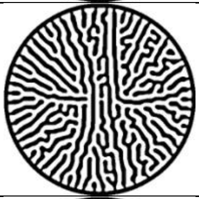	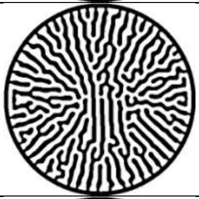
F	0101010	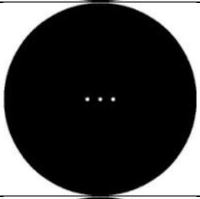	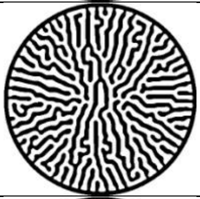	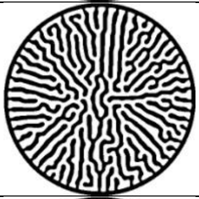	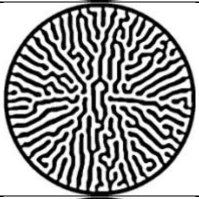
G	0101011	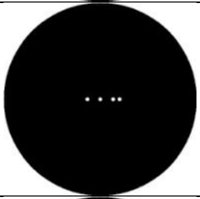	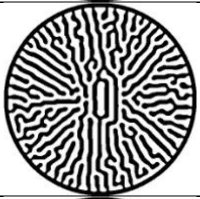	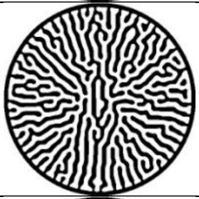	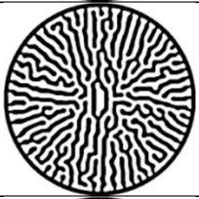
H	0101100	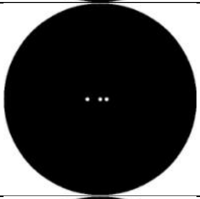	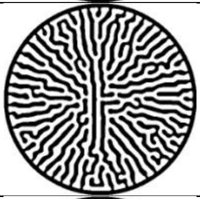	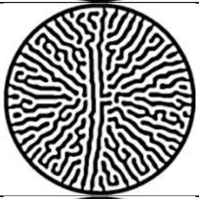	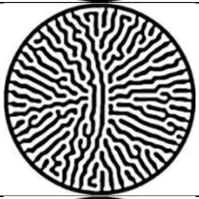
I	0101101	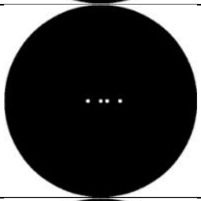	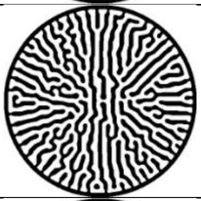	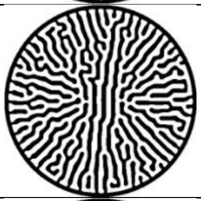	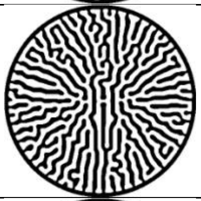
J	0101110	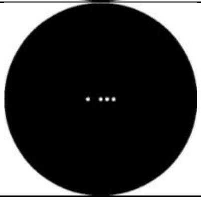	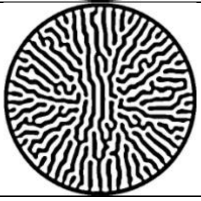	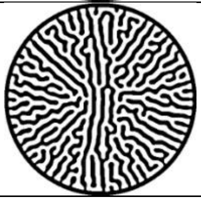	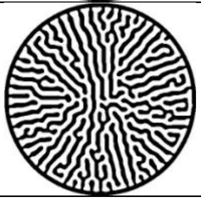

K	0101111	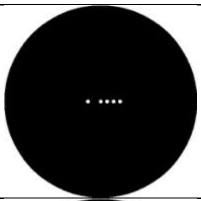	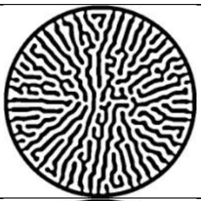	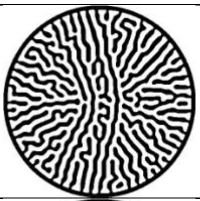	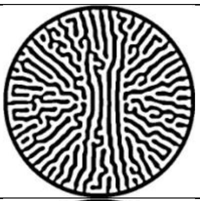
L	0110000	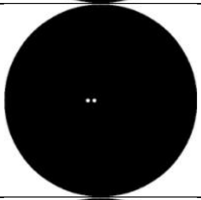	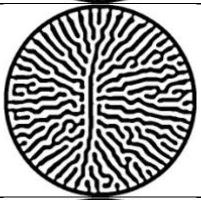	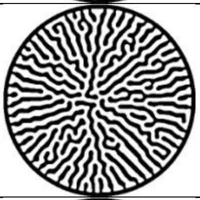	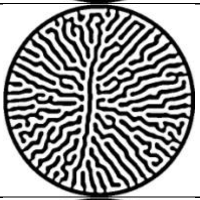
M	0110001	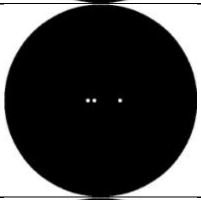	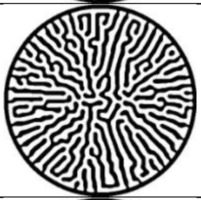	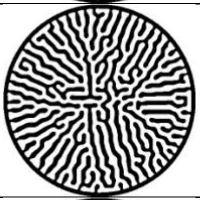	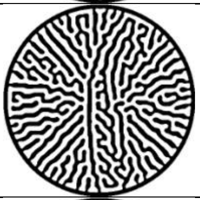
N	0110010	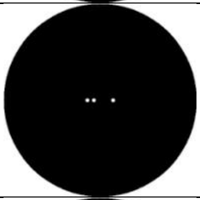	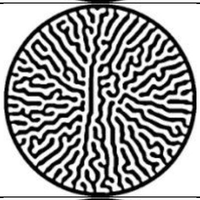	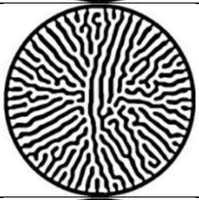	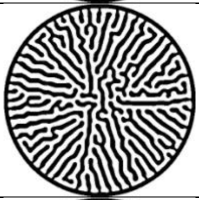
O	0110011	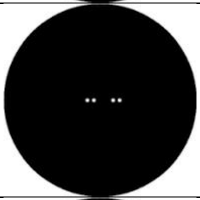	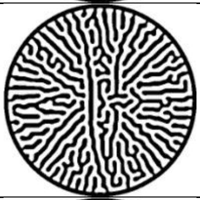	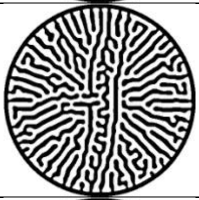	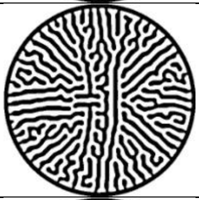
P	0110100	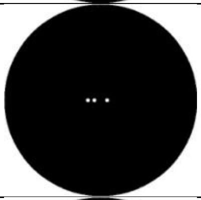	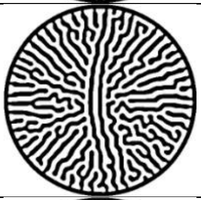	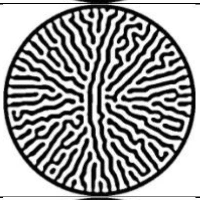	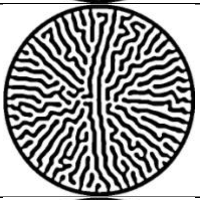
Q	0110101	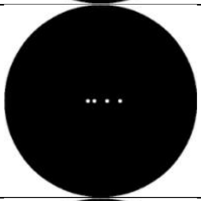	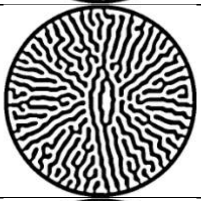	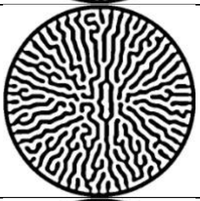	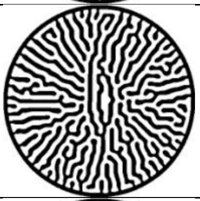
R	0110110	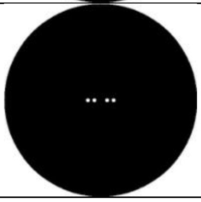	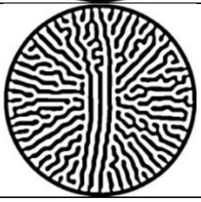	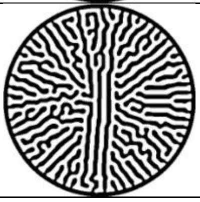	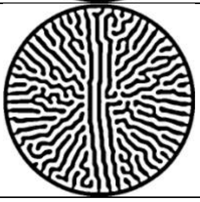

S	0110111	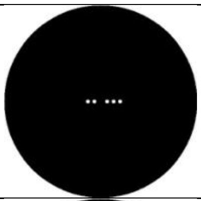	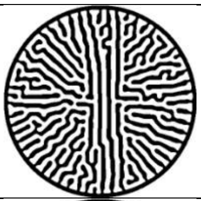	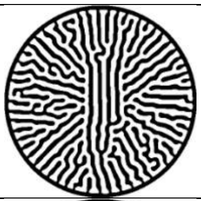	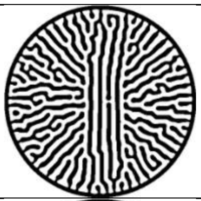
T	0111000	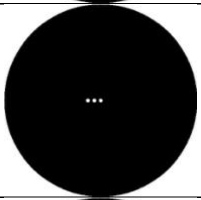	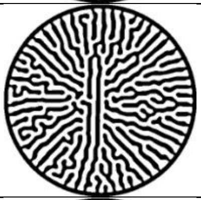	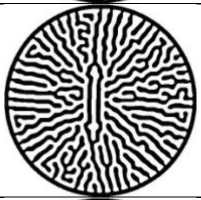	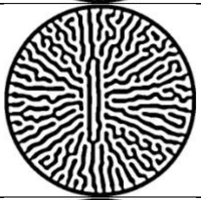
U	0111001	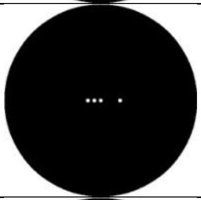	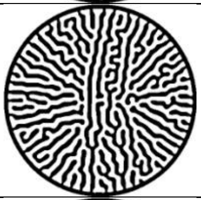	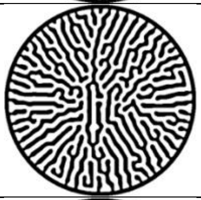	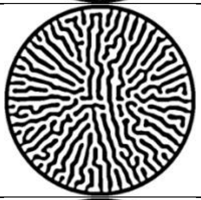
V	0111010	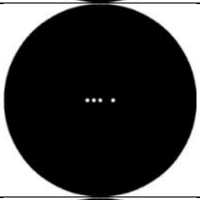	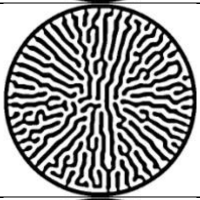	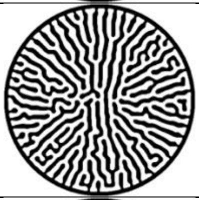	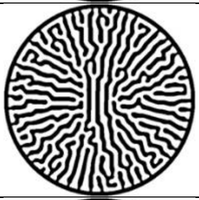
W	0111011	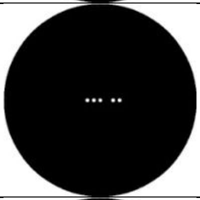	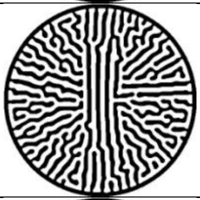	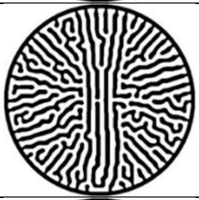	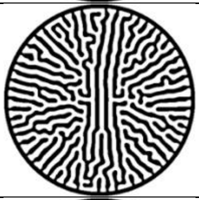
X	0111100	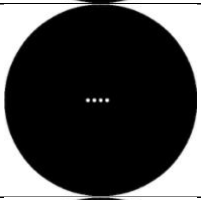	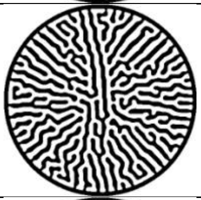	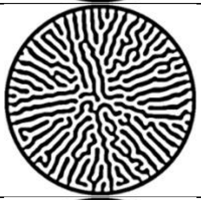	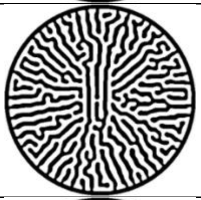
Y	0111101	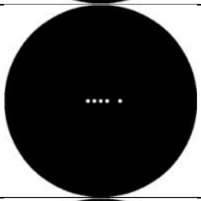	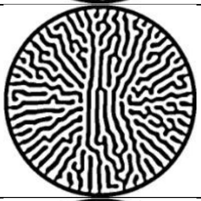	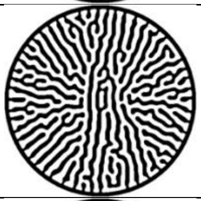	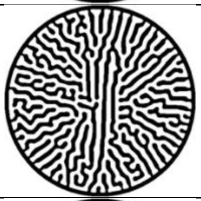
Z	0111110	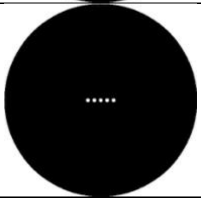	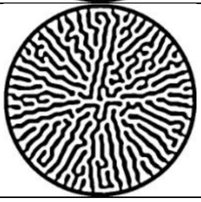	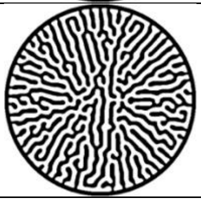	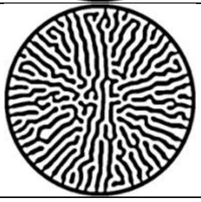

!	0111111	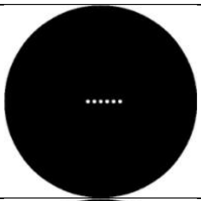	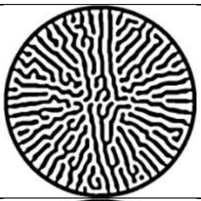	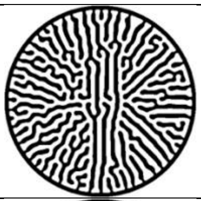	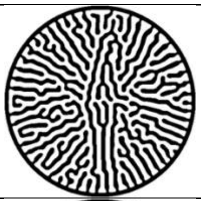
“	1000000	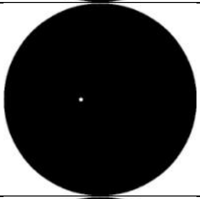	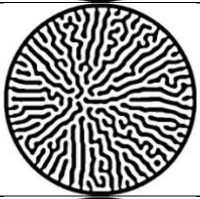	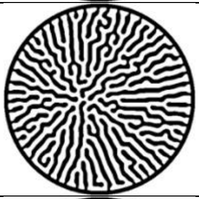	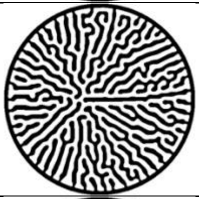
#	1000001	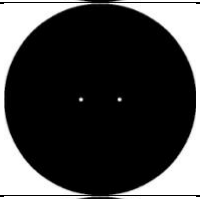	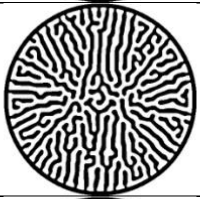	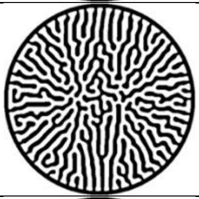	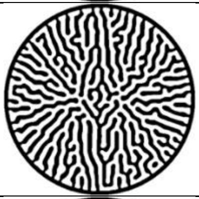
$	1000010	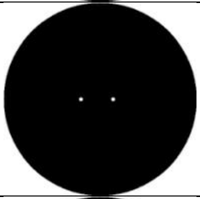	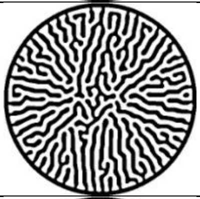	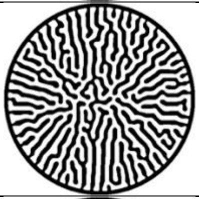	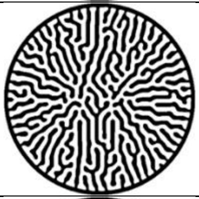
%	1000011	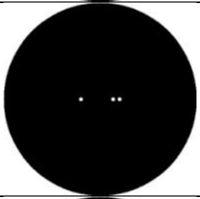	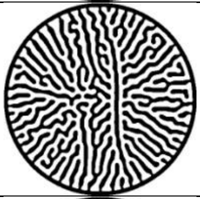	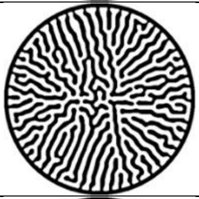	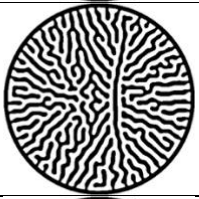
&	1000100	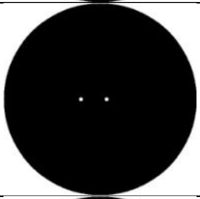	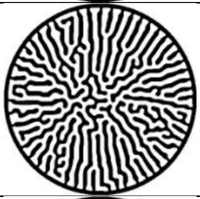	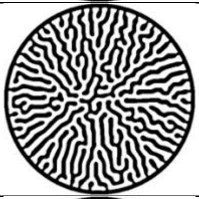	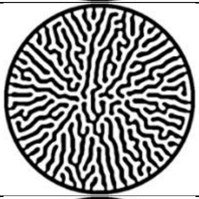
‘	1000101	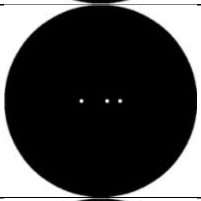	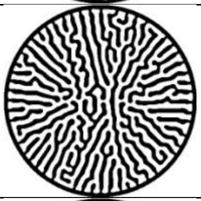	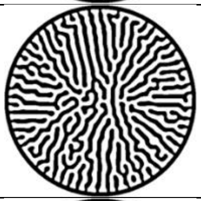	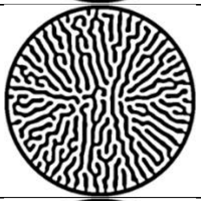
(	1000110	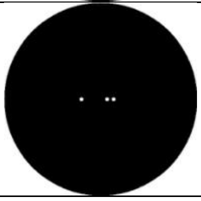	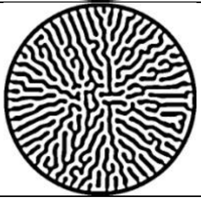	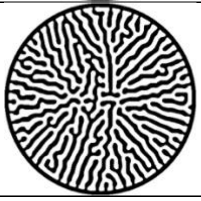	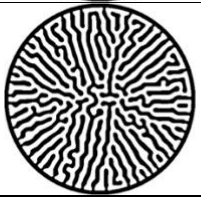

)	1000111	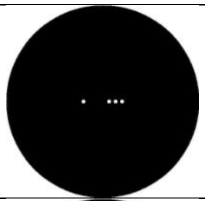	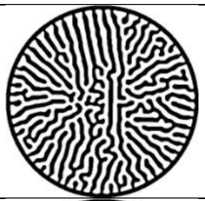	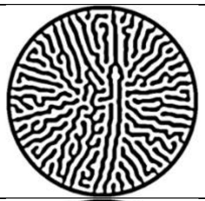	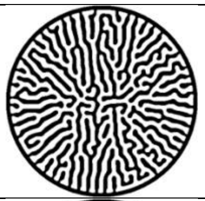
*	1001000	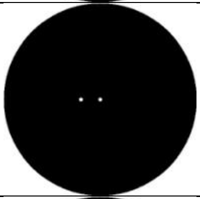	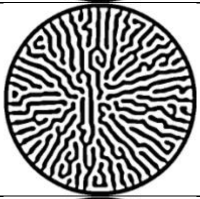	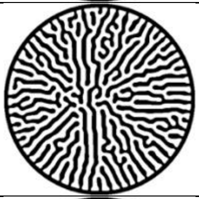	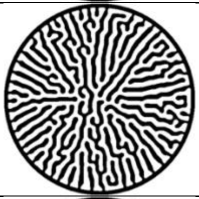
+	1001001	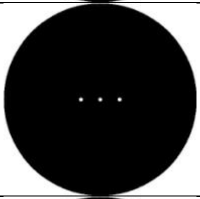	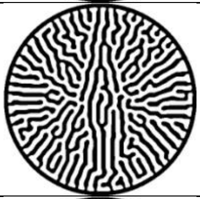	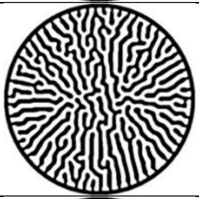	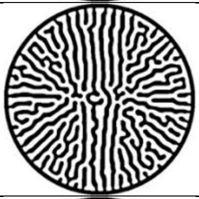
,	1001010	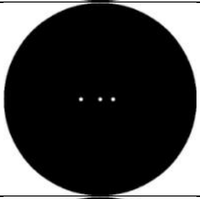	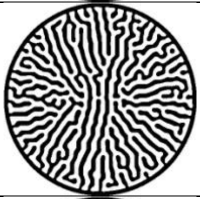	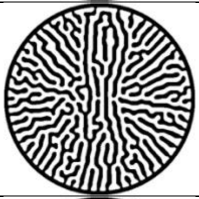	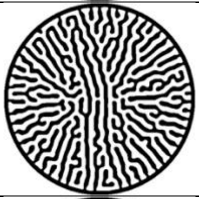
-	1001011	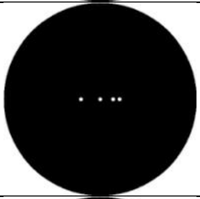	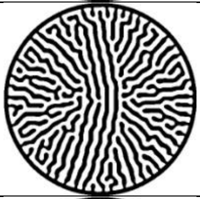	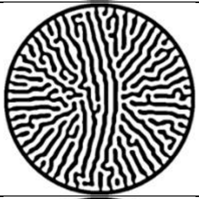	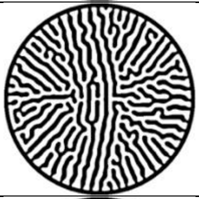
.	1001100	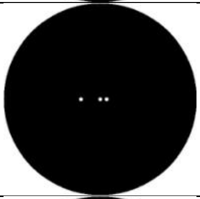	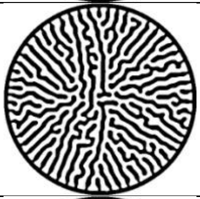	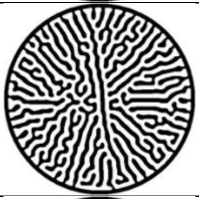	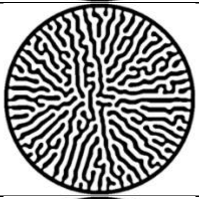
/	1001101	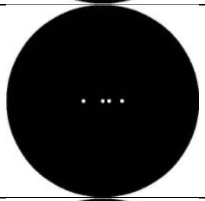	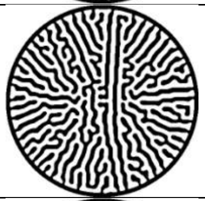	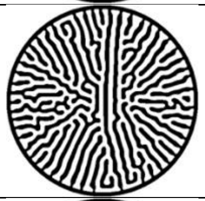	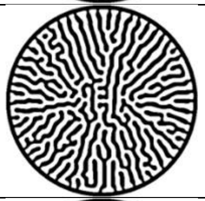
:	1001110	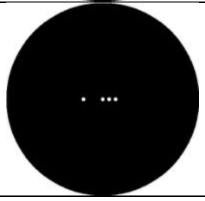	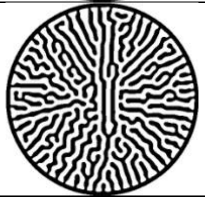	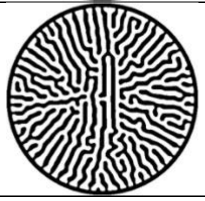	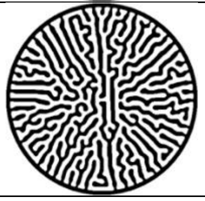

;	1001111	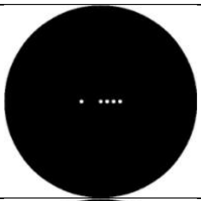	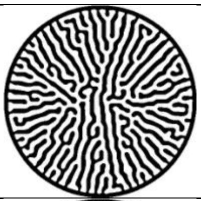	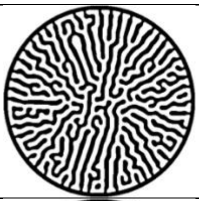	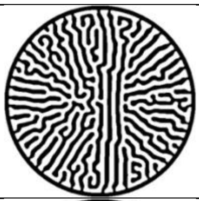
<	1010000	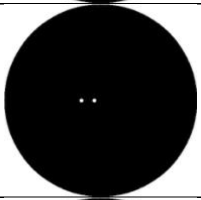	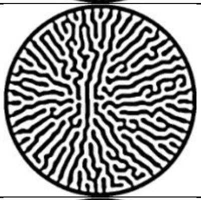	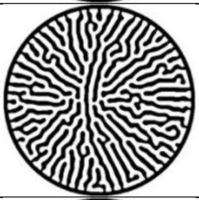	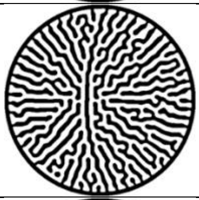
=	1010001	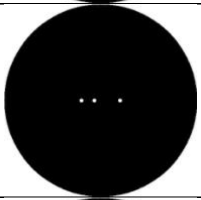	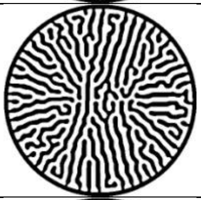	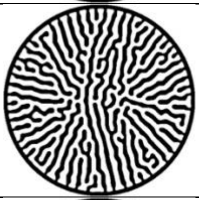	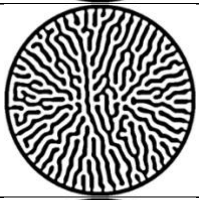
>	1010010	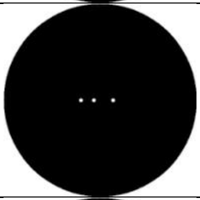	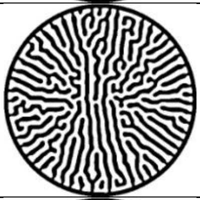	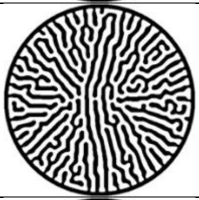	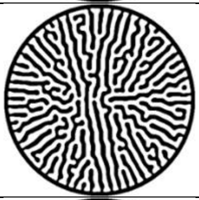
?	1010011	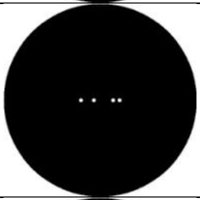	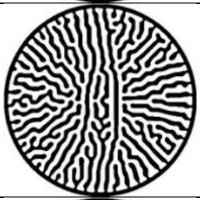	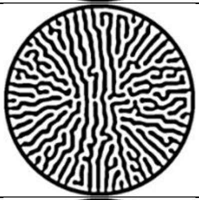	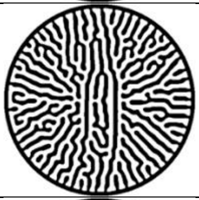
@	1010100	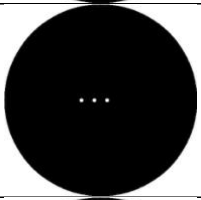	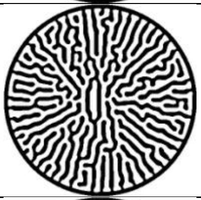	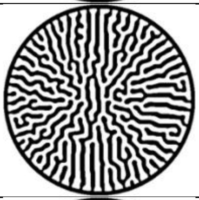	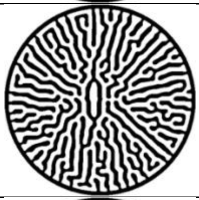
[	1010101	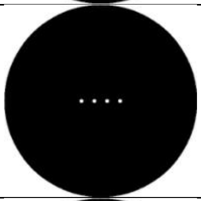	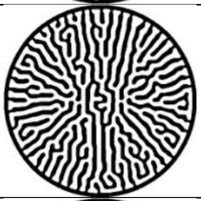	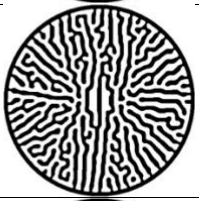	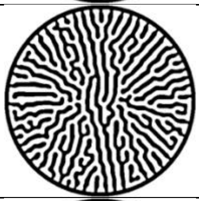
\\	1010110	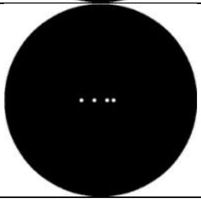	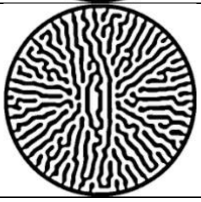	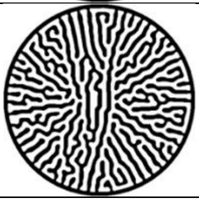	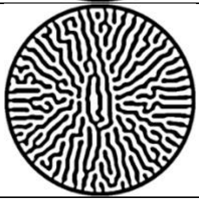

]	1010111	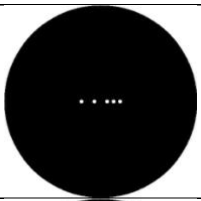	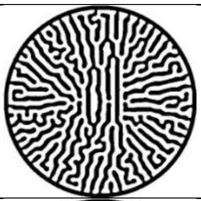	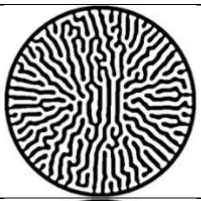	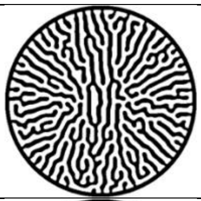
^	1011000	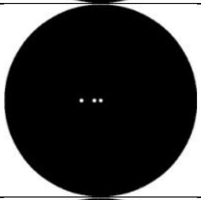	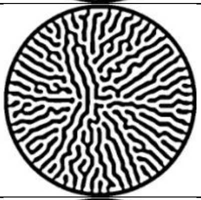	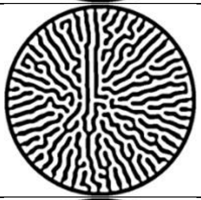	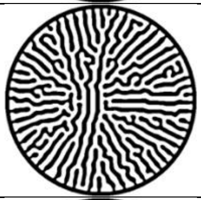
_	1011001	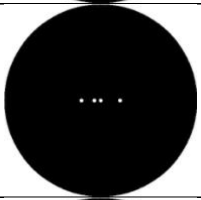	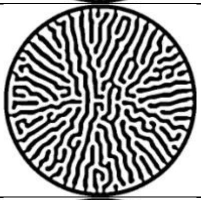	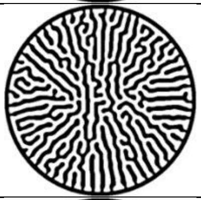	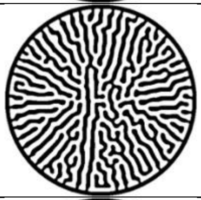
`	1011010	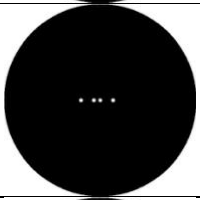	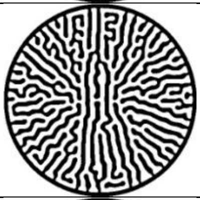	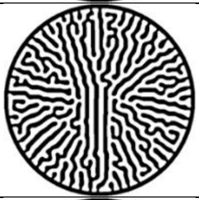	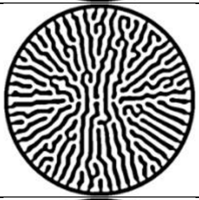
{	1011011	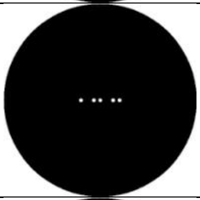	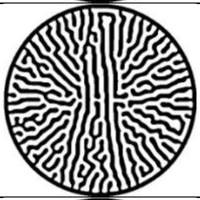	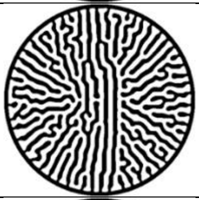	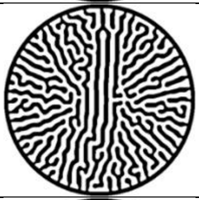
|	1011100	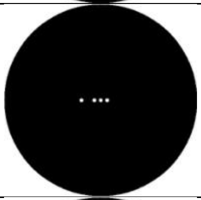	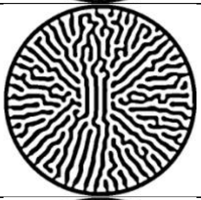	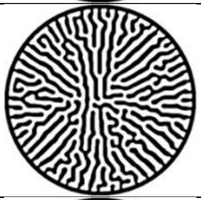	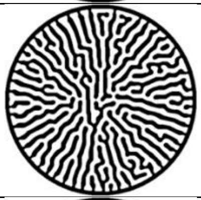
}	1011101	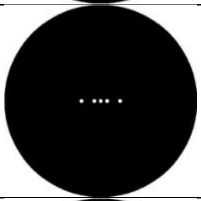	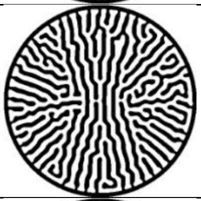	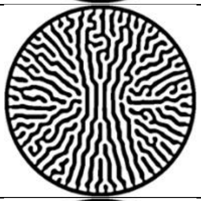	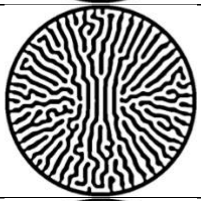
~	1011110	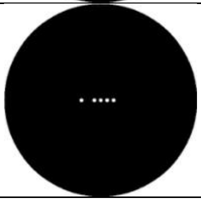	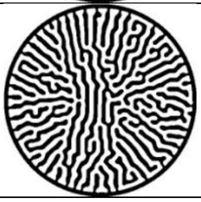	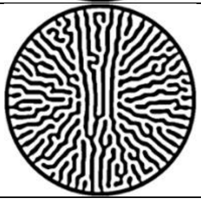	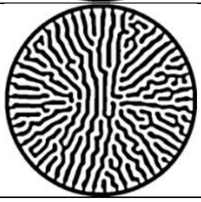

Character space	1011111	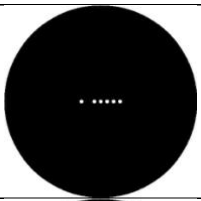	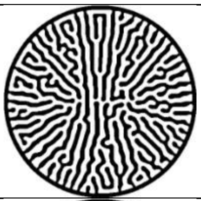	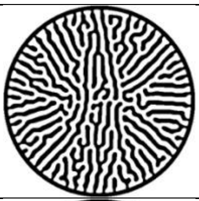	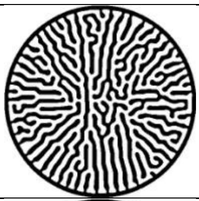
\t (tab)	1100000	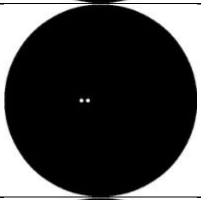	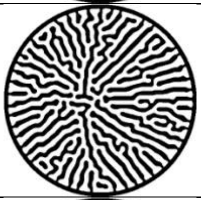	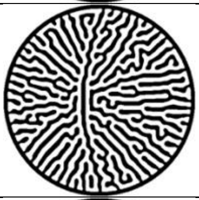	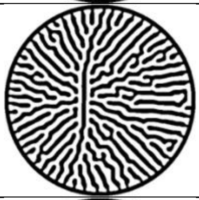
\n (linefeed)	1100001	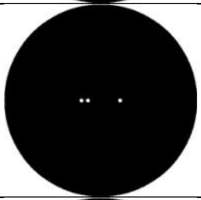	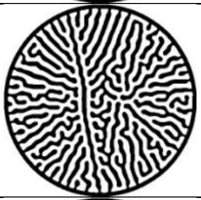	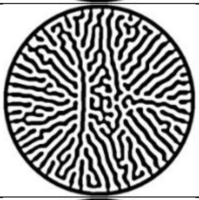	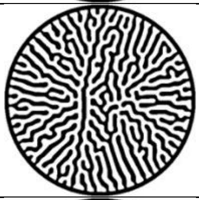
\r (return)	1100010	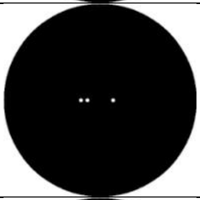	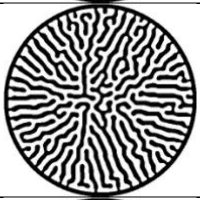	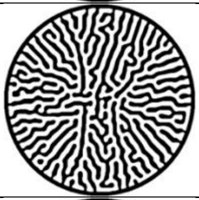	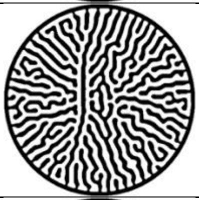
\x0b (vertical tab)	1100011	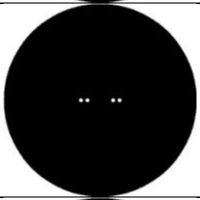	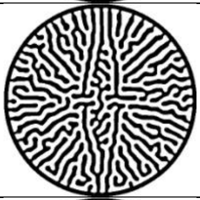	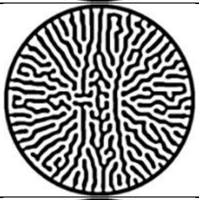	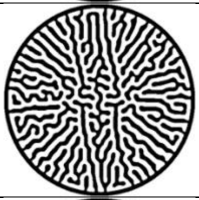
\x0c (formfeed)	1100100	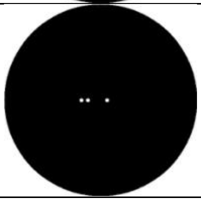	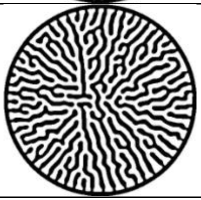	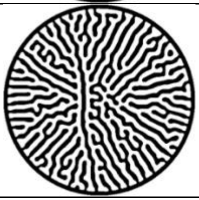	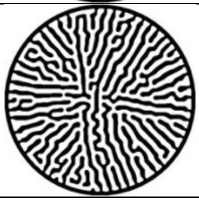

Appendix B

Encoding “I Have a Dream” in Emorfi and decoding it back in English

Each character was encoded in a randomly selected pattern from its corresponding class (see Appendix A). Next, the patterns were assembled in order into a video. To accommodate majority voting, the above process was repeated four times and the new video was concatenated to the end of the existing one. When decoding, we used a trained CNN combined with majority voting, 99.8% of the text was correctly decoded.

I Have a Dream

By Martin Luther King Jr.

August 28, 1963

Original:

Five score years ago, a great American, in whose symbolic shadow we stand today, signed the Emancipation Proclamation. This momentous decree came as a great beacon light of hope to millions of Negro slaves who had been seared in the flames of withering injustice. It came as a joyous daybreak to end the long night of their captivity. But 100 years later, the Negro still is not free. One hundred years later, the life of the Negro is still sadly crippled by the manacles of segregation and the chains of discrimination. One hundred years later, the Negro lives on a lonely island of poverty in the midst of a vast ocean of material prosperity. One hundred years later the Negro is still languished in the corners of American society and finds himself in exile in his own land. And so we've come here today to dramatize a shameful condition. In a sense we've come to our nation's capital to cash a check. When the architects of our republic wrote the magnificent words of the Constitution and the Declaration of Independence, they were signing a promissory note to which every American was to fall heir. This note was a promise that all men - yes, black men as well as white men - would be guaranteed the unalienable rights of life, liberty and the pursuit of happiness. It is obvious today that America has defaulted on this promissory note insofar as her citizens of color are concerned. Instead of honoring this sacred obligation, America has given the Negro people a bad check, a check which has come back marked insufficient funds. But we refuse to believe that the bank of justice is bankrupt. We refuse to believe that there are insufficient funds in the great vaults of opportunity of this nation. And so we've come to cash this check, a check that will give us upon demand the riches of freedom and the security of justice. We have also come to his hallowed spot to remind America of the fierce urgency of now. This is no time to engage in the luxury of cooling off or to take the tranquilizing drug of gradualism. Now is the time to make real the promises of democracy. Now is the time to rise from the dark and desolate valley of segregation to the sunlit path of racial justice. Now is the time to lift our nation from the quick sands of racial injustice to the solid rock of brotherhood. Now is the time to make justice a reality for all of God's children. It would be fatal for the nation to overlook the urgency of the moment. This sweltering summer of the Negro's legitimate discontent will not pass until there is an invigorating autumn of freedom and equality. 1963 is not an end, but a beginning. Those who hope that the Negro needed to blow off steam and will now be content will have a rude awakening if the nation returns to business as usual. There will be neither rest nor tranquility in America until the Negro is granted his citizenship rights. The whirlwinds of revolt will continue to shake the foundations of our nation until the bright day of justice emerges. But there is something that I must say to my people who stand on the warm threshold which leads into the palace of justice. In the process of gaining our rightful place, we must not be guilty of wrongful deeds. Let us not seek to satisfy our thirst for freedom by drinking from the cup of bitterness and hatred. We must forever conduct our struggle on the high plane of dignity and discipline. We must not allow our creative protest to degenerate into physical violence. Again and again, we must rise to the majestic heights of meeting physical force with soul force. The marvelous new militancy which has engulfed the Negro community must not lead us to a distrust of all white people, for many of our white brothers, as evidenced by their

presence here today, have come to realize that their destiny is tied up with our destiny. And they have come to realize that their freedom is inextricably bound to our freedom. We cannot walk alone. And as we walk, we must make the pledge that we shall always march ahead. We cannot turn back. There are those who are asking the devotees of civil rights, when will you be satisfied? We can never be satisfied as long as the Negro is the victim of the unspeakable horrors of police brutality. We can never be satisfied as long as our bodies, heavy with the fatigue of travel, cannot gain lodging in the motels of the highways and the hotels of the cities. We cannot be satisfied as long as the Negro's basic mobility is from a smaller ghetto to a larger one. We can never be satisfied as long as our children are stripped of their selfhood and robbed of their dignity by signs stating: for whites only. We cannot be satisfied as long as a Negro in Mississippi cannot vote and a Negro in New York believes he has nothing for which to vote. No, no, we are not satisfied, and we will not be satisfied until justice rolls down like waters, and righteousness like a mighty stream. I am not unmindful that some of you have come here out of great trials and tribulations. Some of you have come fresh from narrow jail cells. Some of you have come from areas where your quest for freedom left you battered by the storms of persecution and staggered by the winds of police brutality. You have been the veterans of creative suffering. Continue to work with the faith that unearned suffering is redemptive. Go back to Mississippi, go back to Alabama, go back to South Carolina, go back to Georgia, go back to Louisiana, go back to the slums and ghettos of our Northern cities, knowing that somehow this situation can and will be changed. Let us not wallow in the valley of despair, I say to you today, my friends. So even though we face the difficulties of today and tomorrow, I still have a dream. It is a dream deeply rooted in the American dream. I have a dream that one day this nation will rise up and live out the true meaning of its creed: We hold these truths to be self-evident, that all men are created equal. I have a dream that one day on the red hills of Georgia, the sons of former slaves and the sons of former slave owners will be able to sit down together at the table of brotherhood. I have a dream that one day even the state of Mississippi, a state sweltering with the heat of injustice, sweltering with the heat of oppression will be transformed into an oasis of freedom and justice. I have a dream that my four little children will one day live in a nation where they will not be judged by the color of their skin but by the content of their character. I have a dream today. I have a dream that one day down in Alabama with its vicious racists, with its governor having his lips dripping with the words of interposition and nullification, one day right down in Alabama little black boys and black girls will be able to join hands with little white boys and white girls as sisters and brothers. I have a dream today. I have a dream that one day every valley shall be exalted, every hill and mountain shall be made low, the rough places will be made plain, and the crooked places will be made straight, and the glory of the Lord shall be revealed, and all flesh shall see it together. This is our hope. This is the faith that I go back to the South with. With this faith, we will be able to hew out of the mountain of despair a stone of hope. With this faith we will be able to transform the jangling discords of our nation into a beautiful symphony of brotherhood. With this faith we will be able to work together, to pray together, to struggle together, to go to jail together, to stand up for freedom together, knowing that we will be free one day. This will be the day when all of God's children will be able to sing with new meaning: My country, 'tis of thee, sweet land of liberty, of thee I sing. Land where my fathers died, land of the pilgrims' pride, from every mountainside, let freedom ring. And if America is to be a great nation, this must become true. And so let freedom ring from the prodigious hilltops of New Hampshire. Let freedom ring from the mighty mountains of New York. Let freedom ring from the heightening Alleghenies of Pennsylvania. Let freedom ring from the snowcapped Rockies of Colorado. Let freedom ring from the curvaceous slopes of California. But not only that, let freedom ring from Stone Mountain of Georgia. Let freedom ring from Lookout Mountain of Tennessee. Let freedom ring from every hill and molehill of Mississippi. From every mountainside, let freedom ring. And when this happens, and when we allow freedom ring, when we let it ring from every village and every hamlet, from every state and every city, we will be able to speed up that day when all of God's children, black men and white men, Jews and Gentiles, Protestants and Catholics, will be able to join hands and sing in the words of the old Negro spiritual: Free at last. Free at last. Thank God almighty, we are free at last.

Decoded: (the decoding accuracy is 99.8 %, the mispredicted characters are labeled in red)

Five score years ago, a great American, in whose symbolic shadow we stand today, signed the Emancipation Proclamation. This momentous decree came as a great beacon light of hope to millions of Negro slaves who had been seared in the flames of withering injustice. It came as a joyous daybreak to end the long night of their captivity. But 100 years later, the Negro still is not free. One hundred years later, the life of the Negro is still sadly crippled by the manacles of segregation and the chains of discrimination. One hundred years later, the Negro lives on a lonely island of poverty in the midst of a vast ocean of material prosperity. One hundred years later the Negro is still languished in the corners of American society and finds himself in exile in his own land. And so we've come here today to dramatize a shameful condition. In a sense we've come to our nation's capital to cash a check. When the architects of our republic wrote the magnificent words of the Constitution and the Declaration of Independence, they were signing a promissory note to which every American was to fall heir. This note was a promise that all men - yes, black men as well as white men - would be guaranteed the unalienable rights of life, liberty and the pursuit of happiness. It is obvious today that America has defaultcd on this promissory note insofar as her citizens of color are concerned. Instead of honoring this sacred obligation, America has given the Negro people a bad check, a check which has come back marked insufficient funds. But we refuse to believe that the bank of justice is bankrupt. We refuse to believe that there are insufficient funds in the great vaults of opportunity of this nation. And so we've come to cash this check, a check that will give us upon demand the riches of freedom and the security of justice. We have also come to his hallowed spot to remind America of the fierce urgency of now. This is no time to engage in the luxury of cooling off or to take the tranquilizing drug of gradualism. Now is the time to make real the promises of democracy. Now is the time to rise from the dark and desolate valley of segregation to the sunlit path of racial justice. Now is the time to lift our nation from the quick sands of racial injustice to the solid rock of brotherhood. Now is the time to make justice a reality for all of God's children. It would be fatal for the nation to overlook the urgency of the moment. This sweltering summer of the Negro's legitimate discontent will not pass until there is an invigorating autumn of freedom and equality. 1963 is not an end, but a beginning. Those who hope that the Negro needed to blow off steam and will now be content will have a rude awakening if the nation returns to business as usual. There will be neither rest nor tranquility in America until the Negro is granted his citizenship rights. The whirlwinds of revolt will continue to shake the foundations of our nation until the bright day of justice emerges. But there is something that I must say to my people who stand on the warm threshold which leads into the palace of justice. In the process of gaining our rightful place, we must not be guilty of wrongful de8ds. Let us not seek to satisfy our thirst for freedom by drinking from the cup of bitterness and hatred. We must forever conduct our struggle on the high plane of dignity and discipline. We must not allow our creative protest to degenerate into physical violence. Again and again, we must rise to the majestic heights of meeting physical force with soul force. The marvelous new militancy which has engulfed the Negro community must not lead us to a distrust of all white people, for many of our white brothers, as evidenced by their presence here today, have come to realize that their destiny is tied up with our destiny. And they have come to realize uhat their freedom is inextricably bound to our freedom. We cannot walk alone. And as we walk, we must make the pledge that we shall always march ahead. We cannot turn back. There are those who are asking the devotees of civil rights, when will you be satisfied? We can never be satisfied as long as the Negro is the victim of the unspeakable horrors of police brutality. We can never be satisfied as{long as our bodies, heavy with the fatigue of travel, cannot gain lodging in the motels of the highways and the hotels of thd cities. We cannot be satisfied as long as the Negro's basic mobility is from a smaller ghetto to a larger one. We can nevqr be satisfied as long as our children are stripped of their selfhood and robbed of their dignity by signs stating: for whites only. We cannot be satisfied as long as a Negro in Mississippi cannot vote and a Negro in New York believes he has nothing for which to vote. No, no, we are not satisfied, and we will not be satisfied until justice rolls down like waters, and righteousness like a mighty stream. I am not unmindful that some of you have come here out of great trials and tribulations. Some of you have come fresh from narrow jail cells. Some of you have come from areas where your quest for freedom left you battered by the storms of persecution and staggered by the winds of police brutality. You have been the veterans of creative suffering. Continue to work with the faith that unearned suffering is redemptive. Go back to Mississippi, go back to Alabama, go back to South Carolina, go back to Georgia, go back to Louisiana, go back to the slums and ghettos of our Northern cities, knowing

that somehow this situation can and will be changed. Let us not wallow in{the valley of despair, I say to you today, my friends. So even though we face the difficulties of today and tomorrow, I still have a dream. It is a dream deeply rooted in the American dream. I have a dream that one day this nation will rise up and live out the true meaning of its creed:{We hold these truths to be self-evident, that all men are created equal. I have a dream that one day on the red hills of Georgia, the sons of former slaves and the sons of former slave owners will be able to sit{down together at the table of brotherhood. I have a dream that one day even the state of Mississippi, a state sweltering with the heat of injustice, sweltering with the heat of oppression will be transformed into an oasis of freedom and justice. I have a dream that my four little children will one day live in a nation where they will not be judged by the color of their skin but by the content of their character. I have a dream today. I have a dream that one day down in Alabama with its vicious racists, with its governor having his lips dripping with the words of interposition and~nullification, one day right down in Alabama little black boys and black girls will be able to join hands with little white boys and white girls as sisters and brothers. I have a dream today. I have a dr**u**am that one day every valley shall be exalted, every hill and mountain shall b**8** made{low, the rough places will be made plain, and the crooked places will be made straight, and the glory of the Lord shall be revealed, and all flesh shall see it together. This is our hope. This is the faith that I go_back to the South with. With this faith, we will be able to hew out of the mountain of despair a stone of hope. With this faith we will be able to transform the jangling discords of our nation into a beautiful symphony of brotherhood. With this faith we will{be able to work together, to pray together, to struggle together, to go to jail together, to stand up for freedom together, knowing that we will be free one day. This will be the day when all of God's children will be able to sing with new meaning: My country, 'tis of thee, sweet land of liberty, of thee I sing. Land where my fathers died, land of the pilgrims' pride, from every mountainside, let freedom ring. And if America is to be a great nation, this must become true. And so let freedom ring from the prodigious hilltops of New Hampshire. Let freedom ring from the mighty mountains of New York. Let freedom ring from the heightening Alleghenies of Pennsylvania. Let freedom ring from the snowcapped Rockies of Colorado. Let freedom ring from the curvaceous slopes of California. But not only that, let freedom ring from Stone Mountain of Georgia. Let freedom ring from Lookout Mountain of Tennessee. Let freedom ring from every hill{and molehill of Mississippi. From every mountainside, let freedom ring. And when this happens, and when we allow freedom ring, when we let it ring from every village and every hamlet, from every state and every city, we will be able to speed up that day when all of God's children, black men and white men, Jews and Gentiles, Protestants and Catholics, will be able to join hands and sing in the words of the old N**c**gro spiritual: Fr**8**e at last. Free at last. Thank God almighty, we are free at last.

Appendix C

Encoding “Auguries of Innocence” in Emorfi and decoding it back in English

Each character was encoded in a randomly selected pattern from its corresponding class (see Appendix A). Next, the patterns were assembled in order into a video. To accommodate majority voting, the above process was repeated four times and the new video was concatenated to the end of the existing one. When decoding, we used a trained CNN combined with majority voting, 99.6% of the text was correctly decoded.

Auguries of Innocence

By William Blake

1863

Original (Poetry foundation):

To see a World in a Grain of Sand
And a Heaven in a Wild Flower
Hold Infinity in the palm of your hand
And Eternity in an hour
A Robin Red breast in a Cage
Puts all Heaven in a Rage
A Dove house filld with Doves & Pigeons
Shudders Hell thr' all its regions
A dog starvd at his Masters Gate
Predicts the ruin of the State
A Horse misusd upon the Road
Calls to Heaven for Human blood
Each outcry of the hunted Hare
A fibre from the Brain does tear
A Skylark wounded in the wing
A Cherubim does cease to sing
The Game Cock clipd & armd for fight
Does the Rising Sun affright
Every Wolfs & Lions howl
Raises from Hell a Human Soul
The wild deer, wandring here & there
Keeps the Human Soul from Care
The Lamb misusd breeds Public Strife
And yet forgives the Butchers knife
The Bat that flits at close of Eve
Has left the Brain that wont Believe
The Owl that calls upon the Night
Speaks the Unbelievers fright
He who shall hurt the little Wren
Shall never be belovd by Men
He who the Ox to wrath has movd
Shall never be by Woman lovd
The wanton Boy that kills the Fly
Shall feel the Spiders enmity
He who torments the Chafers Sprite
Weaves a Bower in endless Night
The Catterpiller on the Leaf
Repeats to thee thy Mothers grief

Kill not the Moth nor Butterfly
For the Last Judgment draweth nigh
He who shall train the Horse to War
Shall never pass the Polar Bar
The Beggars Dog & Widows Cat
Feed them & thou wilt grow fat
The Gnat that sings his Summers Song
Poison gets from Slanders tongue
The poison of the Snake & Newt
Is the sweat of Envys Foot
The poison of the Honey Bee
Is the Artists Jealousy
The Princes Robes & Beggars Rags
Are Toadstools on the Misers Bags
A Truth thats told with bad intent
Beats all the Lies you can invent
It is right it should be so
Man was made for Joy & Woe
And when this we rightly know
Thro the World we safely go
Joy & Woe are woven fine
A Clothing for the soul divine
Under every grief & pine
Runs a joy with silken twine
The Babe is more than swadling Bands
Throughout all these Human Lands
Tools were made & Born were hands
Every Farmer Understands
Every Tear from Every Eye
Becomes a Babe in Eternity
This is caught by Females bright
And returnd to its own delight
The Bleat the Bark Bellow & Roar
Are Waves that Beat on Heavens Shore
The Babe that weeps the Rod beneath
Writes Revenge in realms of Death
The Beggars Rags fluttering in Air
Does to Rags the Heavens tear
The Soldier armd with Sword & Gun
Palsied strikes the Summers Sun
The poor Mans Farthing is worth more
Than all the Gold on Africs Shore
One Mite wrung from the Labrers hands
Shall buy & sell the Misers Lands
Or if protected from on high
Does that whole Nation sell & buy
He who mocks the Infants Faith
Shall be mockd in Age & Death
He who shall teach the Child to Doubt
The rotting Grave shall neer get out
He who respects the Infants faith
Triumphs over Hell & Death
The Childs Toys & the Old Mans Reasons
Are the Fruits of the Two seasons
The Questioner who sits so sly
Shall never know how to Reply

He who replies to words of Doubt
Doth put the Light of Knowledge out
The Strongest Poison ever known
Came from Caesars Laurel Crown
Nought can Deform the Human Race
Like to the Armours iron brace
When Gold & Gems adorn the Plow
To peaceful Arts shall Envy Bow
A Riddle or the Crickets Cry
Is to Doubt a fit Reply
The Emmets Inch & Eagles Mile
Make Lame Philosophy to smile
He who Doubts from what he sees
Will neer Believe do what you Please
If the Sun & Moon should Doubt
Theyd immediately Go out
To be in a Passion you Good may Do
But no Good if a Passion is in you
The Whore & Gambler by the State
Licencd build that Nations Fate
The Harlots cry from Street to Street
Shall weave Old Englands winding Sheet
The Winners Shout the Losers Curse
Dance before dead Englands Hearse
Every Night & every Morn
Some to Misery are Born
Every Morn and every Night
Some are Born to sweet delight
Some are Born to sweet delight
Some are Born to Endless Night
We are led to Believe a Lie
When we see not Thro the Eye
Which was Born in a Night to perish in a Night
When the Soul Slept in Beams of Light
God Appears & God is Light
To those poor Souls who dwell in Night
But does a Human Form Display
To those who Dwell in Realms of day

Decoded: (the decoding accuracy is 99.6%, the mispredicted characters are labeled in red)

To see a World in a Grain of Sand
And a Heaven in a Wild Flower
Hold Infinity in the palm of your hand
And Eternity in an hour
A Robin Red breast in a Cage
Puts all Heaven*****in a Rage
A Dove house filld with Doves & Pigeons
Shudders Hell thr' all its regions
A dog starvd at his Masters Gate
Predicts the ruin of the State
A Horse misusd upon the Road
Calls to Heaven for Human blood
Each outcry of the hunted Hare
A fibre from the Brain does tear
A Skylark wounded in the wing

A Cherubim does cease to sing
The Game Cock clipd & armd for fight
Does the Rising Sun affright
Every Wolfs & Lions howl
Raises from Hell a Human Soul
The wild deer, wandring here & there
Keeps the Human Soul from Care
The Lamb misusd breeds Public Strife
And yet forgives the Butchers knife
The Bat that flit**u** at close of Eve
Has left the Brain that wont Believe
The Owl t**l**at calls upon the Night
Speaks the Unbelievers fright
He who shall hurt the little Wren
Shall never be belovd by Men
He who the Ox to**{**wrath has movd
Shall never be by Woman lovd
The wanton Boy that kills the Fly
Shall feel the Spiders enmity
He who torments the Chafers Sprite
Weaves a Bower in endless Night
The Catterpiller on the Leaf
Repeats to thee thy Mothers grief
Kill not the Moth nor Butterfly
For the Last Judgment draweth nigh
He who shall train the Horse to War
Shall never pass the Polar Bar
The Beggars Dog & Widows Cat
Feed them & thou wilt grow fat
The Gnat that sings his Summers Song
Poison gets from Slanders tongue
The poison of the Snake & Newt
Is the sweat of Envys Foot
The poison of the Honey Bee
Is the Artists Jealousy
The Princes Robes & Beggars Rags
Are Toadstools on the Misers Bags
A Truth thats told with bad intent
Beats all the Lies you can invent
It is right it should be so
Man was made for Joy & Woe
And when this we rightly know
Thro the World we safely go
Joy & Woe are woven fine
A Clothing for the**?**soul divine
Under every grief & pine
Runs a joy with silken twine
The Babe is more than swadling Bands
Throughout all these Human Lands
Tools were made &**]**Born were hands
Every Farmer Understands
Every Tear from Every Eye
Becomes a Babe in Eternity
This is caught by Female**u** bright
And returnd to its own delight
The Bleat the Bark Bellow & Roar

Are Waves that Beat on Heavens Shore
The Babe~that weeps the Rod beneath
Writes Revenge in realms{of Death
The Beggars Rags fluttering in Air
Does to Rags the Heavens tear
The Soldier armd with Sword & Gun
Palsied strikes the Summers Sun
The poor Mans Farthing is worth more
Than all the Gold on Africs Shore
One Mite wrung from the Labrers hands
Shall buy & sell the Misers Lands
Or if p**t**otected from on high
Does that whole Nation sell & buy
He who mocks the Infants Faith
Shall be mockd in Ag**c** & Death
He who shall teach the Child to Doubt
The rotting Grave shall neer get out
He who respects the Infants faith
Triumphs over Hell & Death
The Childs Toys & the Old Mans Reasons
Are the Fruits of the Two seasons
The Questioner who sits**?**so sly
Shall never know how to Reply
He who replies to words of Doubt
Doth put the Light of Knowledge out
The Strongest Poison ever known
Came from Caesars Laurel~Crown
Nought can Deform the Human Race
Like to the Armours iron brace
When Gold & Gems adorn the Plow
To peaceful Arts shall Envy Bow
A Riddle or the Crickets Cry
Is to Doubt a fit Reply
The Emmets Inch & Eagles Mile
Make Lame Philosophy to smile
He{who Doubts from what he sees
Will neer Believe do what you Please
If]the Sun & Moon should Doubt
Theyd immediately Go out
To be in a Passion you Good may Do
But no Good if a Passion is in you
The Whore & Gambler by the State
Licencd build that Nations Fate
The Harlots cry from Street to Street
Shall weave Old Englands winding Sheet
The Winners Shout the Losers Curse
Dance before dead Englands Hearse
Every Night & every Morn
Some to Misery are Born
Every Morn and every Night
Some are Born to sweet delight
Some are Born to sweet delight
Some are Born to Endless Night
We are led to Believe a Lie
When we see not Thro the Eye
Which was Born in a Night to perish in a Night

When the Soul Sl**c**pt in Beams of Light
God Appears & God is Light
To those poor Souls who dwell in Night
But does a Human Form Display
To those who Dwell in Realms of day

Appendix D

Encoding the GFP protein sequence in patterns and decoding it back in amino acid

A similar approach for generating Emorfi was used to encode and decode protein sequences. Each of the 20 common amino acids was converted into a binary representation and then converted into a unique initial configuration. 1000 images were generated through mathematical simulation for each amino acid.

To encode GFP sequence, each amino acid was encoded in a randomly selected pattern from its corresponding class. Next, the patterns were assembled in order into a video. To accommodate majority voting, the above process was repeated four times and the new video was concatenated to the end of the existing one. When decoding, we used a trained CNN combined with majority voting, 100% of the sequence was correctly predicted.

GFP protein sequence

Original (Uniprot):

MSKGEELFTGVVPILVELDGDVNGHKFSVSGEGEGDATYGKLTLKFICTTGKLPVPWPT
LVTTFSYGVQCFSRYPDHMKQHDFFKSAMPEGYVQERTIFFKDDGNYKTRAEVKFEGDTLVNRI
ELKGIDFKEDGNILGHKLEYNYNSHNVYIMADKQKNGIKVNFKIRHNIEDGSVQLADHYQQNTPIG
DGPVLLPDNHYLSTQSALSKDPNEKRDHMVLLEFVTAAGITHGMDELYK

Decoded: (the decoding accuracy is 100%)

MSKGEELFTGVVPILVELDGDVNGHKFSVSGEGEGDATYGKLTLKFICTTGKLPVPWPT
LVTTFSYGVQCFSRYPDHMKQHDFFKSAMPEGYVQERTIFFKDDGNYKTRAEVKFEGDTLVNRI
ELKGIDFKEDGNILGHKLEYNYNSHNVYIMADKQKNGIKVNFKIRHNIEDGSVQLADHYQQNTPIG
DGPVLLPDNHYLSTQSALSKDPNEKRDHMVLLEFVTAAGITHGMDELYK
