# Supplementary material for: Distributed information encoding and decoding using self-organized spatial patterns
Source: Patterns (N Y). 2022 Sep 23;3(10):100590. doi: 10.1016/j.patter.2022.100590 (PMC9583124; doi:10.1016/j.patter.2022.100590)
Supplement: Document S2. Article plus supplemental information [file mmc5.pdf]

Article

Patterns

Distributed information encoding and decoding using self-organized spatial patterns

Highlights

- Self-organized patterns can be used for secure information encoding
- A machine learning-mediated decoding method is proposed
- Encoding capability and security are tunable through modulating system properties

Authors

Jia Lu, Ryan Tsoi, Nan Luo, ..., Alison Zhang, Neil Zhenqiang Gong, Lingchong You

Correspondence

you@duke.edu

In brief

This work demonstrates the feasibility of using self-organized patterns for secure information encoding. It advances the use of these systems by using machine learning-mediated decoding. It provides an empirical analysis that would allow for fast prototyping and implementation of dynamical system-based communication platforms, and a means to measure the convergence of biological system outputs.

Lu et al., 2022, Patterns *3*, 100590
October 14, 2022 © 2022 The Author(s).
https://doi.org/10.1016/j.patter.2022.100590

CellPress

Patterns

CellPress
OPEN ACCESS

Article

Distributed information encoding and decoding using self-organized spatial patterns

Jia Lu,[1] Ryan Tsoi,[1] Nan Luo,[1] Yuanchi Ha,[1] Shangying Wang,[1] Minjun Kwak,[2,8] Yasa Baig,[3,8] Nicole Moiseyev,[2,8] Shari Tian,[4,8] Alison Zhang,[5,8] Neil Zhenqiang Gong,[2,5] and Lingchong You[1,6,7,9,*]
[1]Department of Biomedical Engineering, Duke University, Durham, NC 27708, USA
[2]Department of Computer Science, Duke University, Durham, NC 27708, USA
[3]Department of Physics, Duke University, Durham, NC 27708, USA
[4]Department of Statistical Science, Duke University, Durham, NC 27708, USA
[5]Department of Electrical and Computer Engineering, Duke University, Durham, NC 27708, USA
[6]Center for Genomic and Computational Biology, Duke University, Durham, NC 27708, USA
[7]Department of Molecular Genetics and Microbiology, Duke University School of Medicine, Durham, NC 27708, USA
[8]These authors contributed equally
[9]Lead contact
*Correspondence: you@duke.edu
https://doi.org/10.1016/j.patter.2022.100590

THE BIGGER PICTURE Self-organized patterns are ubiquitous in biology. They arise from interactions in and between cells, and with the environment. These patterns are often used as a composite phenotype to distinguish cell states and environment conditions. Conceptually, pattern generation under an initial condition is encoding; discerning the initial condition from the pattern represents decoding. Inspired by these examples, we develop a scheme, integrating mathematical modeling and machine learning, to use self-organization for secure and accurate information encoding and decoding. We show that this strategy is applicable to non-biological dynamical systems. We further demonstrate the scalability of the scheme by generating a complete mapping of the standard English keyboard, allowing encoding of English text. Our work serves as an example of nature-inspired computation.

1 2 3 4 5 **Proof-of-Concept:** Data science output has been formulated, implemented, and tested for one domain/problem

SUMMARY

Dynamical systems often generate distinct outputs according to different initial conditions, and one can infer the corresponding input configuration given an output. This property captures the essence of information encoding and decoding. Here, we demonstrate the use of self-organized patterns that generate high-dimensional outputs, combined with machine learning, to achieve distributed information encoding and decoding. Our approach exploits a critical property of many natural pattern-formation systems: in repeated realizations, each initial configuration generates similar but not identical output patterns due to randomness in the patterning process. However, for sufficiently small randomness, different groups of patterns that arise from different initial configurations can be distinguished from one another. Modulating the pattern-generation and machine learning model training can tune the tradeoff between encoding capacity and security. We further show that this strategy is scalable by implementing the encoding and decoding of all characters of the standard English keyboard.

INTRODUCTION

Information encoding is a process of converting information, such as text and images, from its original representation to an output format following defined rules. Dynamical systems have this information encoding capability as they can generate specific outputs according to given inputs. Conversely, decoding can be achieved if one can infer the input corresponding to an output. Depending on the system, decoding could be obvious, challenging, or impossible. As self-organization systems can

This is an open access article under the CC BY license (http://creativecommons.org/licenses/by/4.0/).

generate high-dimensional outputs, they are particularly useful for encoding rich information.

One example is to use cellular automaton (CA) that converts a grid of cells from a simple initial configuration into a self-organized sequence or spatial pattern according to a set of update rules.[1] Wolfram proposed to use a chaotic rule to generate random sequences to encode information.[2,3] Here, the encoding is deterministic—each initial configuration corresponds to a unique output pattern. Because of the chaotic nature of the rule, however, decoding the input from a given output pattern is computationally prohibitive without prior knowledge of the update rules. As such, the system in theory can serve as the foundation for digital cryptography.[4–8]

While making the encoding secure, however, the chaotic nature of the above example can limit its application. Like other dynamical systems exhibiting deterministic chaos, the final patterns generated by CA are extremely sensitive to perturbations and lack statistical regularities.[9,10] As such, a minute change in the initial configuration or the encoding process can lead to drastically different final patterns (a phenomenon termed the *avalanche effect*[11]). Unless the encoding and transmission are noise-free, the decoding is prone to errors *even if the rules are known*.[12]

In contrast to these chaotic systems, many natural systems are convergent. That is, for the same or similar input configurations and environmental conditions, the final patterns share global similarity despite local variances. This property is sometimes referred to as "edge of chaos."[13] Examples are chemical reaction[14] and cortical networks.[15] Many biological patterning systems also fall into this category. Despite minute variances, coat patterns are largely determined by animal genomes and allow identification of different species. In microbes, the same bacterial strain can grow into colonies with distinct shapes and sizes under different growth conditions.[16,17] Consequently, colony morphology can serve as a crude signature to distinguish environmental conditions and chemical cues, as well as the stage of infectious diseases.[18,19] Despite these empirical examples, the potential and limitations of information encoding and decoding using biological self-organization remain unexplored. Here, we use these systems to establish distributed information encoding. Coupled with machine learning (ML)-mediated decoding, our system illustrates a scalable strategy for information encoding and decoding with quantifiable reliability and security (Figure 1A).

RESULTS

Criteria for choosing an encoding system

Any dynamical systems, including those generating self-organized patterns, can serve as the foundation for information encoding and decoding. However, to ensure secure encoding and reliable decoding, we reason that the system dynamics need to meet a set of heuristic criteria. First, the output patterns are sufficiently complex and diverse such that different initial configurations would generate distinguishable output patterns. Second, the pattern generation is subject to stochasticity but remains convergent. That is, in repeated pattern-generation processes, the same initial configuration with small noise or perturbations should generate output patterns that are approximately the same but differ in minor details. Importantly, the differences between patterns generated from replicated simulations should be smaller than those between patterns generated from different inputs. Third, while different groups of patterns arising from different initial conditions can be decoded by a properly constructed decoder, their differences are difficult to discern by the naked eye. We note that the degree by which different groups of patterns can be distinguished often has to be established empirically (if a reliable decoder can indeed be constructed).

As a proof of principle, we focus on a coarse-grained model of self-organized pattern formation (Figure 1, also see "mathematical modeling" in methods). The model was developed to simulate qualitative aspects of branching dynamics of *Pseudomonas aeruginosa* colony growth.[20] In it, each simulation initiates from a predefined cell seeding configuration and the cells develop into a branching colony (Figure S1). The patterning process is influenced by two sources of random noise. One comes from the variability in the initial distribution of seeding cells; the other comes from the underlying growth kinetics. With appropriate choice of parameters (including noise levels), the patterning dynamics satisfy all criteria listed above.

In addition, another rationale for choosing this model is its simplicity and versatility. It can generate diverse patterns by adjusting model parameters and be solved in a computationally efficient manner (one simulation takes several minutes on a cluster compute node to solve). These features allow us to probe this platform's security, reliability, and scalability (see "tradeoff among encoding capacity, security, and decoding reliability").

Distributed encoding and decoding by spatial patterns

To demonstrate encoding, we represent a dictionary of 15 characters—letters A–E and numbers 0–9—using binary numbers 0001–1111 (Table S1). Each binary number then corresponds to a seeding configuration of cells in a braille-like array at time 0 (Figure 1B): a digit "1" corresponds to a spot seeding indicating the presence of cells, whereas a digit "0" indicates no cells. In each simulation, the colony grows from its initial configuration into a final pattern. As mentioned above, the simulation is subject to two noise sources: the variability in seeding and during growth. The former could originate from the marginal but unavoidable uneven cell seeding, and the latter could originate from the inherent heterogeneity of cell gene expression, motility, or small external perturbation. Therefore, repeated simulations from the same initial seeding configuration generate similar final patterns with minor differences, which *collectively* encode the identity of the input configuration (Figure 1C). We chose to encode in seeding configuration because of its simplicity, one may also choose to encode in other parameters influencing pattern formation.

We configure our simulations such that neither the mapping between the initial configurations and the colony patterns nor the difference between patterns corresponding to different inputs is obvious to the naked eye. To allow reliable decoding, we need a robust method to navigate through this visual complexity. A direct method is brute-force search, whereby all the possible patterns for each initial configuration are simulated to establish an empirical mapping between the input and the output. While apparently straightforward, this approach is computationally prohibitive and impractical because the training

CellPress
OPEN ACCESS

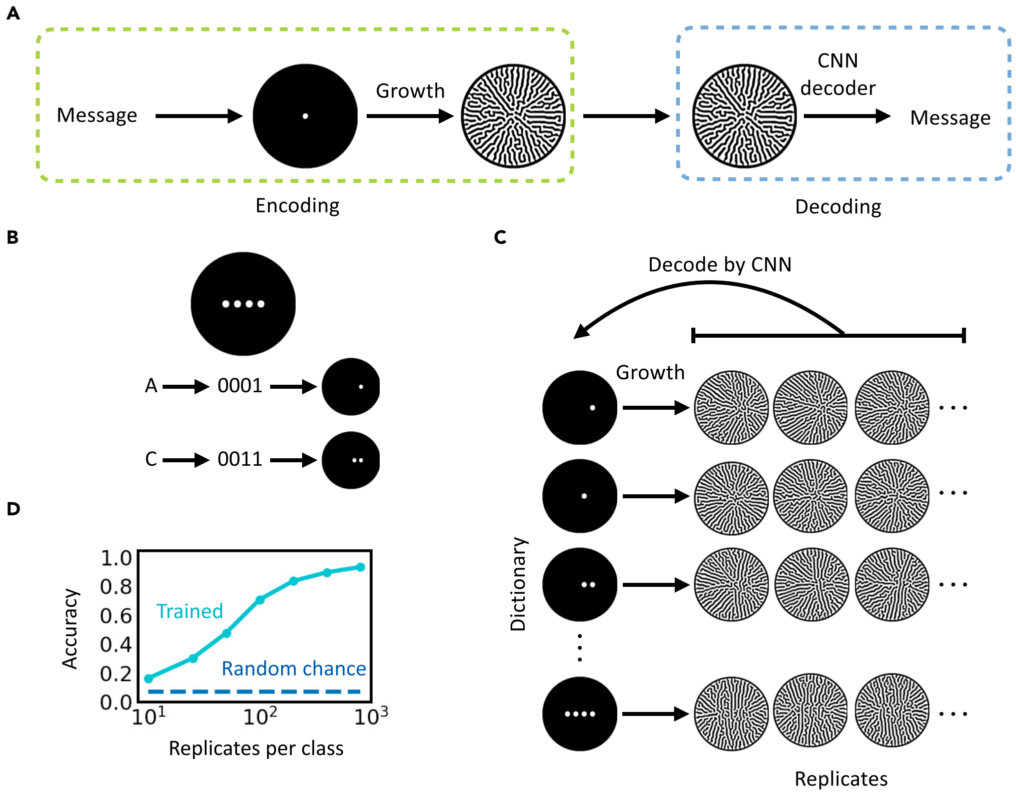

Figure 1. Distributed encoding and decoding using self-organized patterns

(A) The encoding and decoding scheme. To encode, a message is converted into cell seeding configuration followed by colony growth, during which a colony pattern develops. To decode, the colony pattern of interest is fed into a trained CNN that converts the pattern into the original message.

(B) Predefined braille-like cell seeding arrangement. For a dictionary consisting of 15 characters (A–E and 0–9), we need a minimum 4-digit spot array (top). The characters (e.g., "A" and "C") are first converted into a 4-digit binary number, then converted into a seeding configuration. For a given digit, if it is 1, cells are "inoculated" within the corresponding spot and if it is a 0, no cell is inoculated.

(C) One-to-many mapping between seeding configuration and spatial patterns. Pattern formation is subject to minor biological noise, which includes heterogeneity in cell seeding, external perturbation, and variability in cell phenotype during growth process. The noise is amplified by the branching mechanism. Hence, patterns evolved from the same configuration share qualitative similarity but are different in detail. A well-trained CNN should navigate through this mapping and be able to decode the patterns as the corresponding character. For CNN training, the dataset is composed of equal number of replicates of patterns developed from all seeding configurations.

(D) Relationship between the number of replicates of the training set and CNN accuracy. The CNN was trained on a balanced dataset that contains 15 distinct characters. The numerical simulation used the default parameter values (see "mathematical modeling" in methods) and intermediate growth noise (signal-to-noise ratio = 3.5). The CNN decoding accuracy increases as the number of available replicates increases. The accuracy is significantly higher than random chance (1/the size of the dictionary).

patterns are 8-bit, 80 × 80 pixels grayscale images, resulting in up to $2^{8\times80\times80} \sim 10^{15412}$ possible patterns.

Alternatively, image classification using convolutional neural networks (CNNs) has been successful for numerous applications.[21–23] Through observing sufficient examples, a CNN learns to cluster images by their categories. Here, we built a CNN to decode the colony patterns via multiclass classification (Figure S2, see "CNN training" in methods). During training, our CNN decoder takes pattern images (generated by repeated simulations) as input and updates its trainable parameters to classify patterns based on initial seeding configurations. With sufficient replicates in each class, our trained CNN was able to distinguish patterns corresponding to the 15 characters with high accuracy (Figure 1D). For instance, greater than 93% of decoding accuracy can be achieved by having 800 replicate patterns in the training set.

In an actual application of this encoding/decoding strategy, we assume the channel is public while the pattern generator, model parameters, training set, and the trained CNNs are private to the end users (Figure 1A). The recipient chooses the correct, trained CNN to decode a pattern according to the model parameters transmitted through another private channel (not shown in the figure) as prior knowledge.

Tradeoff among encoding capacity, security, and decoding reliability

In this platform, we aim to maximize the capability of the patterns to encode information, termed *encoding capacity*, and our platform's robustness against data leakage to unauthorized parties, termed *encoding security*. We consider that a system has higher encoding capacity if it can encode more characters correctly with adequate data, while we consider our encoding scheme

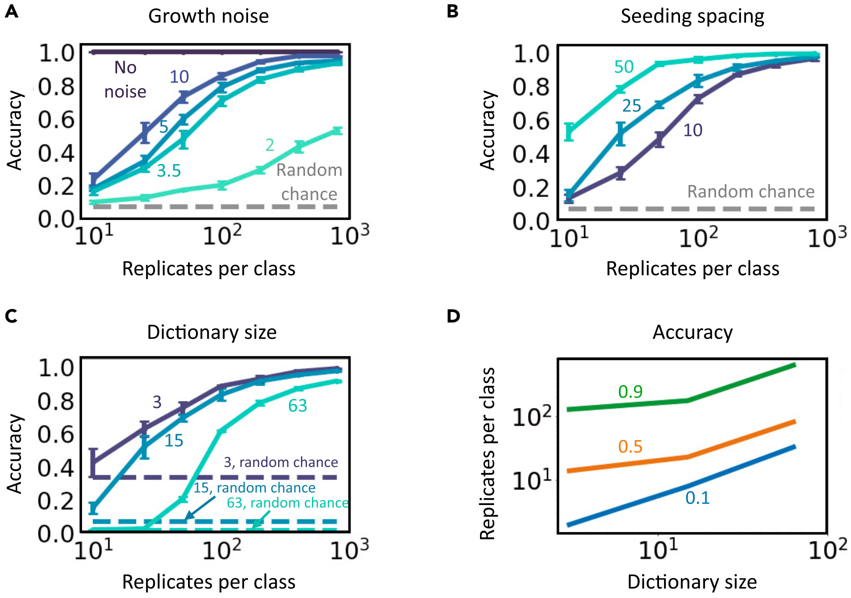

Figure 2. Tradeoff between encoding capacity, security, and decoding reliability

We present the prediction performance of CNNs trained on branching patterns with different parameterizations. Specifically, (A) we fixed the seeding noise such that the only source of noise is growth. The magnitude of growth noise is modulated through changing the signal-to-noise ratio (SNR) of the growth kernel. The higher SNR is, the lower the noise level is. We present the results on datasets with no noise, SNR = 2, 3.5, 5, and 10, respectively. (B) We simulated patterns using seeding spacing = 10, 25, and 50, respectively, which represent from small to large spacing. As the spacing increases, patterns corresponding to different initial configurations become more dissimilar.

(C) We simulated datasets of 3, 15, and 63 characters using 2-, 4-, and 6-bit predefined braille-like seeding arrays, respectively, while keeping all else as the default. Overall, the decoding accuracy increases as the number of replicates per class increases, and it significantly exceeds the corresponding accuracy by random guessing. The only exception is in the absence of growth noise, in which case the patterns are identical thus the decoding is trivial. Notably, when the patterns become more complicated (e.g., larger growth noise, smaller spacing, or larger dictionary), more data are required to reach the same accuracy. Data are represented as mean ± standard deviation.

(D) Required training replicates per class as a function of dictionary size. The green, orange, and blue lines represent accuracy of 0.9, 0.5, and 0.1, respectively. The required data size increases exponentially as the desired accuracy increases.

being more secure when the attacker cannot build a successful decoder from the leaked data. For example, the accuracy of a separate decoder built on only 10 replicates per class drops to less than 20% (Figure 1D), which is only slightly better than random guessing (1/15). Note that the efficacy of our platform depends on the complexity of the generated patterns, our desired accuracy, and the amount of available training data.

We can tune our scheme's performance by modulating parameters in the pattern-generation model. We constructed 16 simulated training datasets of diverse patterns by tuning these 2 parameters (see "mathematical modeling" in methods, Figure S3A). Based on their final appearance, we categorized our results into three subgroups: disk-like (a large disk occupying the entire growth domain), trivial (final pattern is identical to initial configuration), and branching. Disk-like colonies cannot be distinguished regardless of the training data size—thus, the input information was obscured and "lost" after growth (Figures S3B–S3D). Conversely, trivial patterns allow perfect but insecure decoding since the reverse mapping is obvious. Ultimately, the intricate branching patterns allow secure encoding and reliable decoding as demonstrated previously.

We can also modulate encoding capacity and security by tuning the noise during the patterning process. Without noise, one pattern per input is sufficient for perfect decoding as long as output patterns are distinguishable (Figure 2A). Too much noise would introduce too many variations in the replicate patterns generated from each input. If these intra-category variations (between replicate patterns) approach or exceed the inter-category differences (between sets of patterns corresponding to different inputs), the decoding accuracy would deteriorate significantly (Figure 2A). Depending on the magnitude of the noise, this loss in accuracy can be alleviated by increasing the number of replicate patterns per class. A similar tradeoff exists for other parameters as well, such as the spacing between spots in the initial configuration (Figure 2B). When spacing decreases, patterns grown from different configurations appear more alike and indistinguishable. Moreover, a larger dictionary with all else being equal would also reduce the decoding accuracy (Figure 2C). Again, expanding the number of replicate patterns per class can compensate for losses in accuracy, thus increasing the encoding capacity (Figure 2D). Similar tradeoff was also observed in patterns arrested from growth at different time points (see "temporal information encoding and decoding" in supplemental information).

In principle, the encoding-decoding scheme is applicable to any dynamical systems where the input-output mapping satisfies the criteria listed above. To illustrate this point, we chose an elementary CA model with weakly chaotic dynamics[9] (see "encoding and decoding using elementary cellular automaton" in supplemental information). Given the set of rules, we chose the model parameters (including noise levels) such that the resulting dynamics can allow secure encoding and reliable decoding. Again, we encoded characters in binary numbers, which is then converted into 1D initial configuration in a similar manner as in 2D. Noise was imposed on the initial sequence, and the latter develops into a final sequence following the evolution rules (Figure S4). A feedforward neural network was trained to code the final sequence. As expected, higher complexity leads to worse decoding accuracy, and it can be remedied by increasing training data size (Figure S5).

Enhancing encoding security and integrity

To enhance security, we evaluated utilizing encryption to prevent unauthorized access during communication. A secret key is

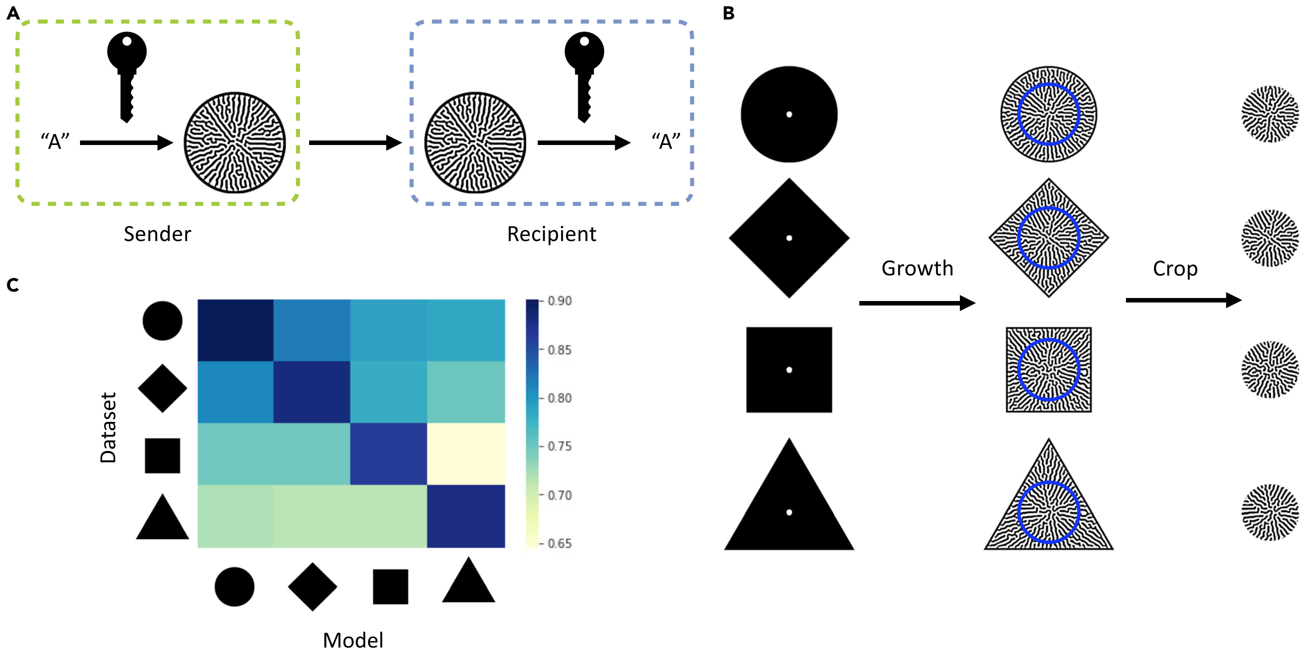

Figure 3. Encryption using growing domain shape as the secret key

(A) Encryption scheme. A secret key is used to convert a message (e.g., "A") to a self-organized pattern, and the knowledge of it is required to reliably convert the pattern back to the original message. For our ML-mediated decoding method, the information on the secret key allows the designated recipient to choose the correct, trained CNN to decode the received pattern.

(B) Training data generation and preprocessing. For each encoding character, we computationally seeded cells on growing domains of different shapes (left) and let them grow into spatial patterns over the entire field (middle). The centers of the colonies (within the blue circles) were cropped to remove the information of the growth domain (right), and then used for CNN training.

(C) Effectiveness of encryption when growth domain shape is the secret key. Four CNN models were trained independently on datasets encrypted by circular, diamond, square, and triangular growth domains, respectively. The heatmap shows their decoding accuracies on each dataset. Only the model trained on the corresponding dataset can decode at the highest accuracy.

implemented during encoding and successful decoding requires the correct key (Figure 3A). For pattern formation systems, the geometry of the patterning domain is a feasible choice of secret key as it can influence the patterning process and is easily tunable.[24–26] In our system, the boundary suppresses bacteria colonization, and the strength of the impact decreases exponentially as the distance from every location in the colony to the boundary increases (see "encryption dataset generation" in methods). As such, the boundary exhibits a time-invariant, long-range, and weak inhibitive force on colony expansion. As this force is anisotropic due to asymmetric boundary geometry, the patterns are encrypted by the domain shape.

To test this notion, we generated patterns within different boundary shapes. For each shape, the resulting patterns would occupy the entire space. We removed the information of the boundary in the output by cropping out a smaller, circular area at the center of each pattern (Figure 3B). We found that only the decoders trained on the correct datasets can decode at high accuracy (Figure 3C), indicating that knowledge of the domain shape (i.e., the secret key) is critical for selecting the right CNN decoder to accurately decode. Note that since the x and y axes are datasets and models, respectively, we do not expect symmetric accuracies in the off-diagonal cases. Similarly, we evaluated the potential of other secret key choices, such as the seeding spacing (Figure S7) and patterning domain size.

We have also considered the threat to information integrity during communication, in which the attackers could alter the output patterns or replace them with fake ones, thus deceiving the intended information receiver. We demonstrated that the noise in the patterning dynamics could be used to ensure the integrity (see "authenticating patterns using noise signatures" in supplemental information). In brief, the noise leaves a unique signature for each correct pattern, which can be used to authenticate a received pattern.

Improving decoding performance by ensemble learning

All else being equal, the reliability of decoding can be improved by increasing the number of replicates per class when training the decoder. However, the degree of improvement diminishes for an increasing number of replicates (Figure 2C). For instance, for a dictionary of 63 characters, the decoding accuracy increases by ~30-fold by increasing the number of replicates from 10 to 100; it only increases by ~1.5-fold by increasing from 100 to 800. To more effectively use the available data, we adopted ensemble learning—a class of ML techniques.[27–29]

Staked generalization combines the knowledge learned by individual ML models (base model) for better prediction.[30–33] We first trained multiple-base CNN decoders on a dataset with random initialization using the same protocol in the previous sections, then trained an ensemble decoder to combine their

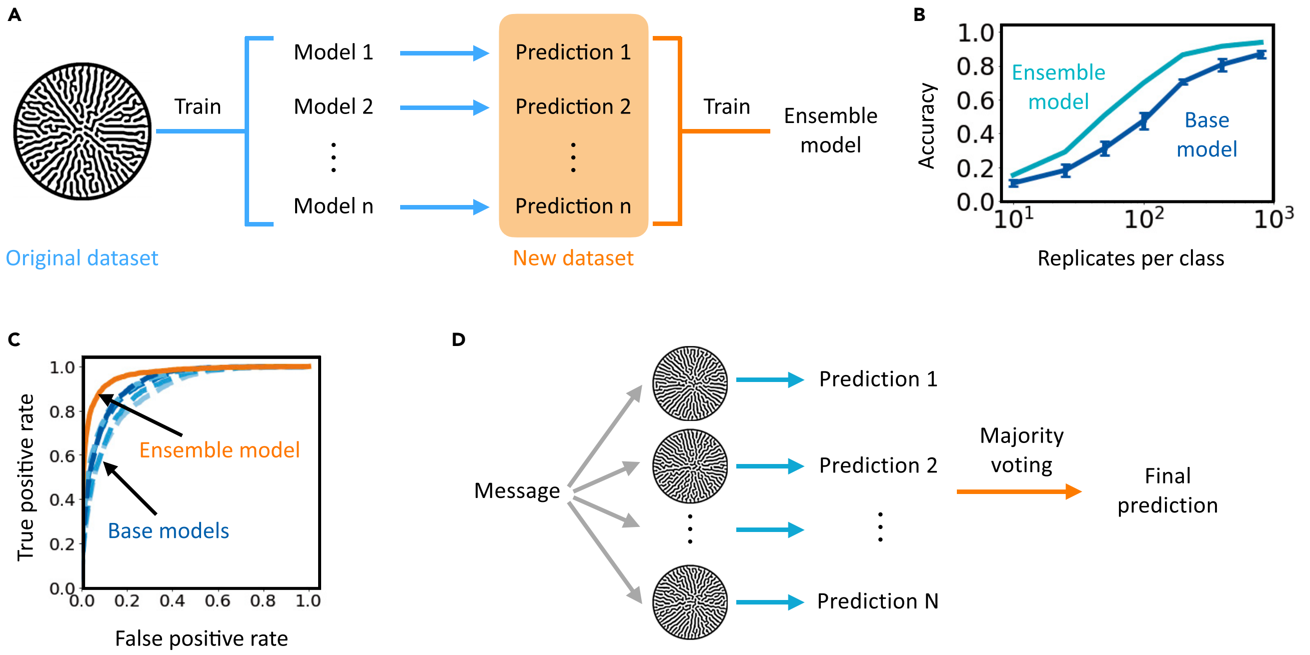

Figure 4. Using ensemble learning to improve decoding accuracy

(A) Training procedure of the ensemble model. The training is done in two steps. First, we train multiple-base CNN decoders on a dataset as described in the previous sections. Then their predictions on the training set and the corresponding class labels constitute a new dataset. In the second step, we train an ensemble model from scratch using the new dataset.

(B) Decoding accuracy of ensemble and base models. Here, a logistic regression ensemble model was trained with five base models. The ensemble model outperforms the base models regardless of the training data size. Notable improvement in accuracy occurs when a moderate amount of data was available for training, whereas the improvement is less significant with adequate or scarce data. Data are represented as mean ± standard deviation.

(C) ROC curve of ensemble and base models (orange, ensemble model; shades of blue, base models). The ROC curves were computed for each encoding character and then averaged over all classes to reflect the overall performance of the decoders. The area under the ROC curve (AUC ROC) of the ensemble model is 0.963. AUC ROC of the base models are 0.881, 0.893, 0.920, 0.925, and 0.924. The models were trained on a dataset with 100 replicated per class.

(D) Schematic of the majority voting algorithm. Instead of using only one pattern for communication, the sender would generate and send out multiple patterns representing the same message. Due to the randomness in the patterning process, these patterns appear similar but differ in detail. The recipient would use a trained decoder to decode each pattern and obtain the corresponding predictions. The most popular prediction will be used as the final prediction.

prediction capabilities. The ensemble model was then used for final decoding (Figure 4A, see "ensemble learning and uncertainty estimation" in methods). For patterns generated with moderate growth noise, the prediction performance of the ensemble decoder excels that of the base models for up to 22% in accuracy (Figure 4B). Receiver operating characteristic (ROC) curves and confusion matrices also show significant improvement with ensemble model (Figures 4C, S8, and S9). As expected, the ensemble model generally outperforms the base ones when intermediate data are available but demonstrates marginal improvement with adequate or scarce data. This is expected because when intermediate data are available, the individual base models are diversified due to random initialization. However, when adequate data are available, each base model individually decodes with high accuracy, leaving little room for improvement. Conversely, when data are scarce, the base decoders barely learn such that integrating their results provides little insight. This final aspect implies encoding security against minor data leakage. In addition, considerable improvement can be achieved with a simple logistic regression model, and more base models leads to better ensemble performance (Figure S10). In addition to stacking, we have also shown that majority voting can improve the decoding accuracy (Figures 4D and S11). Multiple patterns corresponding to the same character were decoded using the same CNN, and the most voted prediction was used as the final prediction.

Ensemble learning not only improves the decoding accuracy, but also sheds light on the prediction uncertainty. According to Lakshminarayanan et al., the base models trained with random initialization explore the entirely different modes of function space,[34] thus their independent predictions can be used to estimate well-calibrated uncertainty.[35] We adopted this notion and estimated decoding uncertainty through multiple metrics, including log likelihood, mean square error (MSE), top 1 and top 5 errors (see "ensemble learning and uncertainty estimation" in methods). A higher metric value indicates larger uncertainty or lower confidence. As expected, the uncertainty reduces as more training data are available (Figure S12). Having more base models does not necessarily reduce the uncertainty (Table S2).

Distributed encoding of English in Emorfi

Our distributed encoding-decoding platform is scalable for practical applications. We constructed 100 sets of patterns

CellPress
OPEN ACCESS

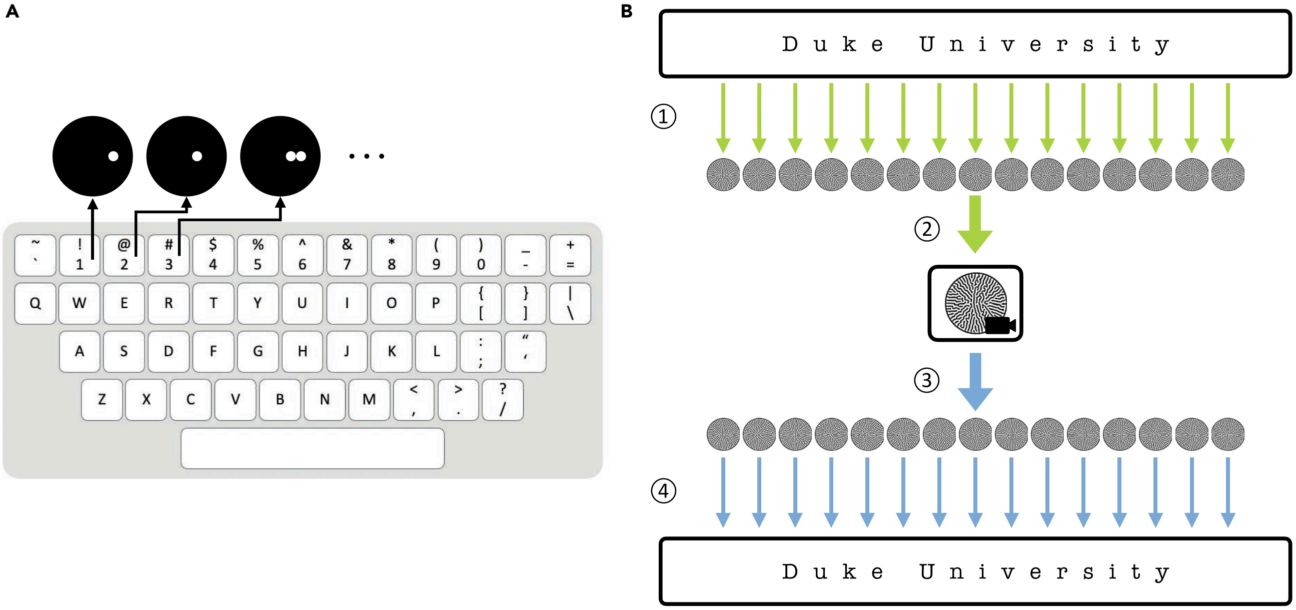

Figure 5. Encoding text in Emorfi

(A) Each of the 100 printable ASCII characters is represented by a unique initial configuration. Ninety-five of them are shown on the keyboard, and five other printable whitespace characters (tab, linefeed, return, vertical tab, and formfeed) are not shown here. In the training set, each character maps to 1,000 patterns. The collection of patterns, as well as subsequent ones to be generated, constitutes Emorfi.

(B) A piece of text could be encoded as a video and decoded using the ensemble method. ① Each character in the text is translated to a corresponding pattern. ② The images are arranged in order and assembled into a video that can be used for communication. ③ To decode, each frame is retrieved from the video. ④ The patterns are decoded sequentially, representing the decoded text.

to encode all printable ASCII characters including English letters in upper and lower cases, digits, punctuations, and whitespaces (Figure 5A; Appendix A). A 7-bit seeding array was used to create the training dataset, in which 100 of the unique initial configurations corresponded to the printable characters. Each of the initial configurations was then used to generate 1,000 patterns. We term this collection of patterns Emorfi, which represents a new, digitally generated coding scheme. When encoding text, each character is represented by one or multiple newly generated patterns with the same setup, and the patterns are then arranged to assemble a video (Figure 5B).

By doing so, all standard English text can be encoded in Emorfi and decoded back. For instance, we encoded the public speech "I have a dream" by Martin Luther King Jr. containing 8,869 individual characters as a video (Video S1). Accommodating majority voting, each character was represented by five different patterns, and 99.8% of the text can be correctly decoded (Appendix B). The same approach was also used to encode the poem "Auguries of Innocence" by William Blake as a video (Video S2), and 99.6% of the text was correctly decoded (Appendix C). In another example, using a 5-bit seeding array, we encoded the GFP protein sequence (238 amino acids) as a video (Video S3) and 100% was correctly decoded (Appendix D). In these real-world use cases, attackers with limited access to the training data cannot decode successfully. For example, having access to 10 patterns per class would only lead to decoding 1.3% of "Auguries of Innocence," which is much lower than using a properly trained decoder.

DISCUSSION

Our encoding and decoding framework is applicable to diverse dynamics systems, as long as they have three key properties: (1) an approximately convergent mapping between initial input and output, (2) complex output signals, and (3) the output patterns are difficult to distinguish to the naked eye. While past studies have explored the possibility of using chaos to encode information and to provide security,[36–38] unavoidable noise and error in numerical simulation (e.g., finite precision computing) or transmission (e.g., channel noise) can alter the output despite these systems being deterministic. In contrast, the convergent nature of our system ensures patterns that originate from the same initial configurations share common features (recognizable by a trained NN) despite small variances. Although noise is often considered undesirable in biological studies—such as masking ground truth[39–41] or disrupting interactions between components[42,43]—we take advantage of the variance in our system to ensure information security and to authenticate each pattern. These features distinguish our methods from other biology-based information encoding, encryption, or storage methods, such as DNA sequences,[44,45] DNA origami,[46] and arrays of microbial colony,[47] which mostly rely on one-to-one mapping between the information to encode and the encoded format.

Our proposed criteria together contribute to the sufficient encoding capacity and tunable information security of our platform. Many systems satisfy these criteria. With appropriate parameterization and boundary conditions, many reaction-diffusion models exhibit considerable robustness in output patterns and

sensitivity to initial conditions.[48,49] In addition to the example we demonstrated (Figures 2, S4, and S5), many CA models with asynchrony update rules also show convergence.[50,51] Biological systems, such as biofilm morphology, butterfly wing scale pattern, and human fingerprint, have also evolved to exhibit common features but vary in detail. Their convergent nature results from the rich multiscale, multidimensional interactions between different system components, such as chemical reactions and diffusion, gene circuits, and cell-cell interactions.[52–56] Our work can motivate future studies of utilizing other types of dynamical system outputs or implementing information encoding using controllable experimental patterns. Similar to the computational examples, the methods of selecting a suitable system, and balancing encoding capacity and security are also applicable for experimental systems.

However, our work does bring up a fundamental question: given a dynamical system with stochasticity, how do we know the dynamics are convergent enough while the output signals from different initial conditions are also distinguishable? We suspect that the question has to be addressed empirically for each specific system. In ours, each initial configuration generates an ensemble of output patterns following a distribution (visualized using t-SNE in Figure S13). It is difficult to determine this distribution by solely inspecting the pattern-generation model, even if parameters and noise magnitudes are known. However, whether each distribution corresponding to an input can be distinguished from another distribution arising from another input is established by ML. In essence, the trained CNN provides an empirical estimate on the extent by which the pattern generation is convergent. To this end, our work has implications for quantifying the convergence for a dynamical system by using ML.

As we have demonstrated with Emorfi, the pattern-based encoding-decoding platform is scalable and generalizable for information in various formats. We envision that the platform could be extended to other languages, such as alphabetic languages with different letters or diacritics (e.g., French, Hebrew) and logographic scripts consisting of thousands of characters (e.g., Chinese, Japanese). It could also be applicable for communicating science and protecting intellectual properties by incorporating Greek alphabet, mathematical symbols, nucleic acid bases, etc. In addition, one may increase the information density from one-character-per-pattern to multiple-characters-per-pattern by using more complex initial conditions, or improve the information efficiency by choosing more convergent systems. The encoding speed could be accelerated by using a faster pattern generator.

EXPERIMENTAL PROCEDURES

Resource availability

Lead contact

Further information and requests for resources should be directed to and will be fulfilled by the lead contact, Lingchong You (you@duke.edu).

Materials availability

This study did not utilize any materials aside from the code noted below and did not generate new unique reagents.

Data and code availability

The mathematical simulation and machine learning codes used in this study are available on GitHub: https://github.com/youlab/Information_encoding. The platform for encoding text in the format of video is available at https://www.patternencoder.com/.

Mathematical modeling

The simple colony pattern-generation model accounts for several driving forces. In particular, it uses a kernel-based method to capture the high-level positive (expansion) and negative (inhibition) effects on patterning, regardless of the specific mechanism. The model is formulated as the following equations:

$$N_{t+1}(\bar{x},\bar{y}) = \int\int K(d_{x,y})\, N_t(\bar{x},\bar{y})dxdy$$

$$K(d_{x,y}) = b\,2^{-\left(\frac{d}{d1}\right)^{h_1}} - 2^{-\left(\frac{d}{d2}\right)^{h_2}}.$$

Here, N is the colonization of the bacteria over the growing medium, K is the growth kernel that is the addition of the expansion and (negative) repulsion kernels. b is the relative magnitude of expansion to repulsion, d_1 and d_2 are the distances that characterize half of the maximum effect of expansion and repulsion respectively. $d_{x,y}$ is the distance of a position (x, y) to $(\bar{x}, \bar{y})$. We used $d_1/d_2 = 0.4$, $h_1 = 1{,}000$, $h_2 = 2{,}000$, and $b = 6.5$ as the default parameter values unless otherwise mentioned. This parameter set generates complex branching patterns.

To adapt the published model for our study, we made several modifications. First, we implemented various seeding configuration, such as the spot seeding arrangement for encoding binary representations of characters (Figure 1B). The size and spacing of the spots were subjected to modulation. As the default setting, we used spacing = 15 and spot radius = 5. Second, we implemented white Gaussian noise with varying signal-to-noise (SNR) ratios to the growth kernel at each time step. The noise (σ) mimics the heterogeneity and small perturbations in growth. Thus, the kernel equation becomes:

$$K(d_{x,y}) = b\,2^{-\left(\frac{d}{d1}\right)^{h_1}} - 2^{-\left(\frac{d}{d2}\right)^{h_2}} + \sigma.$$

We also implemented uneven cell seeding by assigning random intensities drawn from a truncated Gaussian distribution (mean = 0.5, deviation varies) to the pixels within the spot configurations. Both noise sources contribute to the variation in patterns given the same model parameters and initial configurations. As default, we used random seeding without growth noise.

The model was implemented in MATLAB 2017b and solved numerically. The simulation terminates once the colony stops growing. The simulation outputs an 8-bit, 451 × 451 pixel grayscale image. Except for the encryption experiments, the patterns were formed on a circular growth domain of a diameter of 451 pixels.

To generate different patterns, we modulated the relative acting distance ($d1/d2$) and magnitude of colony expansion versus repulsion processes (b). Large relative distance and magnitude (i.e., higher colony expansion) result in thick branches, whereas small relative distance and magnitude (i.e., higher repulsion) result in thin, sparse branches. In extreme cases, these conditions can result in large disks or small circular colonies, respectively. When these two forces are intermediate and comparable, the system generates branching colonies.

CNN training

For CNN training, we numerically simulated datasets with equal numbers of replicates for each encoding character. For evaluation, test datasets made of 100 replicates per class were used. The pattern images were rescaled to 80 × 80 pixels before training or testing.

The CNN (Figure S2) and the ensemble model (Figure 4A) were implemented in Python 3, TensorFlow 1.15.2, and Keras 2.4.0. The CNN uses pattern images as inputs and outputs N features, where N is the dictionary size (i.e., number of characters in a dictionary). It consists of two convolutions, each followed by max pooling and rectified linear unit (ReLU). Then their output is passed onto two fully connected layers, followed by ReLU and softmax, respectively. Here, the softmax function turns it into categorical probabilities. For training, we used Glorot normal initializer, categorical cross entropy loss, and Adam optimization algorithm with learning rate subject to tuning. Keras early

stopping function was also implemented to stop the training once the loss metric stopped improving. We carried out hyperparameter tuning (including learning rate, batch size, early stopping patience, and delta) to obtain the best performing models for analysis. The data generation and training were conducted on Duke Compute Cluster and Google Cloud Platform.

Encryption dataset generation

The geometry of the growth domain impacts the growth and pattern formation through exerting a negative effect on the colony in the vicinity of the boundary, such that the colony does not reach the edge. The plate influence is formulated as:

$$I = -k\,2^{-\varepsilon\frac{d}{R}}.$$

The model is:

$$N_{t+1}(\overline{x},\overline{y}) = \int\int\left(K\left(d_{x,y}\right) + I\left(d_{x,y}\right)\right)N_t(\overline{x},\overline{y})dxdy.$$

Here, d is the Euclidean distance of a position (x, y) in the space to the boundary, and $k = 1{,}000$. R is the plate radius. For irregular domains, contour lines are drawn to determine $\frac{d}{R}$, where R is the value of the highest contour line. ε regulates the shape of the impact function. We deducted the influence from the colony after each discrete time step. For the purpose of encryption, we maximized the influence of the geometry by modulating ε, such that the negative plate impact reached as far as the center of the patterns. We used $\varepsilon = 1$ for generating the encryption datasets, and 2,000 for any other dataset.

When using the shape of the growing medium as the secret key, we simulated the colony patterns on circular-, diamond-, square-, and equilateral triangular-shaped domains. The area of each geometry was kept the same to compare the effect of the geometry. We removed the information of growth domain shape by cropping out a smaller, circular area at the center of each pattern, and only the processed pattern images were used for CNN training.

ENSEMBLE LEARNING AND UNCERTAINTY ESTIMATION

The training of ensemble model was carried out in two steps. First, we trained several base CNN models using the same protocol described in "CNN training." Their probabilistic predictions on the training set were then linearly combined to constitute a new dataset. Next, we used the new dataset to train an ensemble model from scratch. We tested several ensemble model architectures, including logistic regression and feedforward neural networks with different numbers of hidden layers and nodes. In the ensemble model, we used ReLU activation function for the input and hidden layers and passed the model output into softmax function to turn it into categorical probabilities. For its training, we used Glorot uniform initializer, categorical cross entropy loss, and Adam optimization algorithm with learning rate = 0.0001. Keras early stopping was used to stop the training once the loss metric stopped improving. The patience was 5 and the minimum change was 0.0001. We evaluated the model performance on a balanced dataset of 100 datapoints per class through metrics such as precision, recall, ROC, AUC ROC using scikit-learn (0.22.2).

We evaluated the prediction uncertainty based on the output of base models. We used common metrics, such as log likelihood, MSE, and top 1 and top 5 errors, for estimating the uncertainty. Specifically, the log likelihood is $-M^{-1}\sum_{j=1}^{M}\sum_{i=1}^{N} y_{ij}\ \log(p_{ij})$ and the MSE is $M^{-1}N^{-1}\sum_{j=1}^{M}\sum_{i=1}^{N}(y_{ij} - p_{ij})^2$. For the ith data point, y_{ij} is the true label for class j (1 if the data point belongs to class j, otherwise 0), p_{ij} is the predicted probabilities for class j. M indicates the total number of data points, N indicates the dictionary size, and top 1 and top 5 indicate the fraction of data points whose correct label is not among their top 1 or 5 probable predictions, respectively.

SUPPLEMENTAL INFORMATION

Supplemental information can be found online at https://doi.org/10.1016/j.patter.2022.100590.

ACKNOWLEDGMENTS

We thank Thomas Witelski, Sayan Mukherjee, and Teng Wang for the helpful discussion and comments on the manuscript. We thank Duke Compute Cluster for assistance with high-throughput computation. This study was partially supported by the National Science Foundation (MCB-1937259), the Office of Naval Research (N00014-20-1-2121), the David and Lucile Packard Foundation, and the Google Cloud Research Credits program.

AUTHOR CONTRIBUTIONS

J.L. and L.Y. conceived the research and wrote the manuscript. J.L. designed and performed mathematical modeling and machine learning training and carried out data analysis. N.L. assisted with modeling. L.Y., R.T., N.L., and Y.H. assisted with results interpretation. J.L., M.K., Y.B., N.M., S.T., and A.Z. developed the website. R.T., N.L., Y.H., S.W., and N.G. contributed to manuscript revisions.

DECLARATION OF INTERESTS

The authors declare no competing interests.

Received: June 10, 2022
Revised: July 29, 2022
Accepted: August 24, 2022
Published: September 23, 2022

REFERENCES

1. Codd, E.F. (2014). Cellular Automata (Academic Press).
2. Wolfram, S. (1985). Cryptography with Cellular Automata (Springer), pp. 429–432.
3. Nandi, S., Kar, B.K., and Pal Chaudhuri, P. (1994). Theory and applications of cellular automata in cryptography. IEEE Trans. Comput. *43*, 1346–1357.
4. Rhouma, R., Meherzi, S., and Belghith, S. (2009). OCML-based colour image encryption. Chaos, Solit. Fractals *40*, 309–318.
5. Behnia, S., Akhshani, A., Ahadpour, S., Mahmodi, H., and Akhavan, A. (2007). A fast chaotic encryption scheme based on piecewise nonlinear chaotic maps. Phys. Lett. *366*, 391–396.
6. Wang, X., and Jin, C. (2012). Image encryption using game of life permutation and PWLCM chaotic system. Opt Commun. *285*, 412–417.
7. Xiang, T., Liao, X., Tang, G., Chen, Y., and Wong, K.-w. (2006). A novel block cryptosystem based on iterating a chaotic map. Phys. Lett. *349*, 109–115.
8. Wang, X., and Luan, D. (2013). A novel image encryption algorithm using chaos and reversible cellular automata. Commun. Nonlinear Sci. Numer. Simulat. *18*, 3075–3085.
9. Martinez, G.J. (2013). A note on elementary cellular automata classification. Preprint at arXiv.
10. Schüle, M., and Stoop, R. (2012). A full computation-relevant topological dynamics classification of elementary cellular automata. Chaos *22*, 043143.

11. Feistel, H. (1973). Cryptography and computer privacy. Sci. Am. *228*, 15–23.
12. Lafe, O. (1997). Data compression and encryption using cellular automata transforms. Eng. Appl. Artif. Intell. *10*, 581–591.
13. Langton, C.G. (1990). Computation at the edge of chaos: phase transitions and emergent computation. Phys. Nonlinear Phenom. *42*, 12–37.
14. Bertram, M., Beta, C., Pollmann, M., Mikhailov, A.S., Rotermund, H.H., and Ertl, G. (2003). Pattern formation on the edge of chaos: experiments with CO oxidation on a Pt (110) surface under global delayed feedback. Phys. Rev. E Stat. Nonlin. Soft Matter Phys. *67*, 036208.
15. Dahmen, D., Grün, S., Diesmann, M., and Helias, M. (2019). Second type of criticality in the brain uncovers rich multiple-neuron dynamics. Proc. Natl. Acad. Sci. USA *116*, 13051–13060.
16. Julkowska, D., Obuchowski, M., Holland, I.B., and Séror, S.J. (2004). Branched swarming patterns on a synthetic medium formed by wild-type Bacillus subtilis strain 3610: detection of different cellular morphologies and constellations of cells as the complex architecture develops. Microbiology *150*, 1839–1849.
17. Granek, J.A., and Magwene, P.M. (2010). Environmental and genetic determinants of colony morphology in yeast. PLoS Genet. *6*, e1000823.
18. Mayer-Hamblett, N., Rosenfeld, M., Gibson, R.L., Ramsey, B.W., Kulasekara, H.D., Retsch-Bogart, G.Z., Morgan, W., Wolter, D.J., Pope, C.E., Houston, L.S., et al. (2014). Pseudomonas aeruginosa in vitro phenotypes distinguish cystic fibrosis infection stages and outcomes. Am. J. Respir. Crit. Care Med. *190*, 289–297.
19. Tabor, J.J., Salis, H.M., Simpson, Z.B., Chevalier, A.A., Levskaya, A., Marcotte, E.M., Voigt, C.A., and Ellington, A.D. (2009). A synthetic genetic edge detection program. Cell *137*, 1272–1281.
20. Deng, P., de Vargas Roditi, L., Van Ditmarsch, D., and Xavier, J.B. (2014). The ecological basis of morphogenesis: branching patterns in swarming colonies of bacteria. New J. Phys. *16*, 015006.
21. Krizhevsky, A., Sutskever, I., and Hinton, G.E. (2017). Imagenet classification with deep convolutional neural networks. Commun. ACM *60*, 84–90.
22. Szegedy, C., Liu, W., Jia, Y., Sermanet, P., Reed, S., Anguelov, D., Erhan, D., Vanhoucke, V., and Rabinovich, A. (2015). Going Deeper with Convolutions, pp. 1–9.
23. Lawrence, S., Giles, C.L., Tsoi, A.C., and Back, A.D. (1997). Face recognition: a convolutional neural-network approach. IEEE Trans. Neural Netw. *8*, 98–113.
24. Bard, J.B. (1981). A model for generating aspects of zebra and other mammalian coat patterns. J. Theor. Biol. *93*, 363–385.
25. Zhang, C., Li, B., Tang, J.-Y., Wang, X.-L., Qin, Z., and Feng, X.-Q. (2017). Experimental and theoretical studies on the morphogenesis of bacterial biofilms. Soft Matter *13*, 7389–7397.
26. Zhu, J., Zhang, Y.-T., Newman, S.A., and Alber, M. (2009). Application of discontinuous Galerkin methods for reaction-diffusion systems in developmental biology. J. Sci. Comput. *40*, 391–418.
27. Tan, A.C., and Gilbert, D. (2003). Ensemble Machine Learning on Gene Expression Data for Cancer Classification.
28. Zhang, C., and Ma, Y. (2012). Ensemble Machine Learning: Methods and Applications (Springer).
29. Ertosun, M.G., and Rubin, D.L. (2015). Automated Grading of Gliomas Using Deep Learning in Digital Pathology Images: A Modular Approach with Ensemble of Convolutional Neural Networks (American Medical Informatics Association), p. 1899.
30. Wolpert, D.H. (1992). Stacked generalization. Neural Network. *5*, 241–259.
31. Ma, Z., Wang, P., Gao, Z., Wang, R., and Khalighi, K. (2018). Ensemble of machine learning algorithms using the stacked generalization approach to estimate the warfarin dose. PLoS One *13*, e0205872.
32. Ting, K.M., and Witten, I.H. (1999). Issues in stacked generalization. J. Artif. Intell. Res. *10*, 271–289.
33. Chowdhury, A.S., Khaledian, E., and Broschat, S.L. (2019). Capreomycin resistance prediction in two species of Mycobacterium using a stacked ensemble method. J. Appl. Microbiol. *127*, 1656–1664.
34. Fort, S., Hu, H., and Lakshminarayanan, B. (2019). Deep ensembles: a loss landscape perspective. Preprint at arXiv.
35. Lakshminarayanan, B., Pritzel, A., and Blundell, C. (2016). Simple and scalable predictive uncertainty estimation using deep ensembles. Preprint at arXiv.
36. Lai, Y.-C. (2000). Encoding digital information using transient chaos. Int. J. Bifurcation Chaos *10*, 787–795.
37. Hayes, S., Grebogi, C., and Ott, E. (1993). Communicating with chaos. Phys. Rev. Lett. *70*, 3031–3034.
38. Parlitz, U., Kocarev, L., Stojanovski, T., and Preckel, H. (1996). Encoding messages using chaotic synchronization. Phys. Rev. E Stat. Phys. Plasmas Fluids Relat. Interdiscip. Topics *53*, 4351–4361.
39. Gregor, T., Tank, D.W., Wieschaus, E.F., and Bialek, W. (2007). Probing the limits to positional information. Cell *130*, 153–164.
40. Kontogeorgaki, S., Sánchez-García, R.J., Ewing, R.M., Zygalakis, K.C., and MacArthur, B.D. (2017). Noise-processing by signaling networks. Sci. Rep. *7*, 532–539.
41. Horikawa, K., Ishimatsu, K., Yoshimoto, E., Kondo, S., and Takeda, H. (2006). Noise-resistant and synchronized oscillation of the segmentation clock. Nature *441*, 719–723.
42. Potvin-Trottier, L., Lord, N.D., Vinnicombe, G., and Paulsson, J. (2016). Synchronous long-term oscillations in a synthetic gene circuit. Nature *538*, 514–517.
43. Lestas, I., Vinnicombe, G., and Paulsson, J. (2010). Fundamental limits on the suppression of molecular fluctuations. Nature *467*, 174–178.
44. Ceze, L., Nivala, J., and Strauss, K. (2019). Molecular digital data storage using DNA. Nat. Rev. Genet. *20*, 456–466.
45. Shipman, S.L., Nivala, J., Macklis, J.D., and Church, G.M. (2017). CRISPR–Cas encoding of a digital movie into the genomes of a population of living bacteria. Nature *547*, 345–349.
46. Zhang, Y., Wang, F., Chao, J., Xie, M., Liu, H., Pan, M., Kopperger, E., Liu, X., Li, Q., Shi, J., et al. (2019). DNA origami cryptography for secure communication. Nat. Commun. *10*, 5469–5478.
47. Palacios, M.A., Benito-Peña, E., Manesse, M., Mazzeo, A.D., LaFratta, C.N., Whitesides, G.M., and Walt, D.R. (2011). InfoBiology by printed arrays of microorganism colonies for timed and on-demand release of messages. Proc. Natl. Acad. Sci. USA *108*, 16510–16514.
48. Murray, J.D. (1981). A pre-pattern formation mechanism for animal coat markings. J. Theor. Biol. *88*, 161–199.
49. Maini, P.K., Woolley, T.E., Baker, R.E., Gaffney, E.A., and Lee, S.S. (2012). Turing's model for biological pattern formation and the robustness problem. Interface Focus *2*, 487–496.
50. Bersini, H., and Detours, V. (1994). Asynchrony Induces Stability in Cellular Automata Based Models (MIT Press), pp. 382–387.
51. Fatès, N.A., and Morvan, M. (2004). An experimental study of robustness to asynchronism for elementary cellular automata. Preprint at arXiv.
52. Adamatzky, A., Costello, B.D.L., and Asai, T. (2005). Reaction-diffusion Computers (Elsevier).
53. Adleman, L.M. (1994). Molecular computation of solutions to combinatorial problems. Science *266*, 1021–1024.
54. Tamsir, A., Tabor, J.J., and Voigt, C.A. (2011). Robust multicellular computing using genetically encoded NOR gates and chemical 'wires. Nature *469*, 212–215.
55. Castro, L.N.d., and Timmis, J.I. (2003). Artificial immune systems as a novel soft computing paradigm. Soft Comput. A Fusion Found. Methodol. Appl. *7*, 526–544.
56. Payne, S., Li, B., Cao, Y., Schaeffer, D., Ryser, M.D., and You, L. (2013). Temporal control of self-organized pattern formation without morphogen gradients in bacteria. Mol. Syst. Biol. *9*, 697.

Patterns, Volume *3*

Supplemental information

Distributed information encoding and decoding

using self-organized spatial patterns

Jia Lu, Ryan Tsoi, Nan Luo, Yuanchi Ha, Shangying Wang, Minjun Kwak, Yasa Baig, Nicole Moiseyev, Shari Tian, Alison Zhang, Neil Zhenqiang Gong, and Lingchong You

Supplementary Information

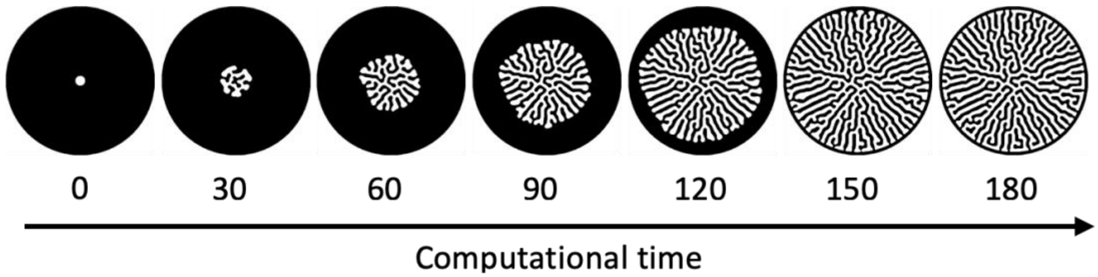

Fig. S1. Time series of branching pattern development.
An example of colony growth starting from a single spot configuration at the center of a circular growth domain. The cells are unevenly seeded within the initial configuration. As the colony grows, it develops into an intricate branching pattern and stops growing at around t = 180. The simulation parameters are the default: seeding spot radius = 5, $d_1/d_2 = 0.4$, $h_1 = 1000, h_2 = 2000, b = 6.5$, $\epsilon = 2000$, there was not growth noise (See "Methods").

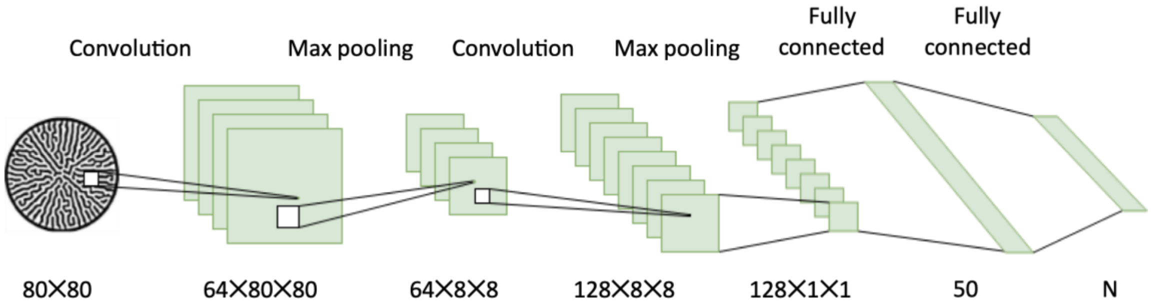

Fig. S2. Schematic of CNN decoder architecture.
The CNN takes an 80 × 80 greyscale image as input, and outputs a N dimensional feature, where N is the number of characters in a dictionary.

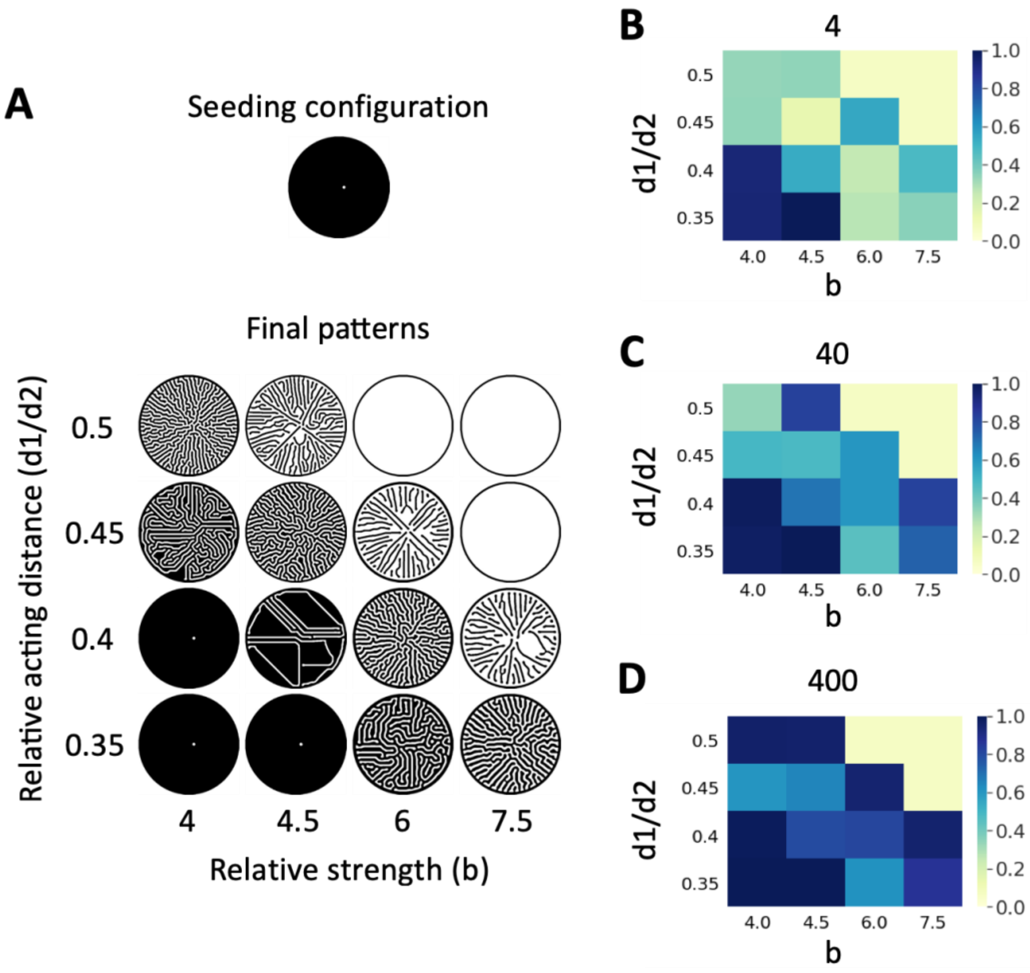

Fig. S3. Patterning dynamics impact the tradeoff among encoding capacity, security, and decoding reliability.

A. Starting from the same initial seeding configuration (and the same seeding noise), cells grow into diverse final patterns under different growth dynamics. Here, the growth dynamic is defined by two parameters: the relative strength and the relative acting distance of colony expansion and repulsion. The simulation is terminated when the growth stops. We can coarsely classify the patterns into three groups: the trivial (lower left corner), the disk-like (top right corner) and the branching patterns (the ones along the diagonal).

B – D. Decoding accuracy of different dynamics when scarce (B), intermediate (C), and adequate (D) training data are available (4, 40, and 400 replicates per class respectively). The trivial patterns always have high decoding capacity, whereas the disk-like patterns always have low decoding capacity. For branching patterns, the decoding accuracy increases drastically if more data become available.

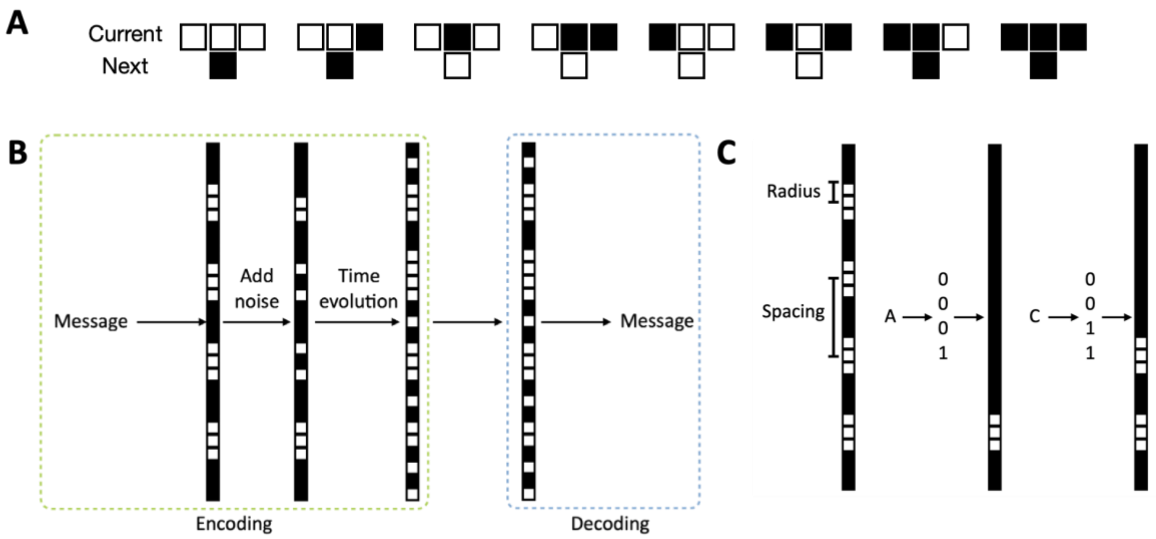

Fig. S4. Encoding and decoding using elementary cellular automata (ECA) with rule 60.
A. Rule 60 of ECA. Color indicates cell status: black – 0; white – 1. For the specific "Current" states, the sequence of the "Next" states represents the number 60 in a binary format (00111100).
B. Encoding-decoding scheme using one dimensional ECA. To encode, a message is first converted into a one-dimensional seeding array and then noise is added to it. The sequence then evolves into a final pattern following rule 60. A trained feedforward neural network is used to decode the pattern.
C. Predefined braille-like cell seeding arrangement and examples of encoded letters.

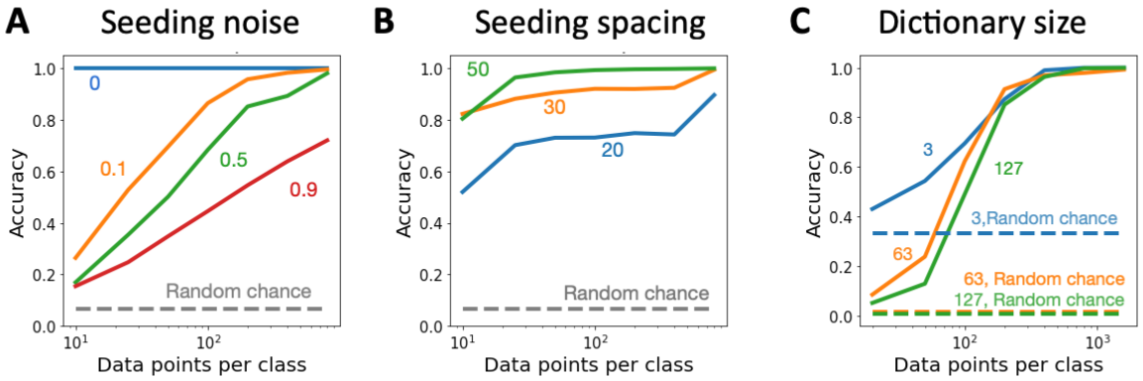

Fig. S5. Tradeoff between encoding capacity, security, and decoding reliability of the CA model. We varied parameters including: A. level of noise in time evolution (increasing value indicates higher noise), B. spacing between spots (time step = 600), and C. number of characters in encoding setup. The decoding accuracy generally increases as the number of replicates per class increases, and it significantly exceeds the corresponding accuracy by random guessing. Increasing complexity such as using larger growth noise or smaller spacing would require more data to reach the same accuracy. Larger dictionary size does not lead to sufficiently distinguishable training performance profile, it indicates every system may have different capacity and require tuning.

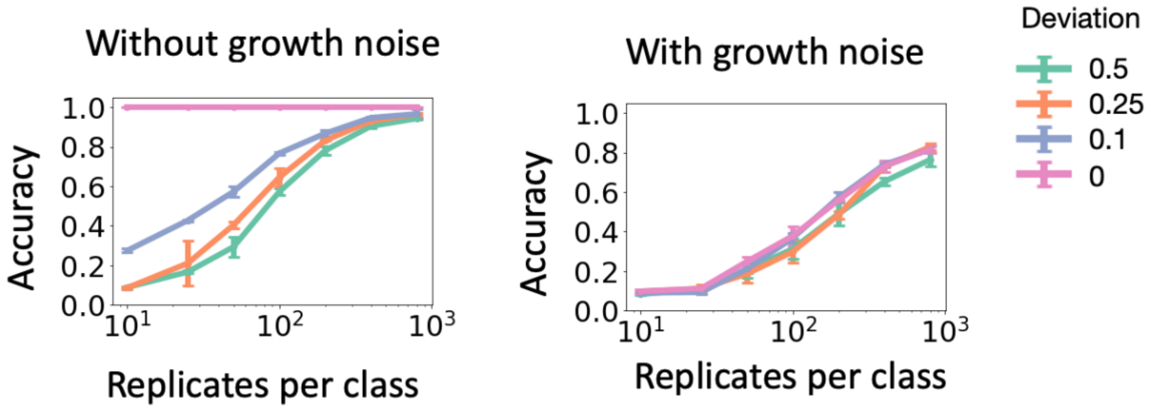

Fig. S6. Impact of seeding noise on the tradeoff among encoding capacity, security, and decoding reliability.

The seeding noise is implemented by assigning each pixel within the seeding configuration a random value. These values are drawn from a truncated Gaussian distribution with a mean of 0.5 and a given deviation (0, 0.1, 0.25 and 0.5 respectively). Larger deviation results in larger seeding noise. In the absence of growth noise, larger seeding noise leads to more challenging decoding, which is indicated by the increasing required training data. However, this impact is minor and can be obscured by a gentle growth noise (SNR = 10, see “Methods” for more details). Data are represented as mean ± standard deviation.

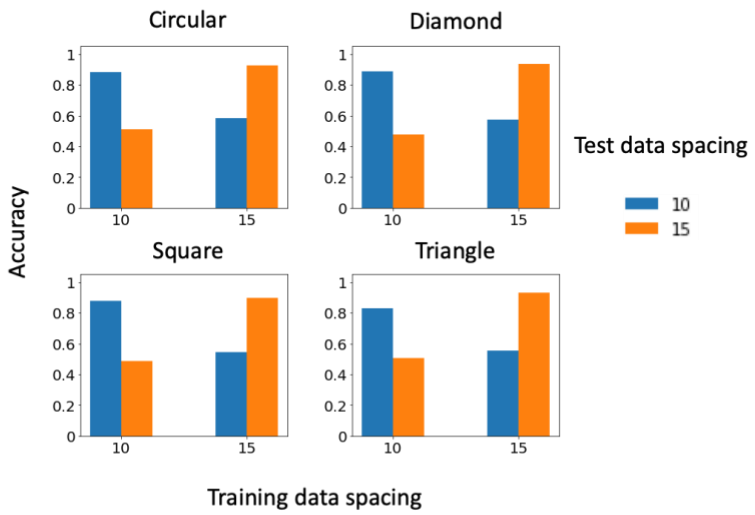

Fig. S7. Spacing as the encryption secret key.
Spacing distance = 10 and 15 were used respectively as the secret key to generate the training patterns. On growing media of all shapes, only models trained with the correct dataset can decode the patterns at significantly higher accuracy. The results indicate that spacing is a feasible choice for the secret key.

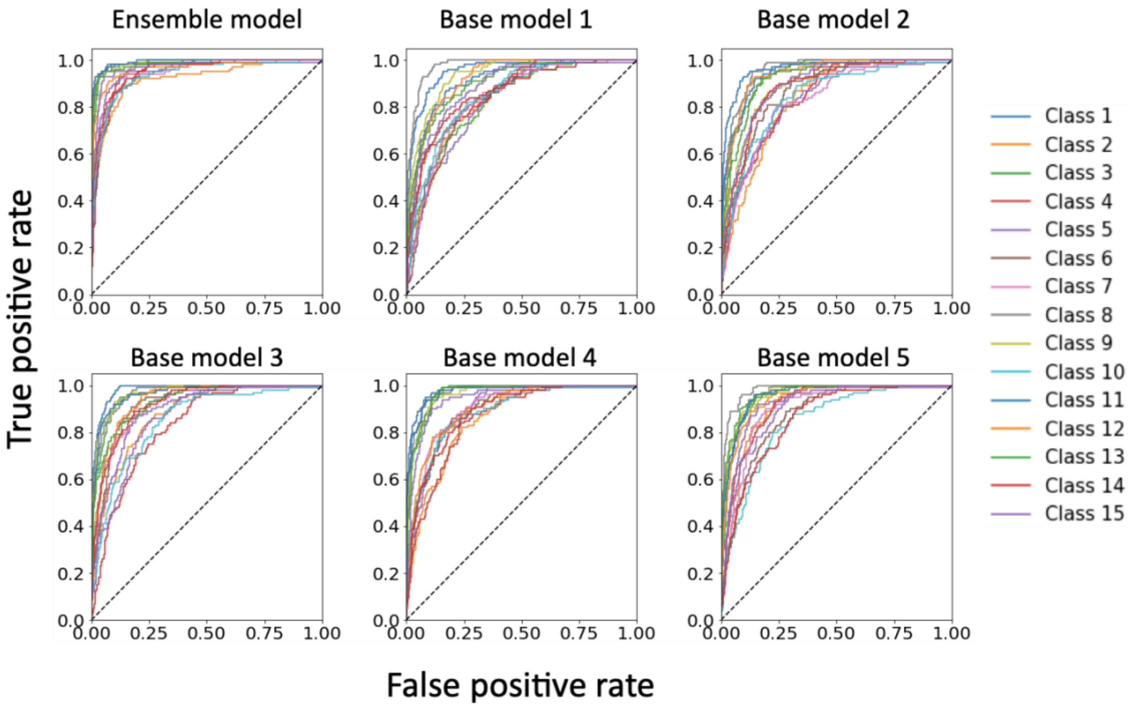

Fig. S8. Multiclass ROC of ensemble and base models.
We used 5 base CNN models to train a LR ensemble model. The training data were patterns generated using moderate growth noise (SNR = 3.5) and the dataset was composed of 100 replicates per class. Among base models, the ROC curves vary drastically. On the contrary, the variation reduces for the ensemble model, and the curves shift towards the upper left corner indicating significant performance improvement.

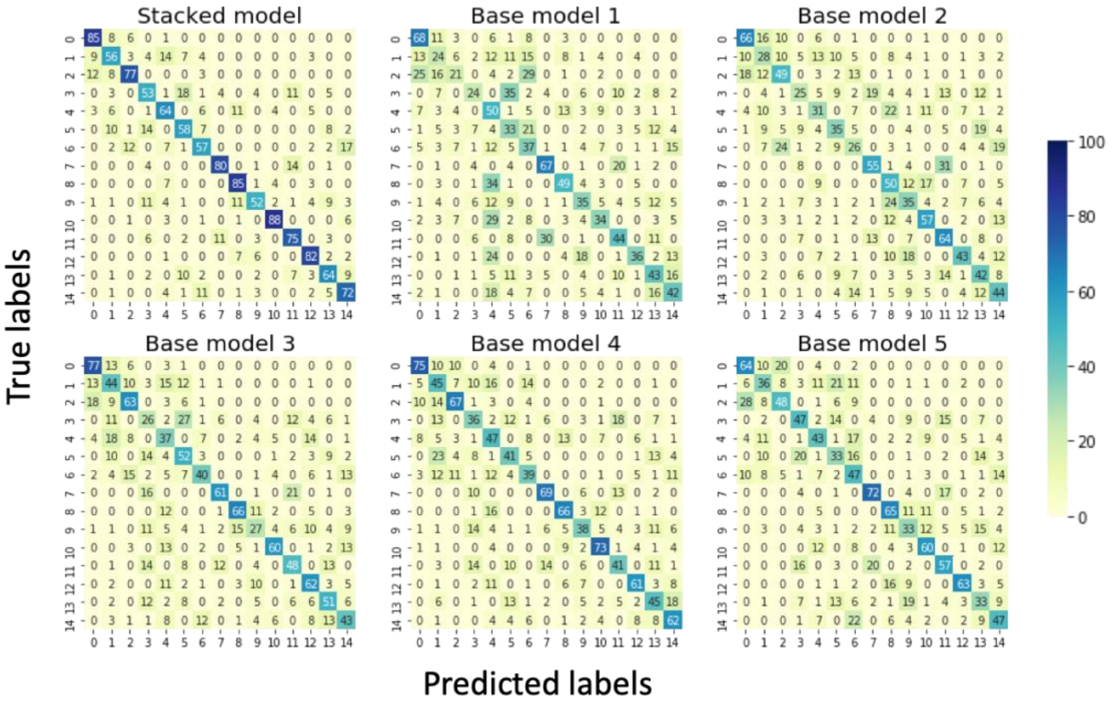

Fig. S9. Confusion matrix of ensemble and base models.

The base and ensemble models were trained on the same dataset in the same way as in Figure S6. Overall, the chance of misclassification reduces considerably for the ensemble model in comparison to individual base models.

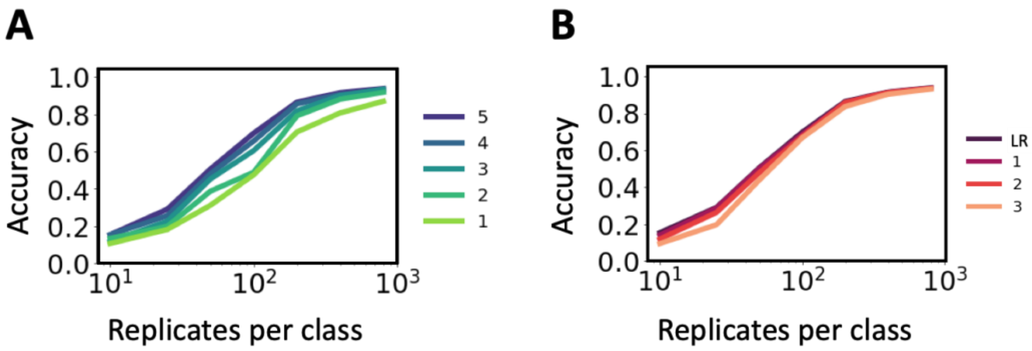

Fig. S10. The impact of the number of base models and ensemble model architecture on ensemble prediction.

A. Colors indicate different number of base models. The ensemble accuracy improves as more base models are used for constructing a LR ensemble model, and the amount of increase depends on the dataset size. The accuracy would eventually saturate when sufficiently many base models are used.
B. Logistic regression (LR) as the ensemble model outperforms feedforward neural network (FNN) with either 1, 2 or 3 hidden layers. Here, we kept the input and output layers the same for all models, and the number of hidden nodes for the FNNs are (40), (60, 30) and (60, 45, 25) respectively. LR performs slightly better or as good as FNNs.

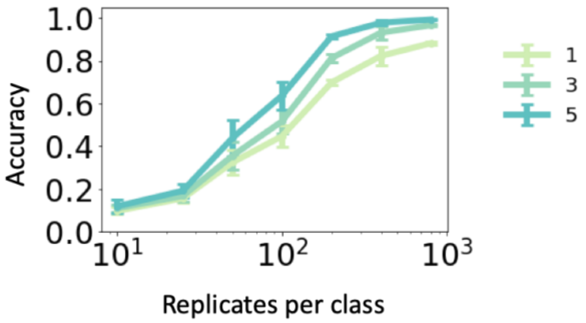

Fig. S11. Majority voting improves decoding accuracy.
Decoding accuracy of using 1, 3 and 5 patterns under the majority voting scheme. Using more patterns can significantly improve the performance, especially when a large dataset has been used for training. Data are represented as mean ± standard deviation.

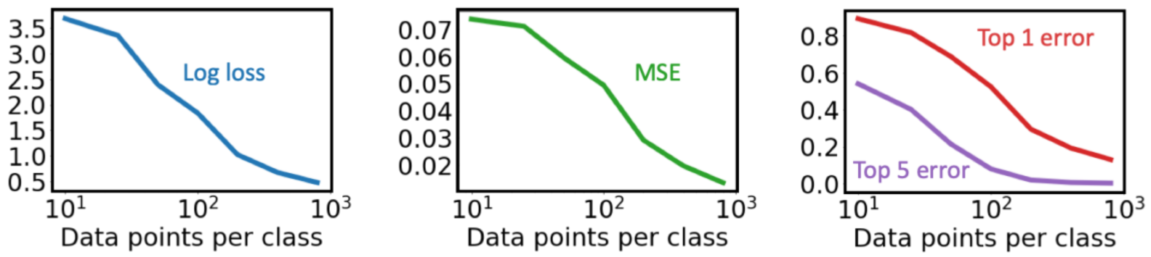

Fig. S12. Uncertainty estimation using deep ensemble.
5 base models trained using random initialization were used to calculate the selected metrics. Higher value indicates greater uncertainty. Having more training data lowers the prediction uncertainty.

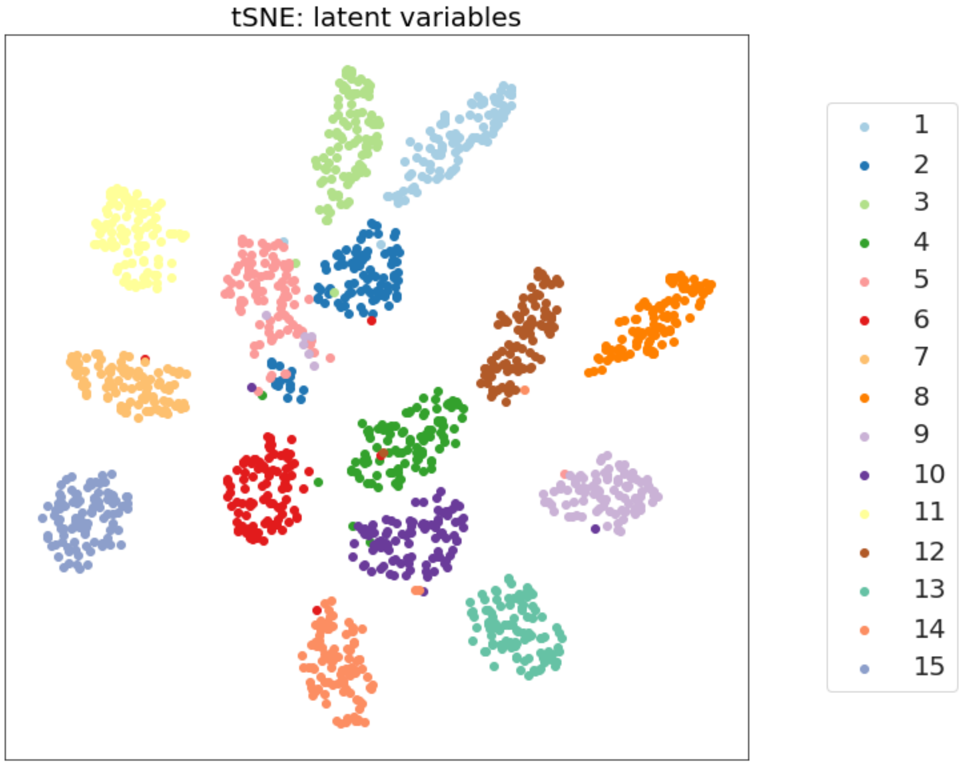

Fig. S13. t-distributed Stochastic Neighborhood Embedding (t-SNE) of data distribution.
t-SNE was trained to embed CNN outputs (before fed to softmax) into 2D space using random initialization, perplexity 50, and learning rate 200 (implemented in scikit-learn). The training dataset consists of 15 initial configurations and 12000 data points, the simulation parameters are the default. The figure illustrates the distribution of 100 randomly selected patterns from each initial configuration (each labeled by a color). The results show that the patterns encoding different characters are indeed distinguishable as t-SNE learnt to cluster them.

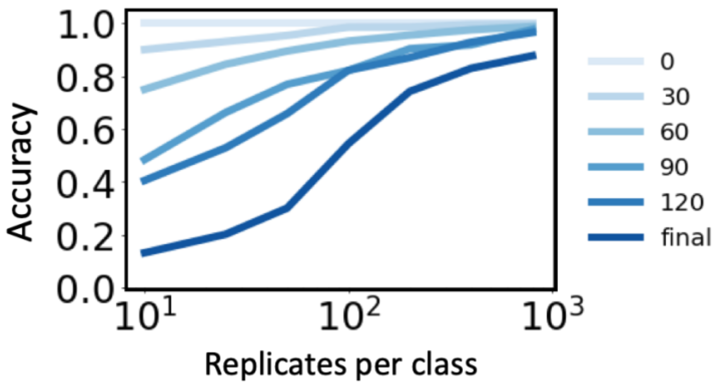

Fig. S14. Temporal information encoding using branching patterns.
Decoding accuracy on patterns arrested from growth at t = 0, 30, 60, 90, 120 and when the colony stops expanding (“final”). The patterns stopped growing at earlier time points, require less training data to achieve the same decoding accuracy.

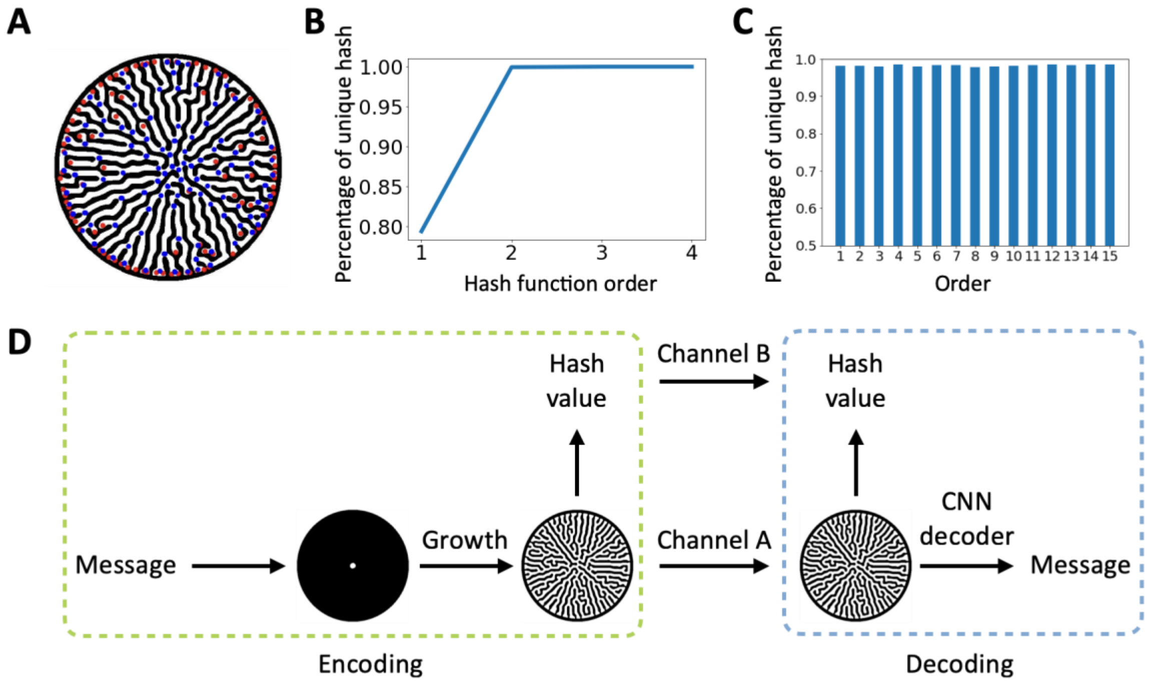

Fig. S15. Protecting information integrity using biological noise

A. Extracted minutiae (blue: bifurcation, red: branch ridge) of an example pattern.

B. The percentage of unique hashes. The hashes were computed using symmetric hash functions with order k = 1, 2, 3, and 4, respectively on a dataset of 15000 patterns (15 classes, 1000 replicates of each class). The default parameter set was used for data generation. To compute the percentage, we removed 217 duplicate patterns and normalized the counts with respect to the total number of unique patterns (14783). The figure indicates that increasing the order of hash function can reduce the chance of two different patterns sharing the same hash.

C. The percentage of unique hashes by class. The hash function here has k = 1. The calculation was carried out on the same dataset as in panel B. The percentage of unique hash was normalized with respect to the number of unique patterns for a given class. The results show that the chance of collision is comparable among all classes.

D. Integrity check procedure. In real communication, the sender would send a pattern and its hash through different channels (channel A and B, respectively) to the recipient. Once the recipient receives both, he/she would first authenticate information integrity by computing the hash of the received pattern and comparing it with the hash received through channel B. If they agree, the recipient can proceed to decode. Otherwise, reject the pattern and request a new one. This procedure is analogous to a typical two factor authentication.

Table S1. Converting encoding characters into binary representations.

For an example dictionary of 15 characters (A-E, 0-9), one possible way of encoding is to first convert them into 4-bit binary numbers in order. Binary number "0000" is purposefully avoided, because 0 corresponds to the absence of cells in the seeding configuration, thus colony patterns cannot form.

**Encoding character**	**Binary representation**	**Encode character**	**Binary representation**
A	0001	3	1001
B	0010	4	1010
C	0011	5	1011
D	0100	6	1100
E	0101	7	1101
0	0110	8	1110
1	0111	9	1111
2	1000		

Table S2. Estimated uncertainty as a function of training data size and the number of base models. We trained LR ensemble model using differently sized dataset and number of base models. For the same dataset, the estimated uncertainty does not change drastically with respect to the number of base models.

Replicates per class	Number of base models	LL	MSE	Top 1 error	Top 5 error
10	5	3.687	0.074	0.107	0.541
	4	3.675	0.074	0.108	0.537
	3	3.645	0.073	0.098	0.552
	2	3.545	0.072	0.108	0.533
100	5	1.839	0.049	0.476	0.080
	4	1.854	0.050	0.470	0.088
	3	1.903	0.051	0.448	0.097
	2	2.010	0.054	0.419	0.110
800	5	0.478	0.014	0.869	0.004
	4	0.423	0.012	0.881	0.003
	3	0.425	0.012	0.882	0.003
	2	0.446	0.012	0.884	0.002

Supplemental Experimental Procedures

Temporal information encoding and decoding

In dynamical systems, information embedded in the initial conditions may dissipate over time(1). Thus, it is necessary to determine the temporal encoding-decoding performance of our patterning system. We simulated branching patterns using the same protocol as described in the Methods. Instead of waiting until the colony stops growing, we arrested the simulation at different computational time points (Figure S1). We observed a similar trade-off among capacity, security and decoding reliability as mentioned in the main text (Figure S12). The patterns arrested at earlier time points have higher encoding capacity and lower security, as they require fewer training data to achieve the same accuracy compared to the later patterns. This is possibly because as the patterning process goes on, the colony starts developing branches that obscure the initial configuration information and shrinks the inter-categorical similarity. The accuracy curves are bounded from below by the curve of the final patterns, implying that the final patterns provide the highest security among all. Moreover, the results indicate that temporal regulation of pattern formation would be an additional strategy to modulate encoding capacity and security.

Authenticating patterns using noise signatures

Information integrity is critical for reliable communication and could be compromised under different scenarios. For instance, the attackers could alter the patterns to prevent delivering important messages or replace them with fake ones to deceive the intended recipient (ex. phishing). Therefore, it is critical to carry out an integrity check before decoding. One plausible method is through hashing, where a unique hash is generated and assigned to a message. When the pattern is tampered, the hash should change drastically (so-called *avalanche effect*) and fail to match the original one, indicating potential attacks and damage. Moreover, the hash function should be designed so that the chance of two different patterns having the same hash value (i.e., collision) is low.

In our system, branching dynamic amplifies the inherent biological noise in cell seeding and growth, resulting in patterns that are similar globally but vary in detail. We leveraged this feature and implemented hashing in the pattern-based communication platform. For a given pattern, we first locate its bifurcation and branch ridges (Figure S13A). To compute a unique hash, we input the extracted minutiae locations into a symmetric hash function(2). This class of hash functions has the advantage of being order-invariant, such that it allows us to bypass the challenge of assigning orders to minutiae. Consider an extracted pattern minutia has location (x_i, y_i), $i \in \{1,2,\dots,N\}$, where N is the total number of minutiae. We can design hash function $h_k = \sum_{i=1}^{N}(x_i^k + y_i^k)$, where k indicates the order of the function. As a proof of principle, we tested hash functions of different orders on an example dataset and found that it was possible to find order k that minimizes the chance of collision for patterns encoding different characters (Figure S13B) and patterns encoding the same characters (Figure S13C). Moreover, it is also possible and probably more robust to match the patterns by comparing multiple hash values all at once.

In practice, the end-users can use the hash values to carry out pattern integrity checks before preceding to decode, similar to two-factor authentication (Figure S13D). Here, we focused only on the malicious tampering of patterns other than transformation or numerical errors caused by minor transmission distortion. Considering the similarity between matching branching patterns and human fingerprints, further studies could design and apply more robust hash functions, such as those used on biometric data(3-5).

Encoding and decoding using elementary cellular automaton

The elementary cellular automaton (ECA) model is one-dimensional, each cell has a state of either 0 or 1. The system starts from an initial sequence of cells, defined in a similar manner as

in the Deng model (Figure S4C). Each character is first converted into a binary number, which is then translated into an initial configuration according to a predefined array. The cell status at the next time step depends on the current status of its neighbors. We implemented rule 60 that is weakly chaotic. Here 60 represents binary number 00111100, each digit represents the resulting cell status in the corresponding scenario (Figure S4A). For example, in the first scenario (Figure S4A, the most left), the cell of interest (middle) and its two neighbors have status of 111 at the current time step, the first 0 in the binary number means the cell of interest will have status 0 at the next time step.

Unless specified, the default encoding parameters are: sequence length = 450, time step = 500, spot radius = 10, dictionary size = 15, spacing = 70. Noise is imposed onto the seeding sequence before evolution starts. We draw random numbers from a uniform distribution and assign them to each cell within the initial configuration. We define a threshold ρ, cells with value smaller than ρ will flip from status 1 to 0, otherwise they will keep status 1. In other words, ρ is the percentage of cells who flip their status, larger ρ indicates larger noise level. We use default ρ of 0.5. These parameters were chosen such that the system gives satisfying security level. To decode, we trained a feedforward neural network to classify the output sequences via classification. The mathematical modeling was implemented in MATLAB R2020b, the ML model training implemented in Python3 and PyTorch 1.9.

Figure S5 shows that increasing noise level and small spacing deteriorate decoder performance, which is consistent with the observations with colony patterns. The decoding accuracy can be partially rescued by increasing the training data size. Dictionary size does not impact the performance, indicating each system have different encoding capacity and must be tuned individually.

Supplementary References

1. Gade P, Amritkar R. Characterizing loss of memory in a dynamical system. Physical review letters. 1990;65(4):389.
2. Tulyakov S, Farooq F, Govindaraju V, editors. Symmetric hash functions for fingerprint minutiae. International Conference on Pattern Recognition and Image Analysis; 2005: Springer.
3. Das P, Karthik K, Garai BC. A robust alignment-free fingerprint hashing algorithm based on minimum distance graphs. Pattern Recognition. 2012;45(9):3373-88.
4. Lai Y-L, Jin Z, Teoh ABJ, Goi B-M, Yap W-S, Chai T-Y, et al. Cancellable iris template generation based on Indexing-First-One hashing. Pattern Recognition. 2017;64:105-17.
5. Kumar G, Tulyakov S, Govindaraju V, editors. Combination of symmetric hash functions for secure fingerprint matching. 2010 20th International Conference on Pattern Recognition; 2010: IEEE.

Appendix A

Encoding characters in Emorfi

To construct Emorfi, each of the printable ASCII characters (including English letters in upper and lower cases, digits, punctuations, and whitespaces) was converted into a binary representation, which was then converted into a unique initial configuration. For each configuration, we carried out mathematical simulation and obtained 1000 different patterns. These 100 sets of patterns, as well as subsequent ones to be generated, make up Emorfi. In the table below, three examples are shown for each character.

Encoding character	Binary representation	Initial configuration	Example patterns		
0	0000001	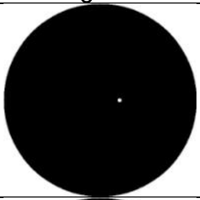	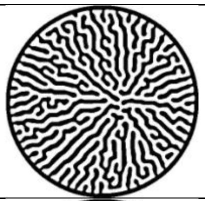	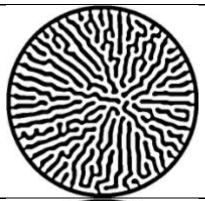	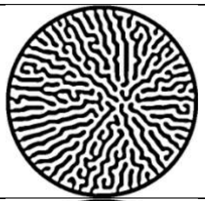
1	0000010	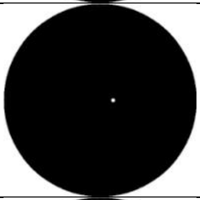	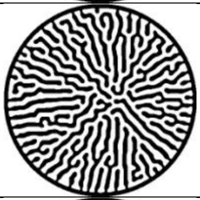	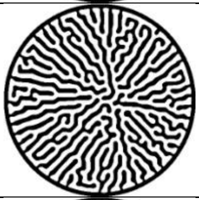	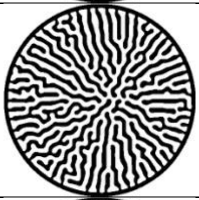
2	0000011	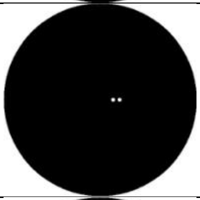	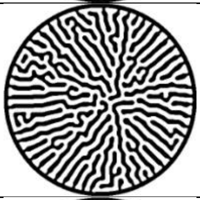	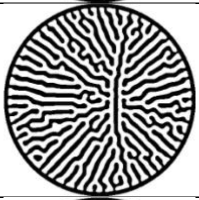	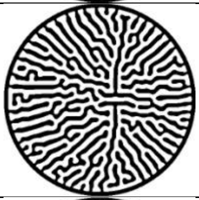
3	0000100	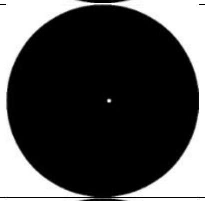	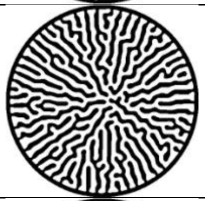	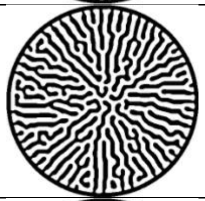	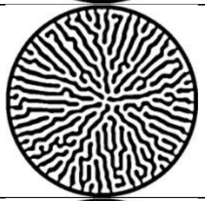
4	0000101	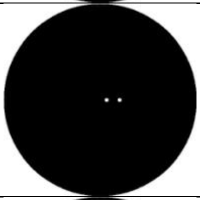	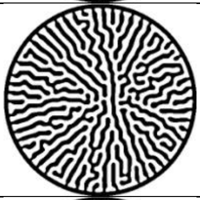	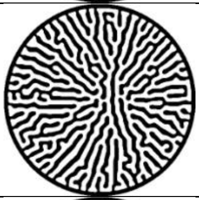	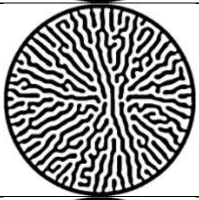
5	0000110	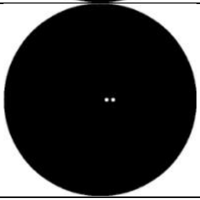	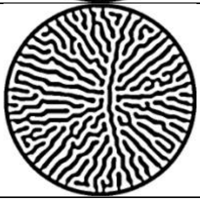	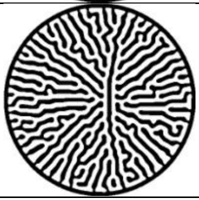	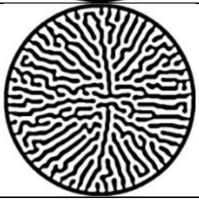

6	0000111	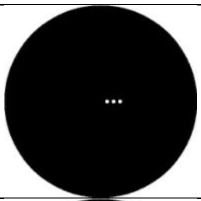	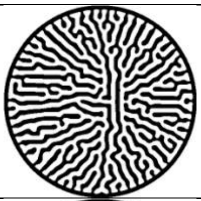	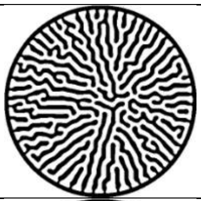	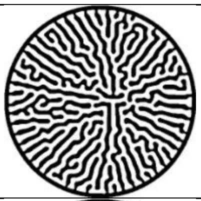
7	0001000	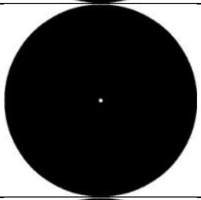	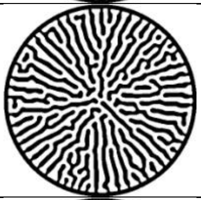	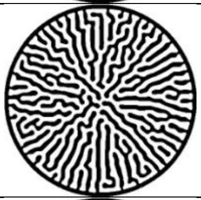	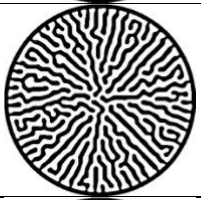
8	0001001	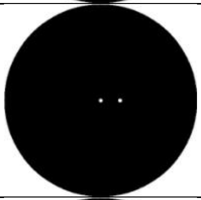	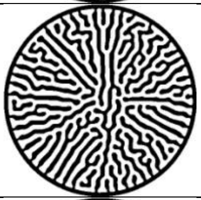	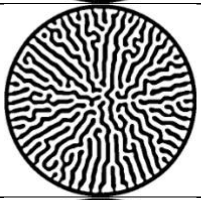	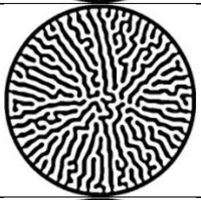
9	0001010	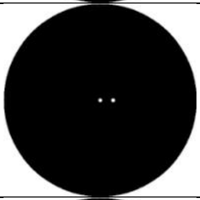	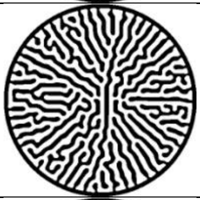	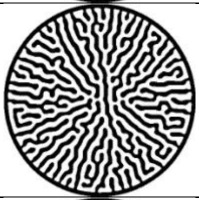	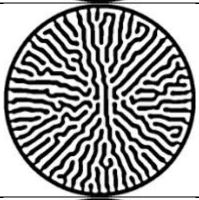
a	0001011	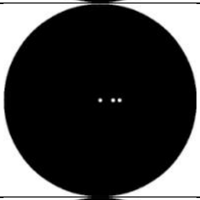	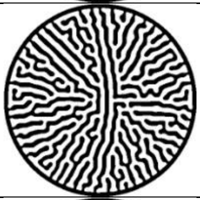	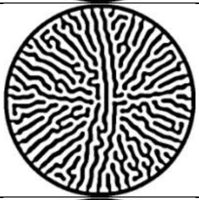	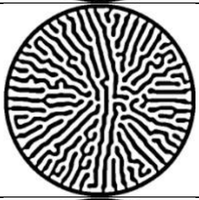
b	0001100	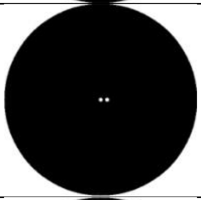	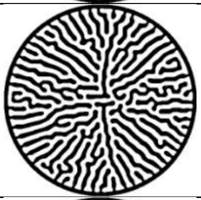	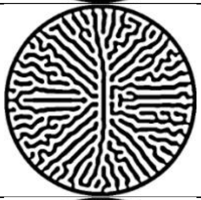	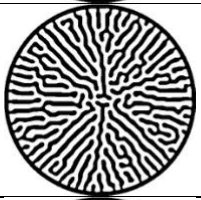
c	0001101	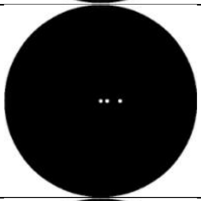	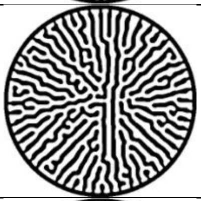	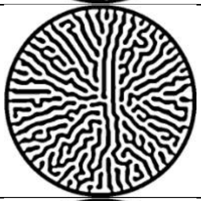	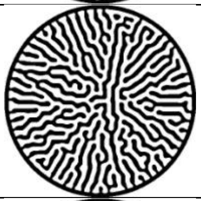
d	0001110	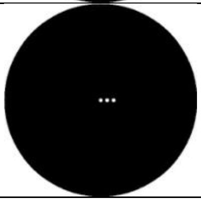	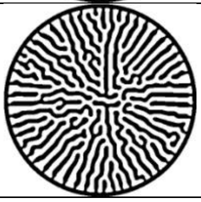	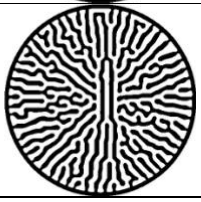	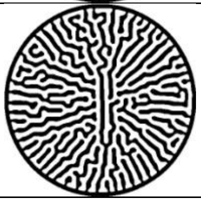

e	0001111	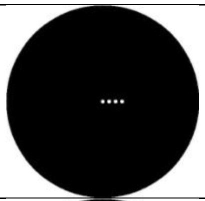	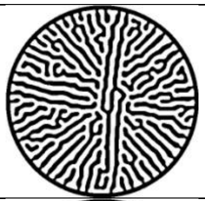	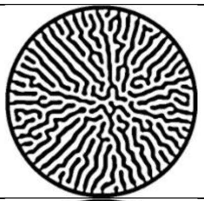	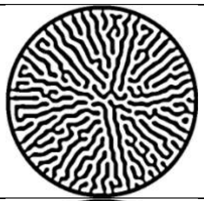
f	0010000	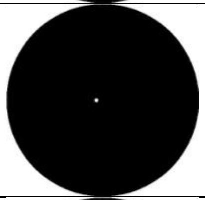	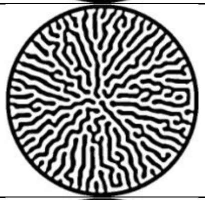	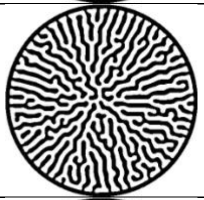	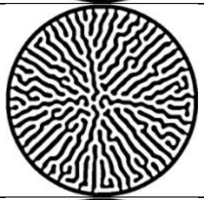
g	0010001	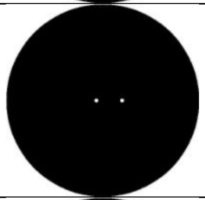	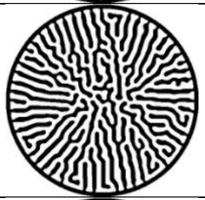	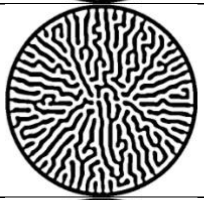	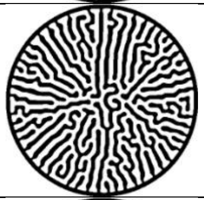
h	0010010	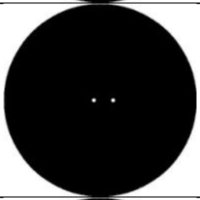	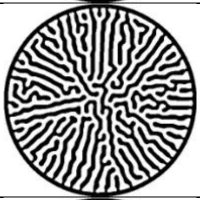	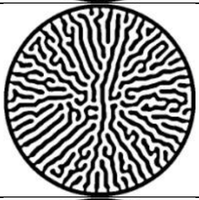	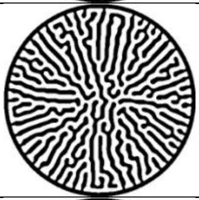
i	0010011	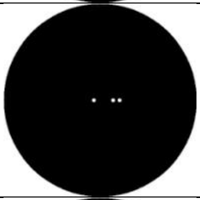	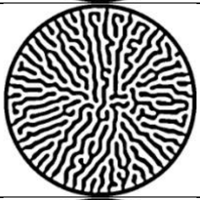	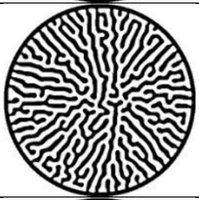	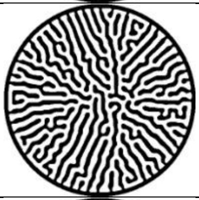
j	0010100	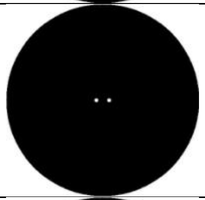	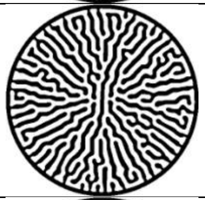	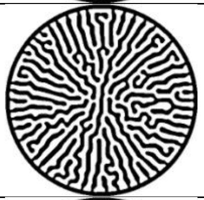	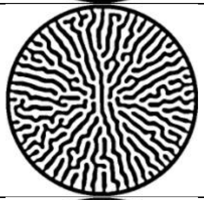
k	0010101	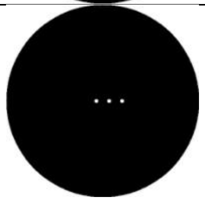	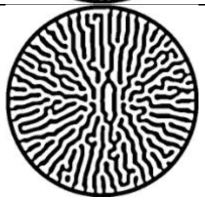	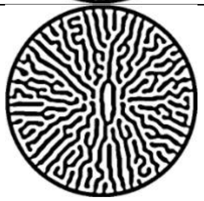	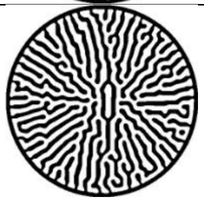
l	0010110	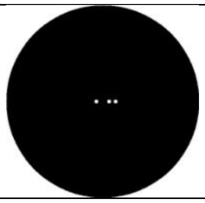	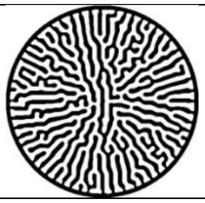	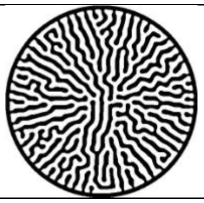	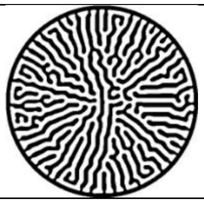

m	0010111	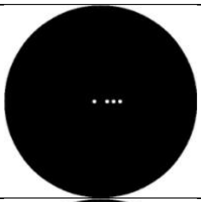	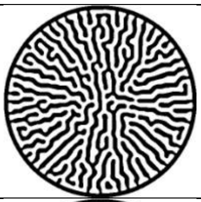	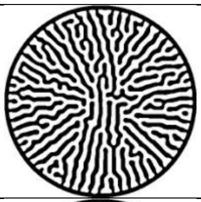	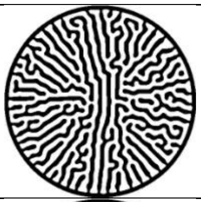
n	0011000	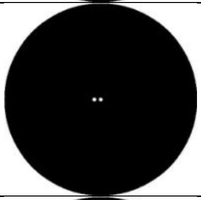	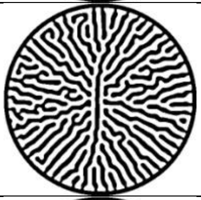	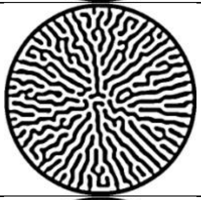	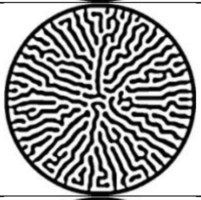
o	0011001	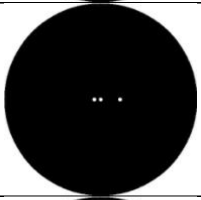	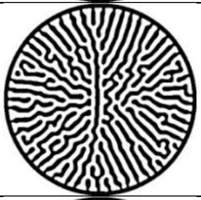	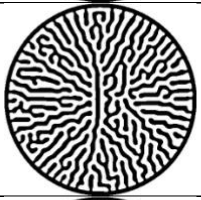	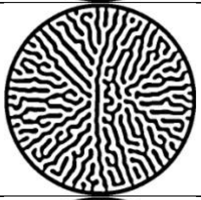
p	0011010	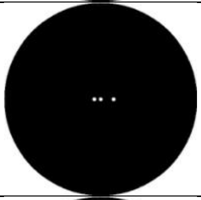	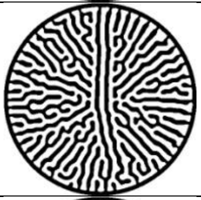	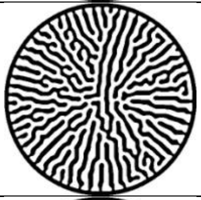	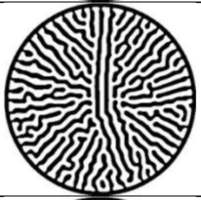
q	0011011	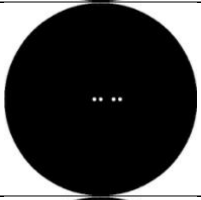	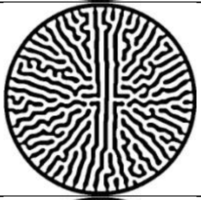	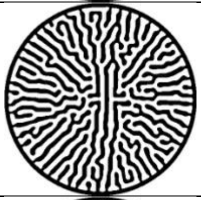	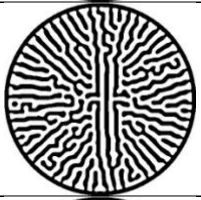
r	0011100	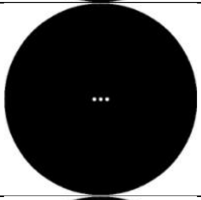	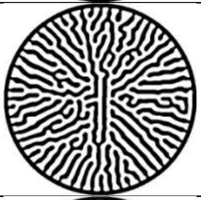	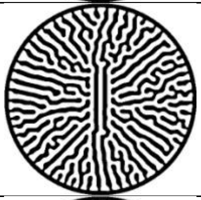	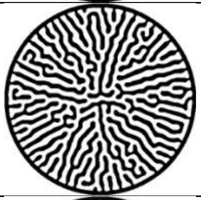
s	0011101	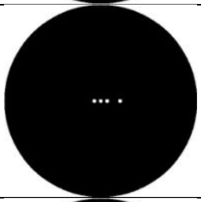	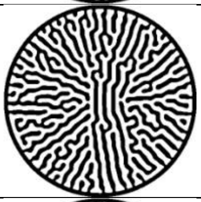	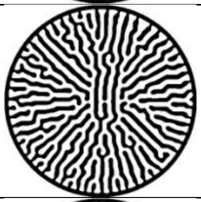	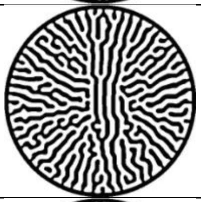
t	0011110	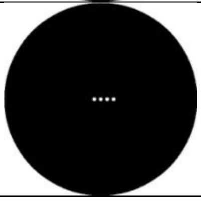	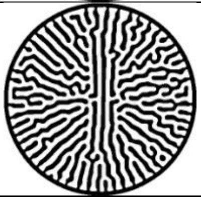	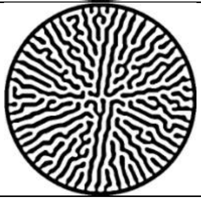	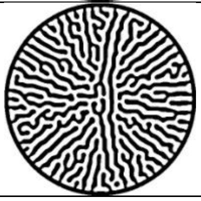

u	0011111	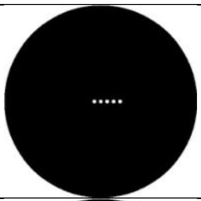	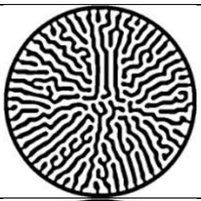	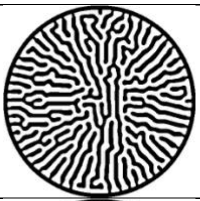	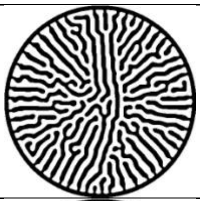
v	0100000	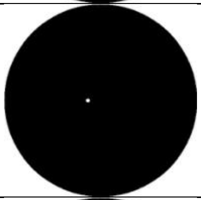	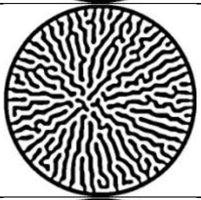	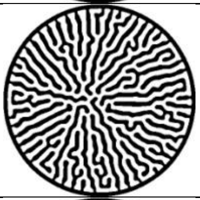	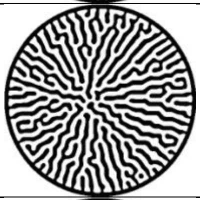
w	0100001	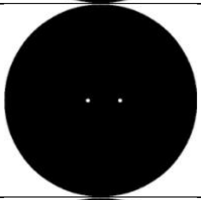	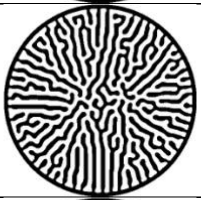	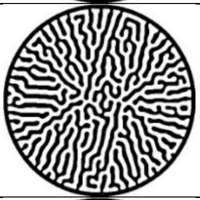	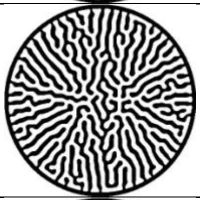
x	0100010	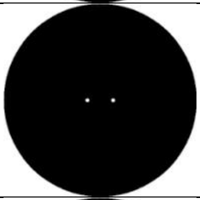	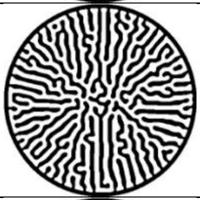	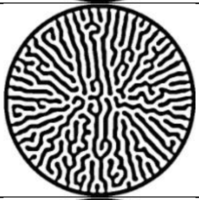	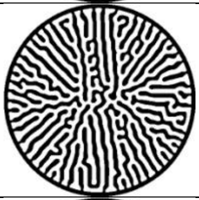
y	0100011	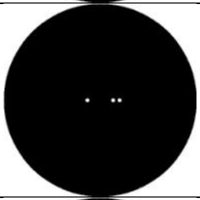	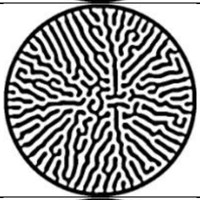	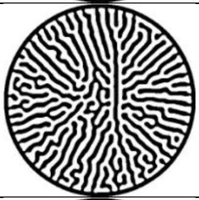	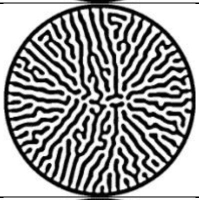
z	0100100	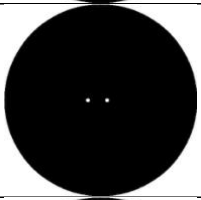	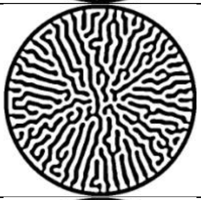	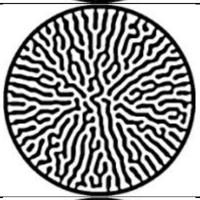	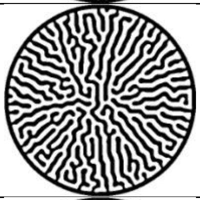
A	0100101	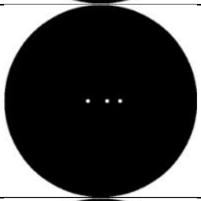	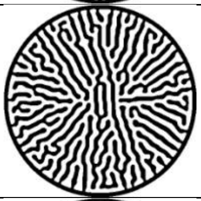	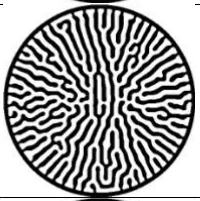	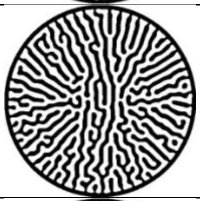
B	0100110	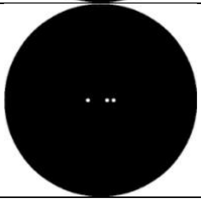	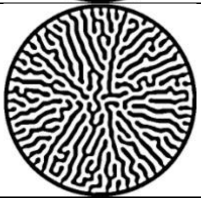	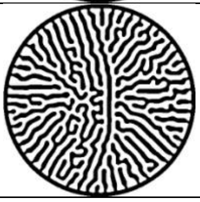	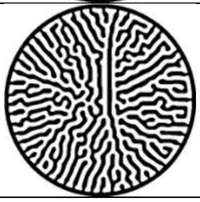

C	0100111	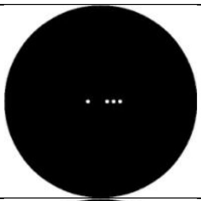	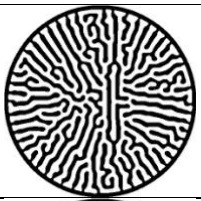	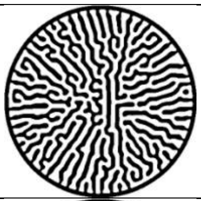	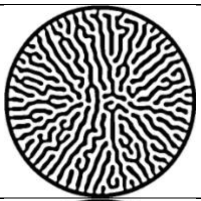
D	0101000	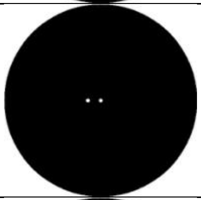	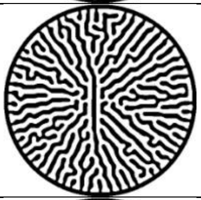	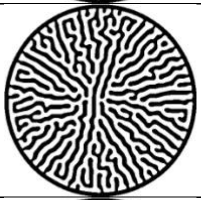	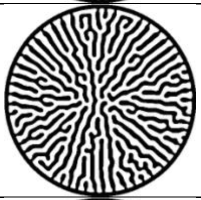
E	0101001	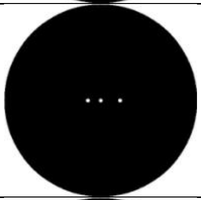	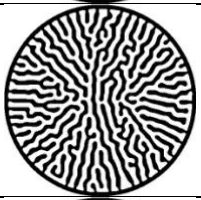	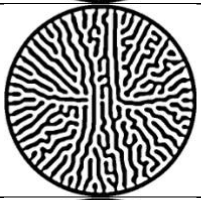	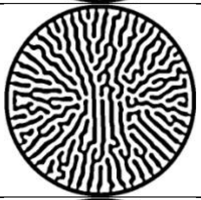
F	0101010	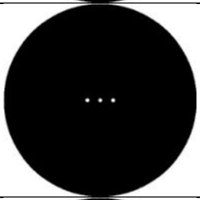	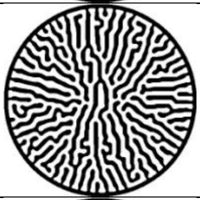	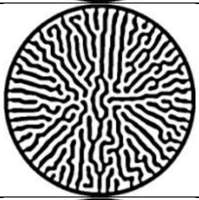	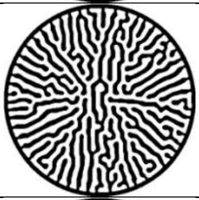
G	0101011	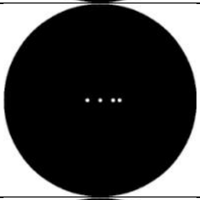	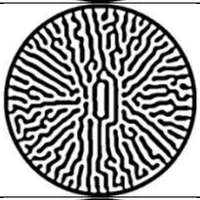	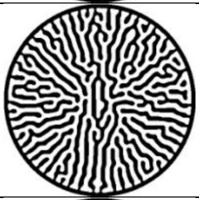	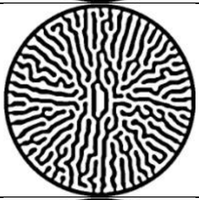
H	0101100	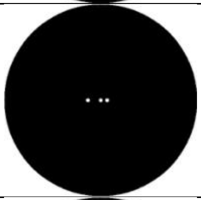	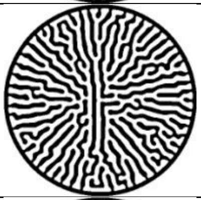	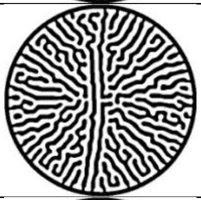	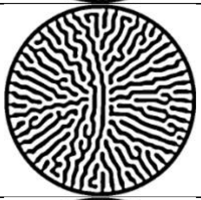
I	0101101	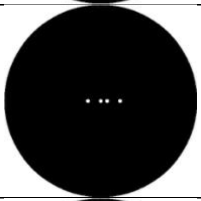	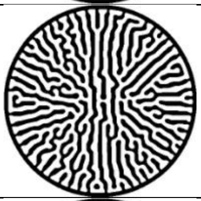	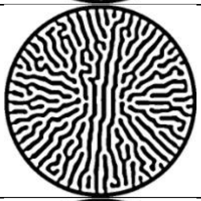	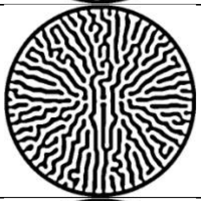
J	0101110	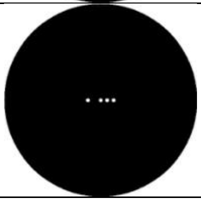	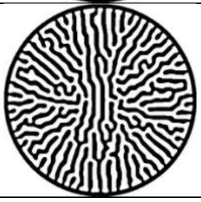	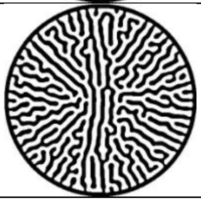	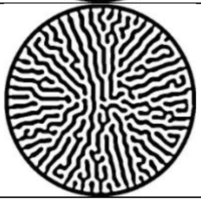

K	0101111	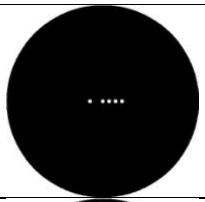	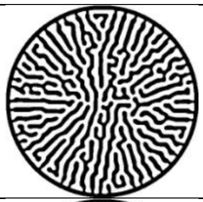	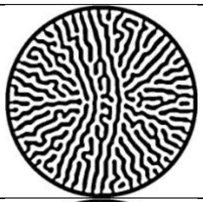	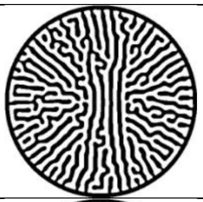
L	0110000	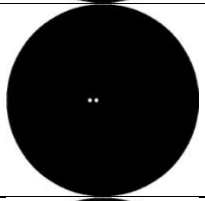	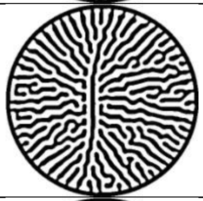	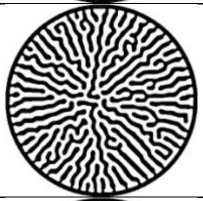	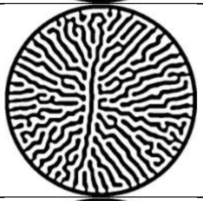
M	0110001	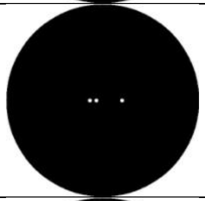	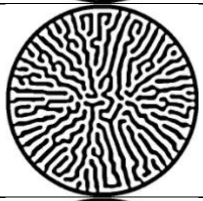	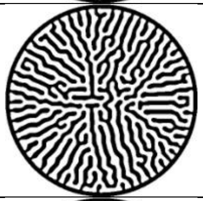	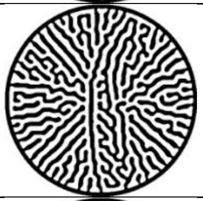
N	0110010	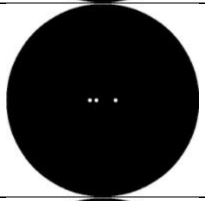	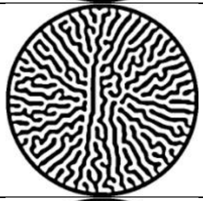	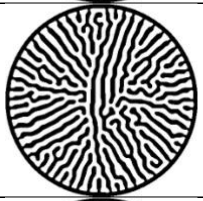	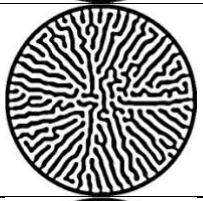
O	0110011	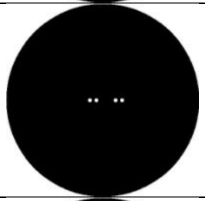	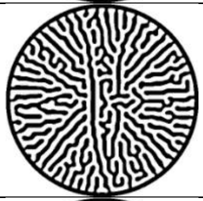	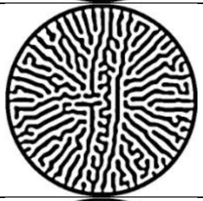	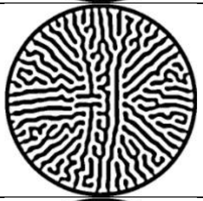
P	0110100	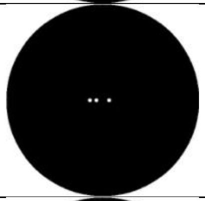	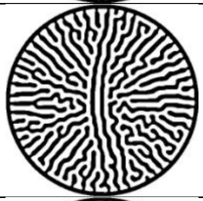	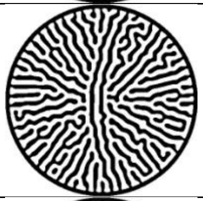	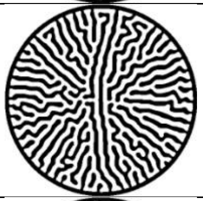
Q	0110101	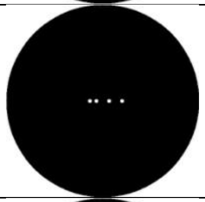	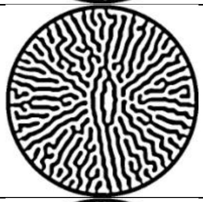	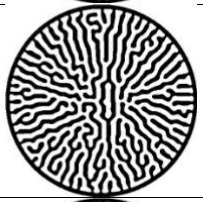	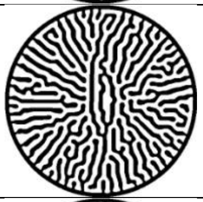
R	0110110	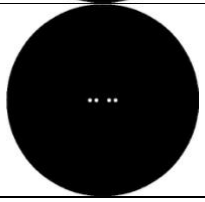	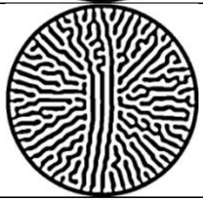	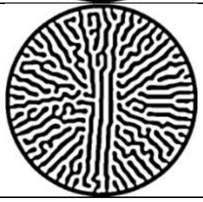	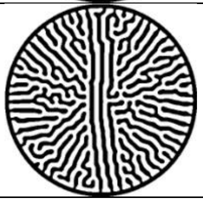

S	0110111	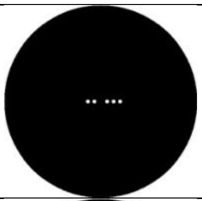	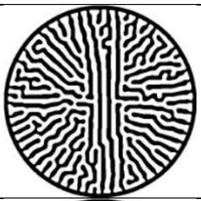	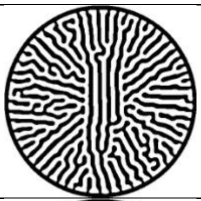	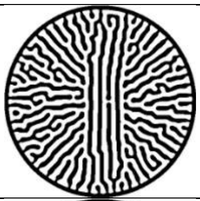
T	0111000	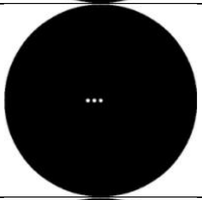	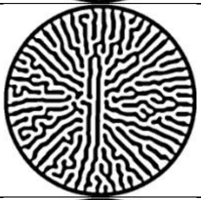	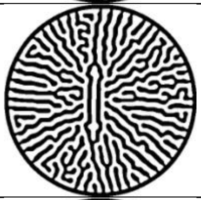	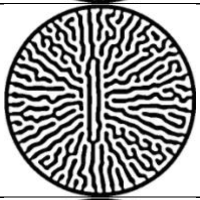
U	0111001	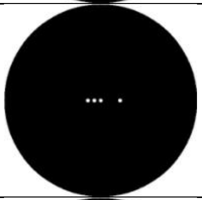	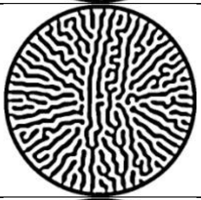	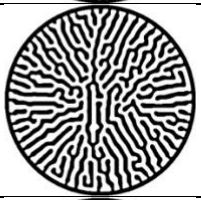	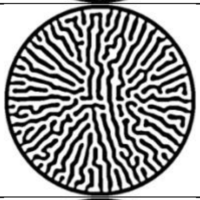
V	0111010	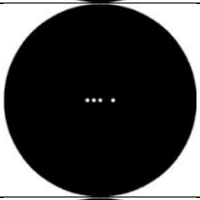	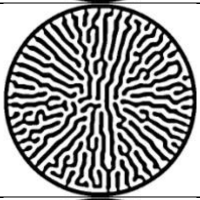	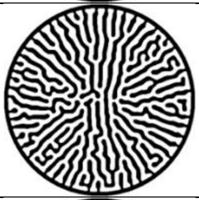	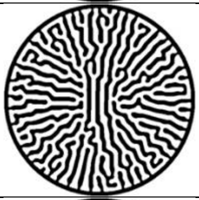
W	0111011	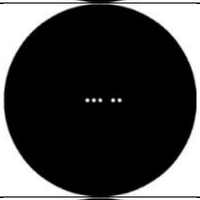	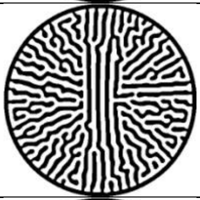	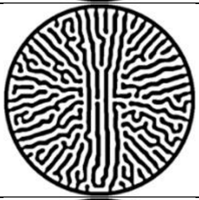	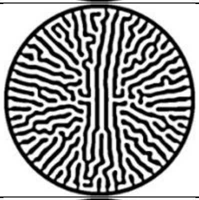
X	0111100	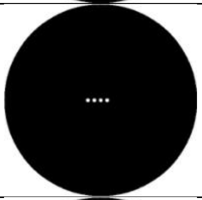	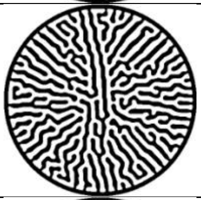	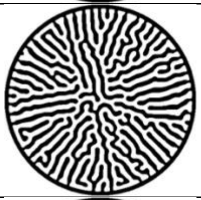	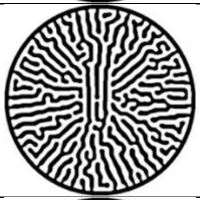
Y	0111101	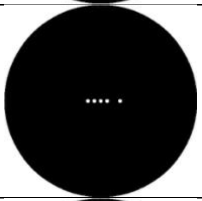	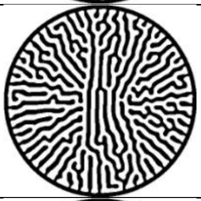	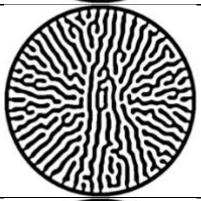	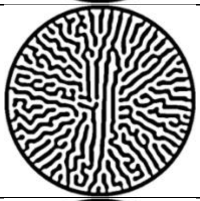
Z	0111110	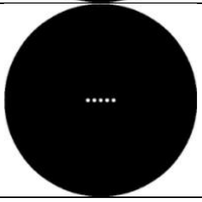	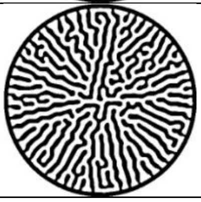	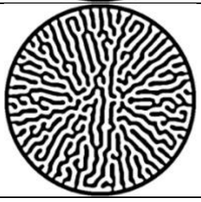	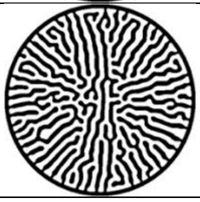

!	0111111	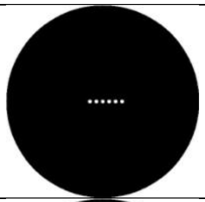	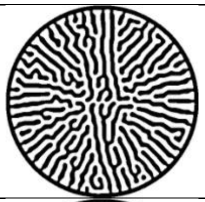	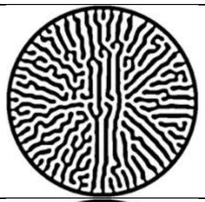	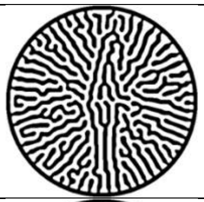
“	1000000	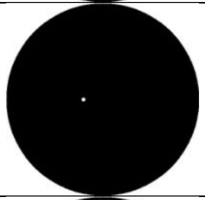	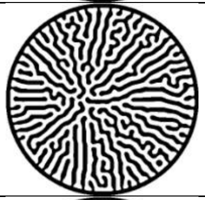	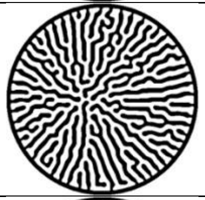	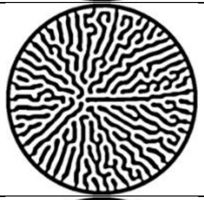
#	1000001	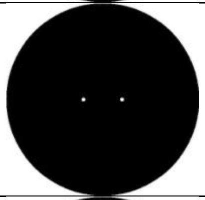	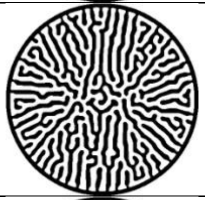	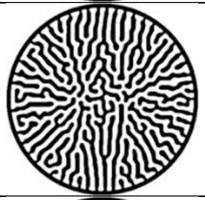	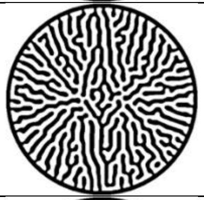
$	1000010	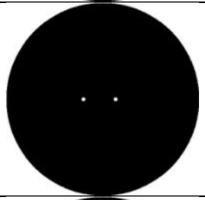	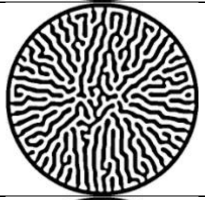	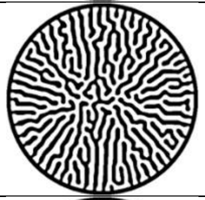	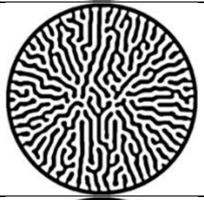
%	1000011	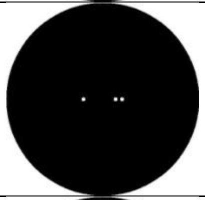	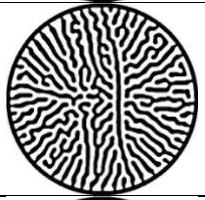	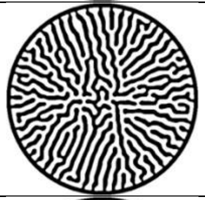	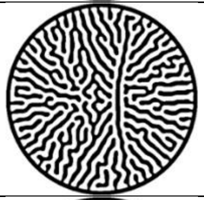
&	1000100	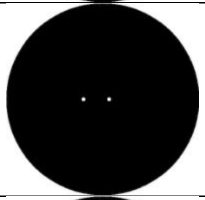	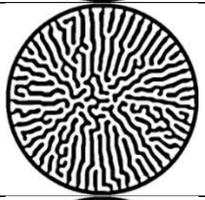	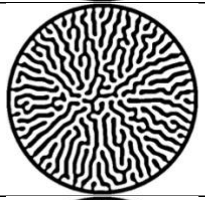	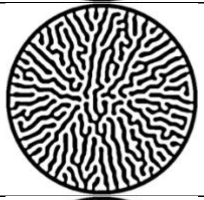
‘	1000101	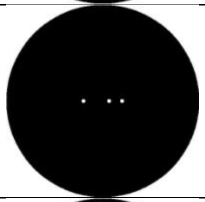	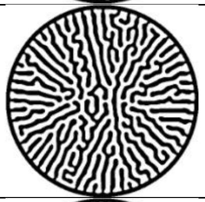	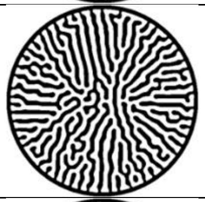	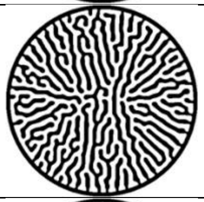
(	1000110	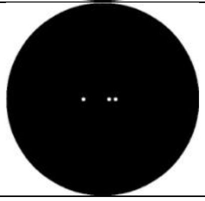	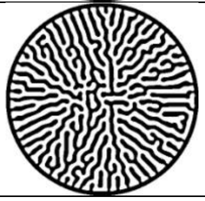	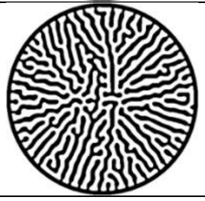	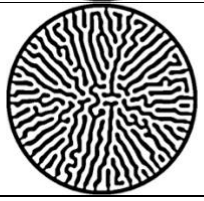

)	1000111	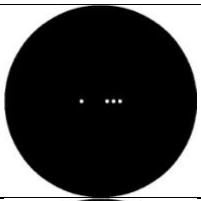	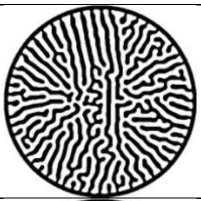	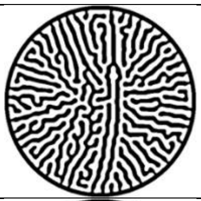	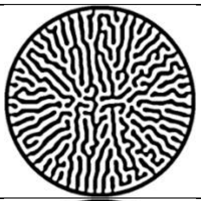
*	1001000	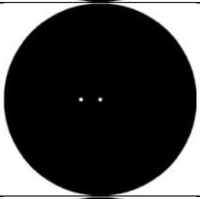	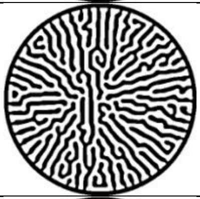	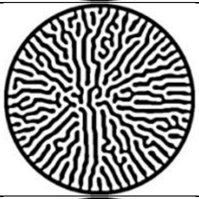	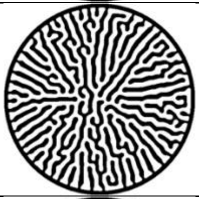
+	1001001	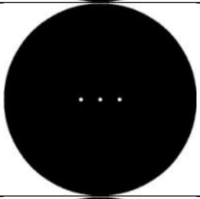	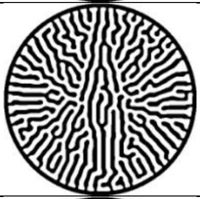	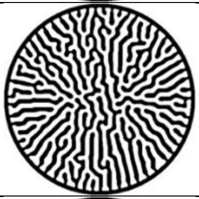	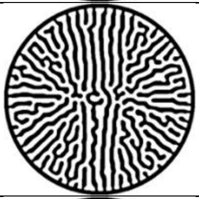
,	1001010	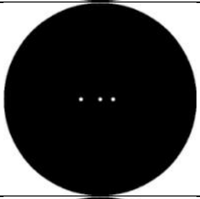	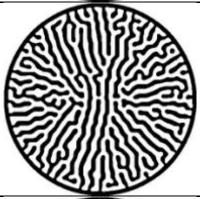	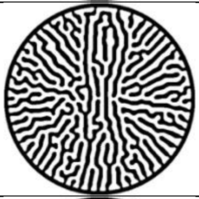	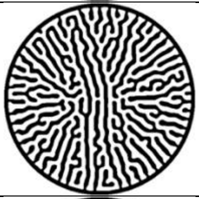
-	1001011	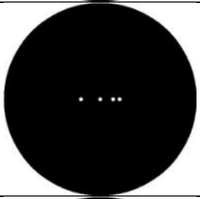	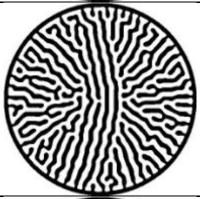	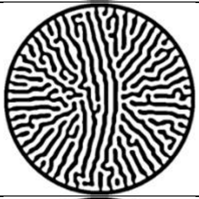	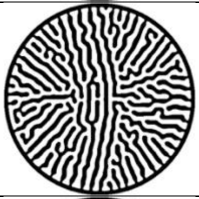
.	1001100	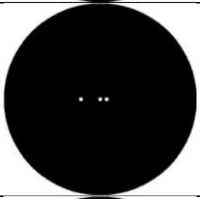	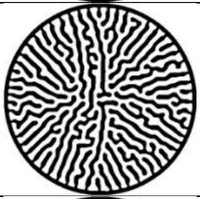	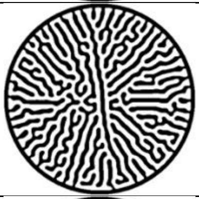	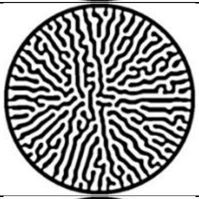
/	1001101	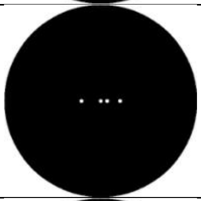	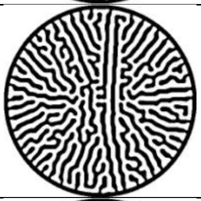	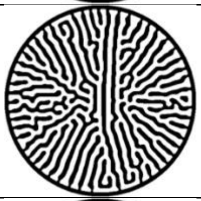	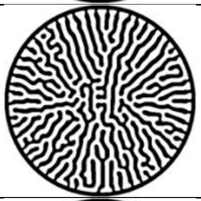
:	1001110	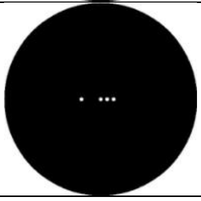	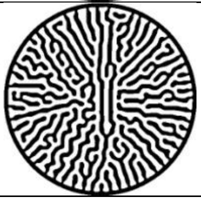	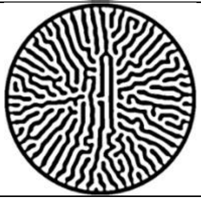	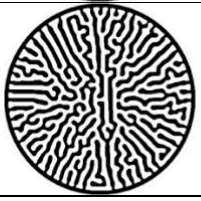

;	1001111	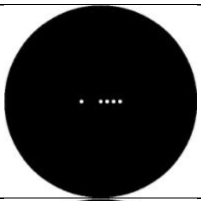	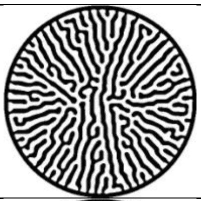	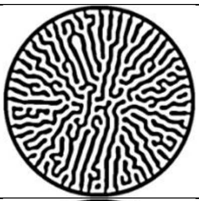	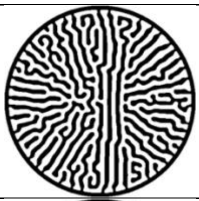
<	1010000	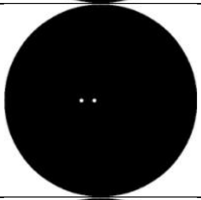	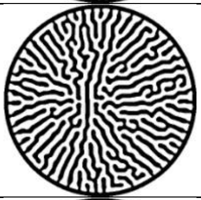	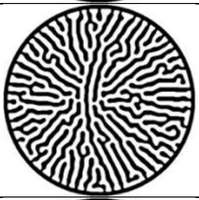	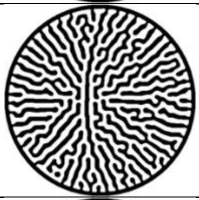
=	1010001	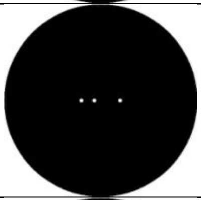	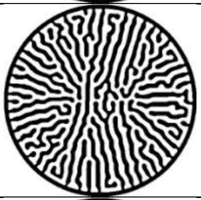	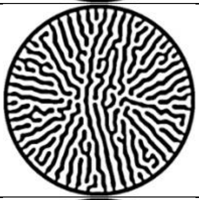	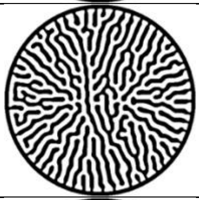
>	1010010	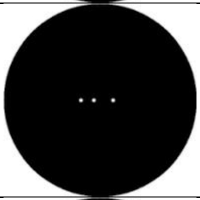	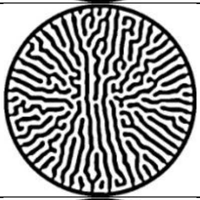	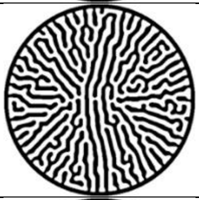	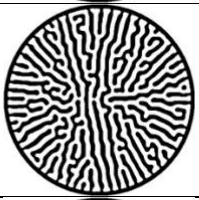
?	1010011	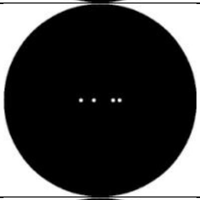	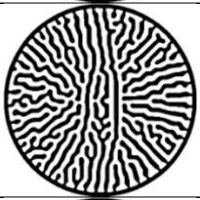	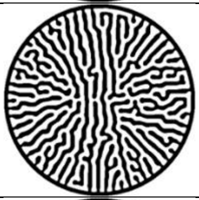	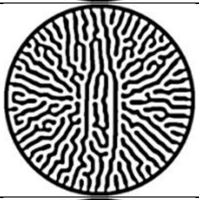
@	1010100	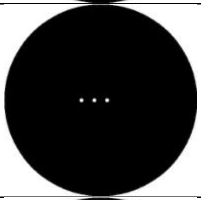	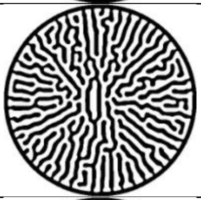	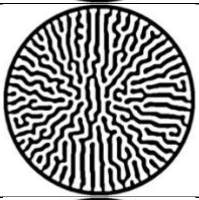	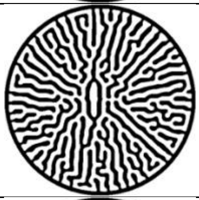
[	1010101	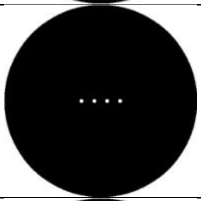	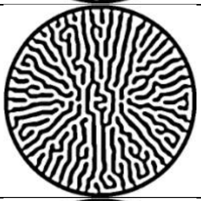	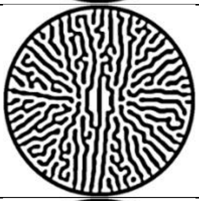	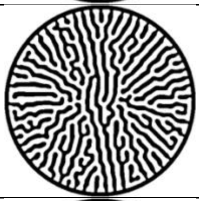
\\	1010110	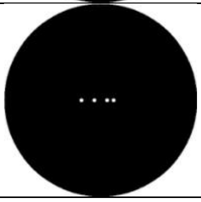	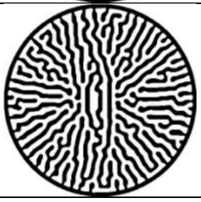	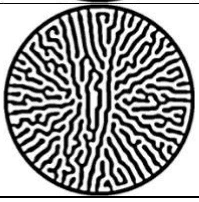	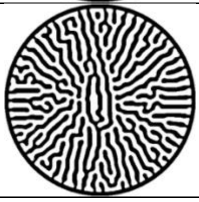

]	1010111	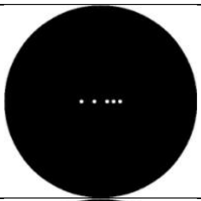	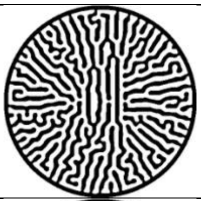	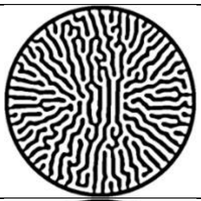	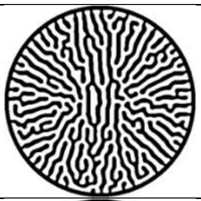
^	1011000	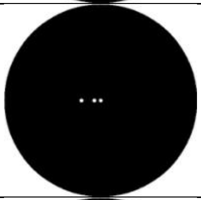	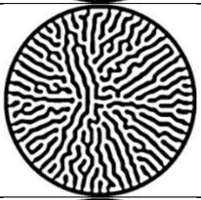	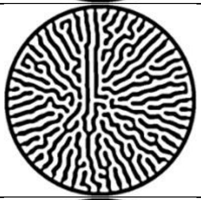	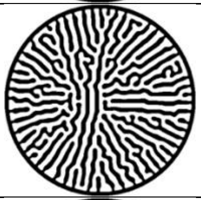
_	1011001	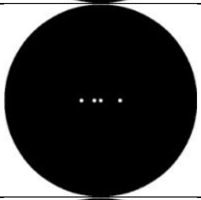	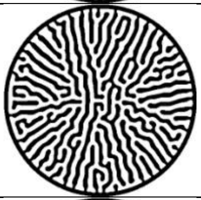	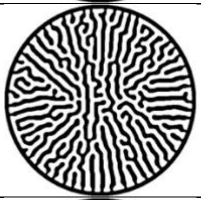	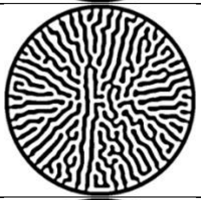
`	1011010	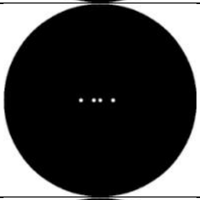	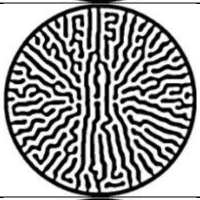	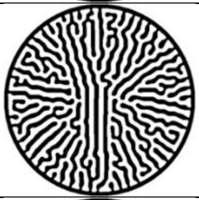	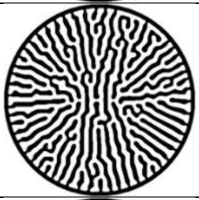
{	1011011	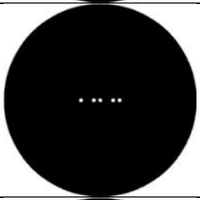	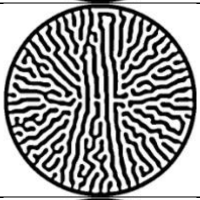	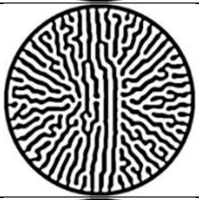	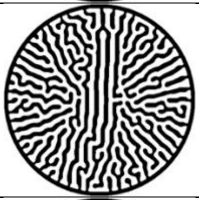
|	1011100	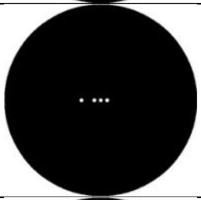	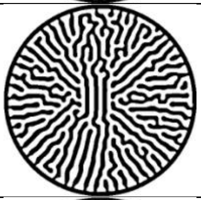	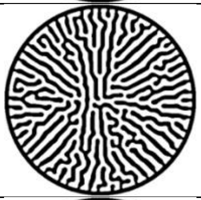	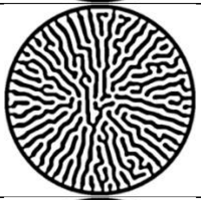
}	1011101	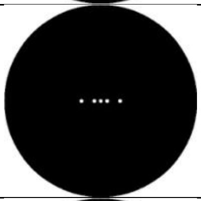	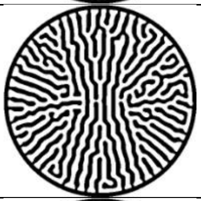	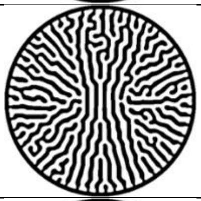	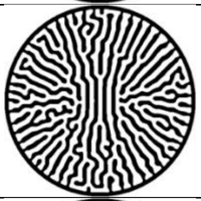
~	1011110	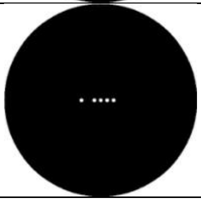	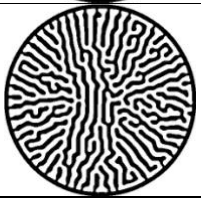	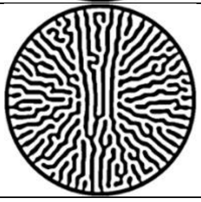	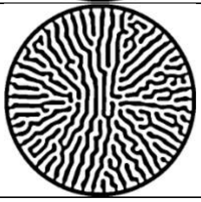

Character space	1011111	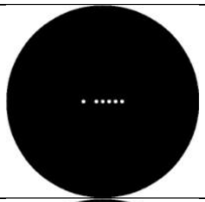	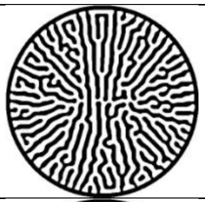	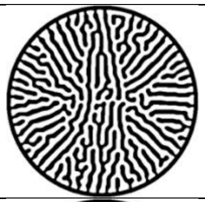	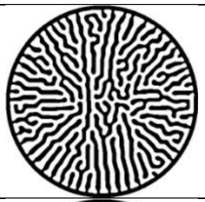
\t (tab)	1100000	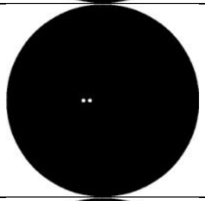	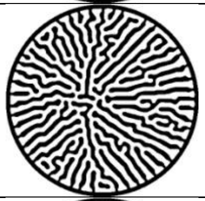	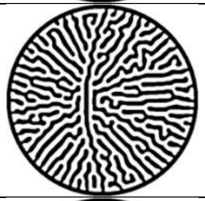	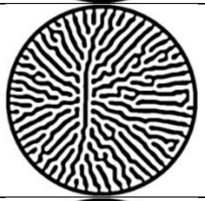
\n (linefeed)	1100001	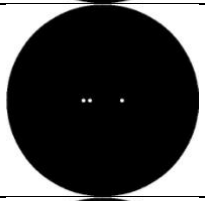	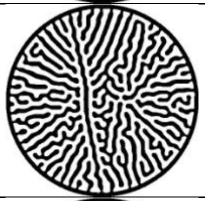	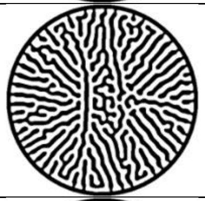	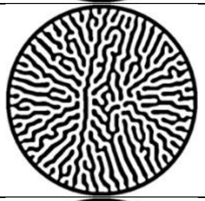
\r (return)	1100010	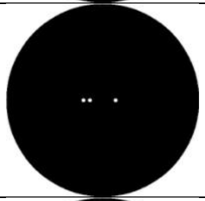	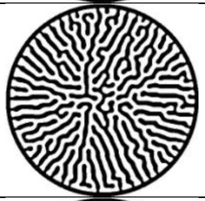	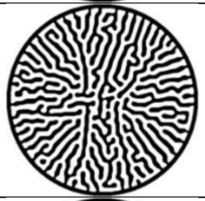	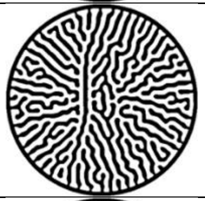
\x0b (vertical tab)	1100011	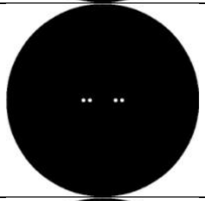	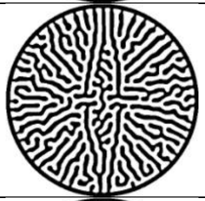	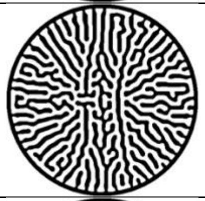	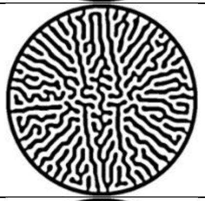
\x0c (formfeed)	1100100	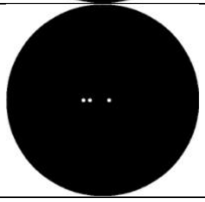	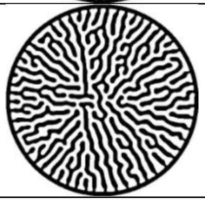	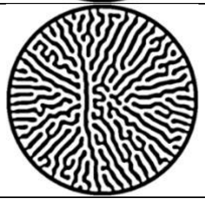	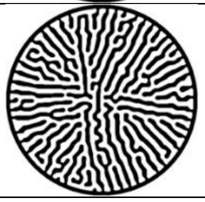

Appendix B

Encoding “I Have a Dream” in Emorfi and decoding it back in English

Each character was encoded in a randomly selected pattern from its corresponding class (see Appendix A). Next, the patterns were assembled in order into a video. To accommodate majority voting, the above process was repeated four times and the new video was concatenated to the end of the existing one. When decoding, we used a trained CNN combined with majority voting, 99.8% of the text was correctly decoded.

I Have a Dream

By Martin Luther King Jr.

August 28, 1963

Original:

Five score years ago, a great American, in whose symbolic shadow we stand today, signed the Emancipation Proclamation. This momentous decree came as a great beacon light of hope to millions of Negro slaves who had been seared in the flames of withering injustice. It came as a joyous daybreak to end the long night of their captivity. But 100 years later, the Negro still is not free. One hundred years later, the life of the Negro is still sadly crippled by the manacles of segregation and the chains of discrimination. One hundred years later, the Negro lives on a lonely island of poverty in the midst of a vast ocean of material prosperity. One hundred years later the Negro is still languished in the corners of American society and finds himself in exile in his own land. And so we've come here today to dramatize a shameful condition. In a sense we've come to our nation's capital to cash a check. When the architects of our republic wrote the magnificent words of the Constitution and the Declaration of Independence, they were signing a promissory note to which every American was to fall heir. This note was a promise that all men - yes, black men as well as white men - would be guaranteed the unalienable rights of life, liberty and the pursuit of happiness. It is obvious today that America has defaulted on this promissory note insofar as her citizens of color are concerned. Instead of honoring this sacred obligation, America has given the Negro people a bad check, a check which has come back marked insufficient funds. But we refuse to believe that the bank of justice is bankrupt. We refuse to believe that there are insufficient funds in the great vaults of opportunity of this nation. And so we've come to cash this check, a check that will give us upon demand the riches of freedom and the security of justice. We have also come to his hallowed spot to remind America of the fierce urgency of now. This is no time to engage in the luxury of cooling off or to take the tranquilizing drug of gradualism. Now is the time to make real the promises of democracy. Now is the time to rise from the dark and desolate valley of segregation to the sunlit path of racial justice. Now is the time to lift our nation from the quick sands of racial injustice to the solid rock of brotherhood. Now is the time to make justice a reality for all of God's children. It would be fatal for the nation to overlook the urgency of the moment. This sweltering summer of the Negro's legitimate discontent will not pass until there is an invigorating autumn of freedom and equality. 1963 is not an end, but a beginning. Those who hope that the Negro needed to blow off steam and will now be content will have a rude awakening if the nation returns to business as usual. There will be neither rest nor tranquility in America until the Negro is granted his citizenship rights. The whirlwinds of revolt will continue to shake the foundations of our nation until the bright day of justice emerges. But there is something that I must say to my people who stand on the warm threshold which leads into the palace of justice. In the process of gaining our rightful place, we must not be guilty of wrongful deeds. Let us not seek to satisfy our thirst for freedom by drinking from the cup of bitterness and hatred. We must forever conduct our struggle on the high plane of dignity and discipline. We must not allow our creative protest to degenerate into physical violence. Again and again, we must rise to the majestic heights of meeting physical force with soul force. The marvelous new militancy which has engulfed the Negro community must not lead us to a distrust of all white people, for many of our white brothers, as evidenced by their

presence here today, have come to realize that their destiny is tied up with our destiny. And they have come to realize that their freedom is inextricably bound to our freedom. We cannot walk alone. And as we walk, we must make the pledge that we shall always march ahead. We cannot turn back. There are those who are asking the devotees of civil rights, when will you be satisfied? We can never be satisfied as long as the Negro is the victim of the unspeakable horrors of police brutality. We can never be satisfied as long as our bodies, heavy with the fatigue of travel, cannot gain lodging in the motels of the highways and the hotels of the cities. We cannot be satisfied as long as the Negro's basic mobility is from a smaller ghetto to a larger one. We can never be satisfied as long as our children are stripped of their selfhood and robbed of their dignity by signs stating: for whites only. We cannot be satisfied as long as a Negro in Mississippi cannot vote and a Negro in New York believes he has nothing for which to vote. No, no, we are not satisfied, and we will not be satisfied until justice rolls down like waters, and righteousness like a mighty stream. I am not unmindful that some of you have come here out of great trials and tribulations. Some of you have come fresh from narrow jail cells. Some of you have come from areas where your quest for freedom left you battered by the storms of persecution and staggered by the winds of police brutality. You have been the veterans of creative suffering. Continue to work with the faith that unearned suffering is redemptive. Go back to Mississippi, go back to Alabama, go back to South Carolina, go back to Georgia, go back to Louisiana, go back to the slums and ghettos of our Northern cities, knowing that somehow this situation can and will be changed. Let us not wallow in the valley of despair, I say to you today, my friends. So even though we face the difficulties of today and tomorrow, I still have a dream. It is a dream deeply rooted in the American dream. I have a dream that one day this nation will rise up and live out the true meaning of its creed: We hold these truths to be self-evident, that all men are created equal. I have a dream that one day on the red hills of Georgia, the sons of former slaves and the sons of former slave owners will be able to sit down together at the table of brotherhood. I have a dream that one day even the state of Mississippi, a state sweltering with the heat of injustice, sweltering with the heat of oppression will be transformed into an oasis of freedom and justice. I have a dream that my four little children will one day live in a nation where they will not be judged by the color of their skin but by the content of their character. I have a dream today. I have a dream that one day down in Alabama with its vicious racists, with its governor having his lips dripping with the words of interposition and nullification, one day right down in Alabama little black boys and black girls will be able to join hands with little white boys and white girls as sisters and brothers. I have a dream today. I have a dream that one day every valley shall be exalted, every hill and mountain shall be made low, the rough places will be made plain, and the crooked places will be made straight, and the glory of the Lord shall be revealed, and all flesh shall see it together. This is our hope. This is the faith that I go back to the South with. With this faith, we will be able to hew out of the mountain of despair a stone of hope. With this faith we will be able to transform the jangling discords of our nation into a beautiful symphony of brotherhood. With this faith we will be able to work together, to pray together, to struggle together, to go to jail together, to stand up for freedom together, knowing that we will be free one day. This will be the day when all of God's children will be able to sing with new meaning: My country, 'tis of thee, sweet land of liberty, of thee I sing. Land where my fathers died, land of the pilgrims' pride, from every mountainside, let freedom ring. And if America is to be a great nation, this must become true. And so let freedom ring from the prodigious hilltops of New Hampshire. Let freedom ring from the mighty mountains of New York. Let freedom ring from the heightening Alleghenies of Pennsylvania. Let freedom ring from the snowcapped Rockies of Colorado. Let freedom ring from the curvaceous slopes of California. But not only that, let freedom ring from Stone Mountain of Georgia. Let freedom ring from Lookout Mountain of Tennessee. Let freedom ring from every hill and molehill of Mississippi. From every mountainside, let freedom ring. And when this happens, and when we allow freedom ring, when we let it ring from every village and every hamlet, from every state and every city, we will be able to speed up that day when all of God's children, black men and white men, Jews and Gentiles, Protestants and Catholics, will be able to join hands and sing in the words of the old Negro spiritual: Free at last. Free at last. Thank God almighty, we are free at last.

Decoded: (the decoding accuracy is 99.8 %, the mispredicted characters are labeled in red)

Five score years ago, a great American, in whose symbolic shadow we stand today, signed the Emancipation Proclamation. This momentous decree came as a great beacon light of hope to millions of Negro slaves who had been seared in the flames of withering injustice. It came as a joyous daybreak to end the long night of their captivity. But 100 years later, the Negro still is not free. One hundred years later, the life of the Negro is still sadly crippled by the manacles of segregation and the chains of discrimination. One hundred years later, the Negro lives on a lonely island of poverty in the midst of a vast ocean of material prosperity. One hundred years later the Negro is still languished in the corners of American society and finds himself in exile in his own land. And so we've come here today to dramatize a shameful condition. In a sense we've come to our nation's capital to cash a check. When the architects of our republic wrote the magnificent words of the Constitution and the Declaration of Independence, they were signing a promissory note to which every American was to fall heir. This note was a promise that all men - yes, black men as well as white men - would be guaranteed the unalienable rights of life, liberty and the pursuit of happiness. It is obvious today that America has defaultcd on this promissory note insofar as her citizens of color are concerned. Instead of honoring this sacred obligation, America has given the Negro people a bad check, a check which has come back marked insufficient funds. But we refuse to believe that the bank of justice is bankrupt. We refuse to believe that there are insufficient funds in the great vaults of opportunity of this nation. And so we've come to cash this check, a check that will give us upon demand the riches of freedom and the security of justice. We have also come to his hallowed spot to remind America of the fierce urgency of now. This is no time to engage in the luxury of cooling off or to take the tranquilizing drug of gradualism. Now is the time to make real the promises of democracy. Now is the time to rise from the dark and desolate valley of segregation to the sunlit path of racial justice. Now is the time to lift our nation from the quick sands of racial injustice to the solid rock of brotherhood. Now is the time to make justice a reality for all of God's children. It would be fatal for the nation to overlook the urgency of the moment. This sweltering summer of the Negro's legitimate discontent will not pass until there is an invigorating autumn of freedom and equality. 1963 is not an end, but a beginning. Those who hope that the Negro needed to blow off steam and will now be content will have a rude awakening if the nation returns to business as usual. There will be neither rest nor tranquility in America until the Negro is granted his citizenship rights. The whirlwinds of revolt will continue to shake the foundations of our nation until the bright day of justice emerges. But there is something that I must say to my people who stand on the warm threshold which leads into the palace of justice. In the process of gaining our rightful place, we must not be guilty of wrongful de**8**ds. Let us not seek to satisfy our thirst for freedom by drinking from the cup of bitterness and hatred. We must forever conduct our struggle on the high plane of dignity and discipline. We must not allow our creative protest to degenerate into physical violence. Again and again, we must rise to the majestic heights of meeting physical force with soul force. The marvelous new militancy which has engulfed the Negro community must not lead us to a distrust of all white people, for many of our white brothers, as evidenced by their presence here today, have come to realize that their destiny is tied up with our destiny. And they have come to realize **u**hat their freedom is inextricably bound to our freedom. We cannot walk alone. And as we walk, we must make the pledge that we shall always march ahead. We cannot turn back. There are those who are asking the devotees of civil rights, when will you be satisfied? We can never be satisfied as long as the Negro is the victim of the unspeakable horrors of police brutality. We can never be satisfied as**{**long as our bodies, heavy with the fatigue of travel, cannot gain lodging in the motels of the highways and the hotels of th**d** cities. We cannot be satisfied as long as the Negro's basic mobility is from a smaller ghetto to a larger one. We can nev**q**r be satisfied as long as our children are stripped of their selfhood and robbed of their dignity by signs stating: for whites only. We cannot be satisfied as long as a Negro in Mississippi cannot vote and a Negro in New York believes he has nothing for which to vote. No, no, we are not satisfied, and we will not be satisfied until justice rolls down like waters, and righteousness like a mighty stream. I am not unmindful that some of you have come here out of great trials and tribulations. Some of you have come fresh from narrow jail cells. Some of you have come from areas where your quest for freedom left you battered by the storms of persecution and staggered by the winds of police brutality. You have been the veterans of creative suffering. Continue to work with the faith that unearned suffering is redemptive. Go back to Mississippi, go back to Alabama, go back to South Carolina, go back to Georgia, go back to Louisiana, go back to the slums and ghettos of our Northern cities, knowing

that somehow this situation can and will be changed. Let us not wallow in{the valley of despair, I say to you today, my friends. So even though we face the difficulties of today and tomorrow, I still have a dream. It is a dream deeply rooted in the American dream. I have a dream that one day this nation will rise up and live out the true meaning of its creed:{We hold these truths to be self-evident, that all men are created equal. I have a dream that one day on the red hills of Georgia, the sons of former slaves and the sons of former slave owners will be able to sit{down together at the table of brotherhood. I have a dream that one day even the state of Mississippi, a state sweltering with the heat of injustice, sweltering with the heat of oppression will be transformed into an oasis of freedom and justice. I have a dream that my four little children will one day live in a nation where they will not be judged by the color of their skin but by the content of their character. I have a dream today. I have a dream that one day down in Alabama with its vicious racists, with its governor having his lips dripping with the words of interposition and~nullification, one day right down in Alabama little black boys and black girls will be able to join hands with little white boys and white girls as sisters and brothers. I have a dream today. I have a dr**u**am that one day every valley shall be exalted, every hill and mountain shall b**8** made{low, the rough places will be made plain, and the crooked places will be made straight, and the glory of the Lord shall be revealed, and all flesh shall see it together. This is our hope. This is the faith that I go_back to the South with. With this faith, we will be able to hew out of the mountain of despair a stone of hope. With this faith we will be able to transform the jangling discords of our nation into a beautiful symphony of brotherhood. With this faith we will{be able to work together, to pray together, to struggle together, to go to jail together, to stand up for freedom together, knowing that we will be free one day. This will be the day when all of God's children will be able to sing with new meaning: My country, 'tis of thee, sweet land of liberty, of thee I sing. Land where my fathers died, land of the pilgrims' pride, from every mountainside, let freedom ring. And if America is to be a great nation, this must become true. And so let freedom ring from the prodigious hilltops of New Hampshire. Let freedom ring from the mighty mountains of New York. Let freedom ring from the heightening Alleghenies of Pennsylvania. Let freedom ring from the snowcapped Rockies of Colorado. Let freedom ring from the curvaceous slopes of California. But not only that, let freedom ring from Stone Mountain of Georgia. Let freedom ring from Lookout Mountain of Tennessee. Let freedom ring from every hill{and molehill of Mississippi. From every mountainside, let freedom ring. And when this happens, and when we allow freedom ring, when we let it ring from every village and every hamlet, from every state and every city, we will be able to speed up that day when all of God's children, black men and white men, Jews and Gentiles, Protestants and Catholics, will be able to join hands and sing in the words of the old N**c**gro spiritual: Fr**8**e at last. Free at last. Thank God almighty, we are free at last.

Appendix C

Encoding “Auguries of Innocence” in Emorfi and decoding it back in English

Each character was encoded in a randomly selected pattern from its corresponding class (see Appendix A). Next, the patterns were assembled in order into a video. To accommodate majority voting, the above process was repeated four times and the new video was concatenated to the end of the existing one. When decoding, we used a trained CNN combined with majority voting, 99.6% of the text was correctly decoded.

Auguries of Innocence

By William Blake

1863

Original (Poetry foundation):

To see a World in a Grain of Sand
And a Heaven in a Wild Flower
Hold Infinity in the palm of your hand
And Eternity in an hour
A Robin Red breast in a Cage
Puts all Heaven in a Rage
A Dove house filld with Doves & Pigeons
Shudders Hell thr' all its regions
A dog starvd at his Masters Gate
Predicts the ruin of the State
A Horse misusd upon the Road
Calls to Heaven for Human blood
Each outcry of the hunted Hare
A fibre from the Brain does tear
A Skylark wounded in the wing
A Cherubim does cease to sing
The Game Cock clipd & armd for fight
Does the Rising Sun affright
Every Wolfs & Lions howl
Raises from Hell a Human Soul
The wild deer, wandring here & there
Keeps the Human Soul from Care
The Lamb misusd breeds Public Strife
And yet forgives the Butchers knife
The Bat that flits at close of Eve
Has left the Brain that wont Believe
The Owl that calls upon the Night
Speaks the Unbelievers fright
He who shall hurt the little Wren
Shall never be belovd by Men
He who the Ox to wrath has movd
Shall never be by Woman lovd
The wanton Boy that kills the Fly
Shall feel the Spiders enmity
He who torments the Chafers Sprite
Weaves a Bower in endless Night
The Catterpiller on the Leaf
Repeats to thee thy Mothers grief

Kill not the Moth nor Butterfly
For the Last Judgment draweth nigh
He who shall train the Horse to War
Shall never pass the Polar Bar
The Beggars Dog & Widows Cat
Feed them & thou wilt grow fat
The Gnat that sings his Summers Song
Poison gets from Slanders tongue
The poison of the Snake & Newt
Is the sweat of Envys Foot
The poison of the Honey Bee
Is the Artists Jealousy
The Princes Robes & Beggars Rags
Are Toadstools on the Misers Bags
A Truth thats told with bad intent
Beats all the Lies you can invent
It is right it should be so
Man was made for Joy & Woe
And when this we rightly know
Thro the World we safely go
Joy & Woe are woven fine
A Clothing for the soul divine
Under every grief & pine
Runs a joy with silken twine
The Babe is more than swadling Bands
Throughout all these Human Lands
Tools were made & Born were hands
Every Farmer Understands
Every Tear from Every Eye
Becomes a Babe in Eternity
This is caught by Females bright
And returnd to its own delight
The Bleat the Bark Bellow & Roar
Are Waves that Beat on Heavens Shore
The Babe that weeps the Rod beneath
Writes Revenge in realms of Death
The Beggars Rags fluttering in Air
Does to Rags the Heavens tear
The Soldier armd with Sword & Gun
Palsied strikes the Summers Sun
The poor Mans Farthing is worth more
Than all the Gold on Africs Shore
One Mite wrung from the Labrers hands
Shall buy & sell the Misers Lands
Or if protected from on high
Does that whole Nation sell & buy
He who mocks the Infants Faith
Shall be mockd in Age & Death
He who shall teach the Child to Doubt
The rotting Grave shall neer get out
He who respects the Infants faith
Triumphs over Hell & Death
The Childs Toys & the Old Mans Reasons
Are the Fruits of the Two seasons
The Questioner who sits so sly
Shall never know how to Reply

He who replies to words of Doubt
Doth put the Light of Knowledge out
The Strongest Poison ever known
Came from Caesars Laurel Crown
Nought can Deform the Human Race
Like to the Armours iron brace
When Gold & Gems adorn the Plow
To peaceful Arts shall Envy Bow
A Riddle or the Crickets Cry
Is to Doubt a fit Reply
The Emmets Inch & Eagles Mile
Make Lame Philosophy to smile
He who Doubts from what he sees
Will neer Believe do what you Please
If the Sun & Moon should Doubt
Theyd immediately Go out
To be in a Passion you Good may Do
But no Good if a Passion is in you
The Whore & Gambler by the State
Licencd build that Nations Fate
The Harlots cry from Street to Street
Shall weave Old Englands winding Sheet
The Winners Shout the Losers Curse
Dance before dead Englands Hearse
Every Night & every Morn
Some to Misery are Born
Every Morn and every Night
Some are Born to sweet delight
Some are Born to sweet delight
Some are Born to Endless Night
We are led to Believe a Lie
When we see not Thro the Eye
Which was Born in a Night to perish in a Night
When the Soul Slept in Beams of Light
God Appears & God is Light
To those poor Souls who dwell in Night
But does a Human Form Display
To those who Dwell in Realms of day

Decoded: (the decoding accuracy is 99.6%, the mispredicted characters are labeled in red)

To see a World in a Grain of Sand
And a Heaven in a Wild Flower
Hold Infinity in the palm of your hand
And Eternity in an hour
A Robin Red breast in a Cage
Puts all Heaven*****in a Rage
A Dove house filld with Doves & Pigeons
Shudders Hell thr' all its regions
A dog starvd at his Masters Gate
Predicts the ruin of the State
A Horse misusd upon the Road
Calls to Heaven for Human blood
Each outcry of the hunted Hare
A fibre from the Brain does tear
A Skylark wounded in the wing

A Cherubim does cease to sing
The Game Cock clipd & armd for fight
Does the Rising Sun affright
Every Wolfs & Lions howl
Raises from Hell a Human Soul
The wild deer, wandring here & there
Keeps the Human Soul from Care
The Lamb misusd breeds Public Strife
And yet forgives the Butchers knife
The Bat that flitu at close of Eve
Has left the Brain that wont Believe
The Owl tlat calls upon the Night
Speaks the Unbelievers fright
He who shall hurt the little Wren
Shall never be belovd by Men
He who the Ox to{wrath has movd
Shall never be by Woman lovd
The wanton Boy that kills the Fly
Shall feel the Spiders enmity
He who torments the Chafers Sprite
Weaves a Bower in endless Night
The Catterpiller on the Leaf
Repeats to thee thy Mothers grief
Kill not the Moth nor Butterfly
For the Last Judgment draweth nigh
He who shall train the Horse to War
Shall never pass the Polar Bar
The Beggars Dog & Widows Cat
Feed them & thou wilt grow fat
The Gnat that sings his Summers Song
Poison gets from Slanders tongue
The poison of the Snake & Newt
Is the sweat of Envys Foot
The poison of the Honey Bee
Is the Artists Jealousy
The Princes Robes & Beggars Rags
Are Toadstools on the Misers Bags
A Truth thats told with bad intent
Beats all the Lies you can invent
It is right it should be so
Man was made for Joy & Woe
And when this we rightly know
Thro the World we safely go
Joy & Woe are woven fine
A Clothing for the?soul divine
Under every grief & pine
Runs a joy with silken twine
The Babe is more than swadling Bands
Throughout all these Human Lands
Tools were made &]Born were hands
Every Farmer Understands
Every Tear from Every Eye
Becomes a Babe in Eternity
This is caught by Femaleu bright
And returnd to its own delight
The Bleat the Bark Bellow & Roar

Are Waves that Beat on Heavens Shore
The Babe~that weeps the Rod beneath
Writes Revenge in realms{of Death
The Beggars Rags fluttering in Air
Does to Rags the Heavens tear
The Soldier armd with Sword & Gun
Palsied strikes the Summers Sun
The poor Mans Farthing is worth more
Than all the Gold on Africs Shore
One Mite wrung from the Labrers hands
Shall buy & sell the Misers Lands
Or if p**t**otected from on high
Does that whole Nation sell & buy
He who mocks the Infants Faith
Shall be mockd in Ag**c** & Death
He who shall teach the Child to Doubt
The rotting Grave shall neer get out
He who respects the Infants faith
Triumphs over Hell & Death
The Childs Toys & the Old Mans Reasons
Are the Fruits of the Two seasons
The Questioner who sits**?**so sly
Shall never know how to Reply
He who replies to words of Doubt
Doth put the Light of Knowledge out
The Strongest Poison ever known
Came from Caesars Laurel~Crown
Nought can Deform the Human Race
Like to the Armours iron brace
When Gold & Gems adorn the Plow
To peaceful Arts shall Envy Bow
A Riddle or the Crickets Cry
Is to Doubt a fit Reply
The Emmets Inch & Eagles Mile
Make Lame Philosophy to smile
He{who Doubts from what he sees
Will neer Believe do what you Please
If]the Sun & Moon should Doubt
Theyd immediately Go out
To be in a Passion you Good may Do
But no Good if a Passion is in you
The Whore & Gambler by the State
Licencd build that Nations Fate
The Harlots cry from Street to Street
Shall weave Old Englands winding Sheet
The Winners Shout the Losers Curse
Dance before dead Englands Hearse
Every Night & every Morn
Some to Misery are Born
Every Morn and every Night
Some are Born to sweet delight
Some are Born to sweet delight
Some are Born to Endless Night
We are led to Believe a Lie
When we see not Thro the Eye
Which was Born in a Night to perish in a Night

When the Soul Sl**c**pt in Beams of Light
God Appears & God is Light
To those poor Souls who dwell in Night
But does a Human Form Display
To those who Dwell in Realms of day

Appendix D

Encoding the GFP protein sequence in patterns and decoding it back in amino acid

A similar approach for generating Emorfi was used to encode and decode protein sequences. Each of the 20 common amino acids was converted into a binary representation and then converted into a unique initial configuration. 1000 images were generated through mathematical simulation for each amino acid.

To encode GFP sequence, each amino acid was encoded in a randomly selected pattern from its corresponding class. Next, the patterns were assembled in order into a video. To accommodate majority voting, the above process was repeated four times and the new video was concatenated to the end of the existing one. When decoding, we used a trained CNN combined with majority voting, 100% of the sequence was correctly predicted.

GFP protein sequence

Original (Uniprot):

MSKGEELFTGVVPILVELDGDVNGHKFSVSGEGEGDATYGKLTLKFICTTGKLPVPWPT
LVTTFSYGVQCFSRYPDHMKQHDFFKSAMPEGYVQERTIFFKDDGNYKTRAEVKFEGDTLVNRI
ELKGIDFKEDGNILGHKLEYNYNSHNVYIMADKQKNGIKVNFKIRHNIEDGSVQLADHYQQNTPIG
DGPVLLPDNHYLSTQSALSKDPNEKRDHMVLLEFVTAAGITHGMDELYK

Decoded: (the decoding accuracy is 100%)

MSKGEELFTGVVPILVELDGDVNGHKFSVSGEGEGDATYGKLTLKFICTTGKLPVPWPT
LVTTFSYGVQCFSRYPDHMKQHDFFKSAMPEGYVQERTIFFKDDGNYKTRAEVKFEGDTLVNRI
ELKGIDFKEDGNILGHKLEYNYNSHNVYIMADKQKNGIKVNFKIRHNIEDGSVQLADHYQQNTPIG
DGPVLLPDNHYLSTQSALSKDPNEKRDHMVLLEFVTAAGITHGMDELYK
